# CB[7]- and CB[8]-Based [2]-(Pseudo)rotaxanes with Triphenylphosphonium-Capped Threads: Serendipitous Discovery of a New High-Affinity Binding Motif

**DOI:** 10.1021/acs.orglett.2c01028

**Published:** 2022-05-06

**Authors:** Iago Neira, Carlos Peinador, Marcos D. García

**Affiliations:** Departamento de Química and Centro de Investigaciones Científicas Avanzadas (CICA). Facultad de Ciencias, Universidade da Coruña, 15071 A Coruña, Spain

## Abstract

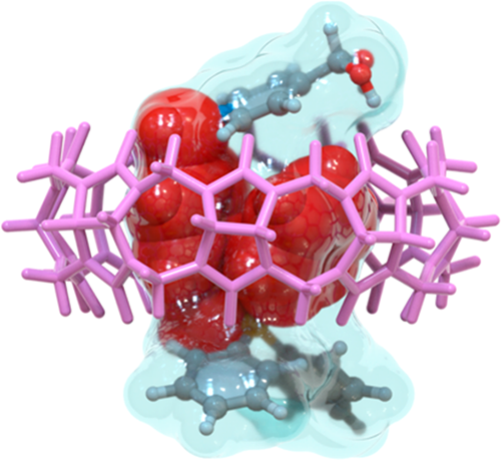

The synthesis of new triphenylphosphonium-capped cucurbit[7]uril (CB[7])- and cucurbit[8]uril (CB[8])-based [2]rotaxanes was achieved by a simultaneous threading-capping strategy. While the use of CB[7] produced the designed [2]rotaxane, attempts to obtain the CB[8] analogue were unsuccessful due to the unexpected strong interaction found between the host and the phosphonium caps leading to pseudo-heteroternary host–guest complexes. This unusual binding motif has been extensively studied experimentally, with results in good agreement with those obtained by dispersion-corrected DFT methods.

Mechanically interlocked molecules (MIMs)^1,2^ are no longer a chemical curiosity but a solid platform for the development of new functionality.^3^ Nevertheless, the efficient synthesis of these entities is still challenging, especially when restricted to aqueous media and the limited choice of intermolecular interactions and reactivity that can be used in this setting.^4^ Cucurbit[*n*]urils (CB[*n*]s, *n* = 5–8, 10, 13–15)^5^ have substantially eased this problem, enlarging the toolbox for the preparation of MIMs in aqueous media,^6^ in particular, when using well-developed synthetic strategies that employ premade axle components (e.g., capping^7^ or slipping^8^). Water-soluble CB[*n*]s are commercially available, nontoxic, and fairly nonreactive and own flexible inner hydrophobic cavities of different sizes, and their host–guest chemistry is mainly directed by cation–dipole interactions,^9^ the hydrophobic effect^10^ and optimization of the host–guest packing coefficient.^11,12^ Furthermore, in the case of CB[8], its polar and large hydrophobic cavity, 1.7 times the volume of CB[7], allows for the preparation of unusual 1:2 heteroternary complexes with aromatic guests of complementary electron acceptor/donor nature, which are stabilized within the cavity by increased charge-transfer interactions.^13^

Following our interest in the chemistry of CB[*n*]s^14^ and pyridinium salts,^15^ we designed the synthetic strategy for the construction of CB[7]- and CB[8]-based asymmetric [2]-rotaxanes depicted in Scheme 1c, having as key steps (a) the threading of CB[7] and CB[8] into complementary triphenylphosphonium-capped semidumbbells **1**_**a**_^2+^/**1**_**b**_^2+^ and (b) trapping of the interlocked molecule by an unusual kinetically controlled imine bonding reaction recently developed by our group.^16^

**Scheme 1:**
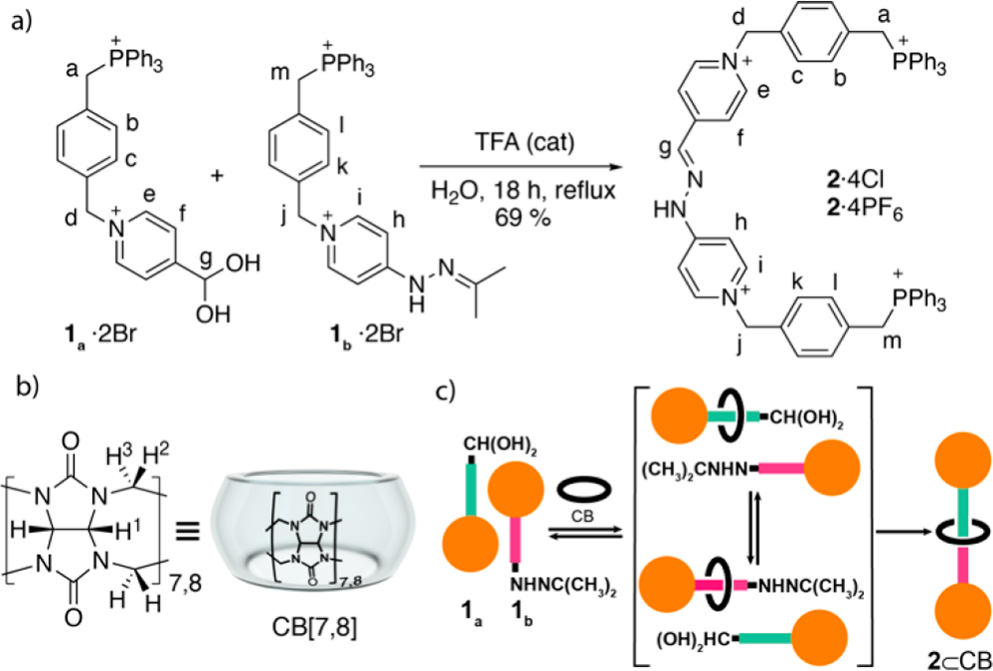
(a) Synthesis of Thread **2**^4+^; (b) Schematic Representation of CB[7] and CB[8]; and (c) Planned Synthesis of Rotaxanes **2**^**4+**^⊂CB[7] and **2**^**4+**^⊂CB[8] by Simultaneous Threading–Capping

Building blocks **1**_**a**_·2Br and **1**_**b**_·2Br were efficiently prepared by substitution reactions of commercially available (4-(bromomethyl)benzyl)triphenylphosphonium bromide with, respectively, isonicotinaldehyde and 4-hydrazinylpyridine.^17^ We then tested the assembly of the axle component **2**^4+^ (Scheme 1) by reacting **1**_**a**_^2+^ and **1**_**b**_^2+^ for 18 h in refluxing water and in the presence of a catalytic amount of TFA. The process produced the expected thread, which was isolated after column chromatography as **2**·4Cl in 69% yield and fully characterized by means of ^31^P/^1^H/^13^C 1D/2D NMR and ESI-MS.^17^ As previously demonstrated for related hydrazones,^16^ cation **2**^4+^ displayed an abnormal stability, with no signs of hydrolysis being detected over a period of weeks.^17^ Having the assembled thread in our hands, we tested a slipping approach for the synthesis of **2**^**4+**^⊂CB[7]^8^ by heating equimolar 1.0 mM mixtures of **2**·4Cl and CB[7] in D_2_O. This procedure failed to produce the MIM, validating therefore the use of the triphenylphosphonium groups as suitable stoppers for CB[7]-based rotaxanes.^16c^

As originally intended, we alternatively undertook the synthesis of **2**^4+^⊂CB[7] starting from the host and the individual components **1**_**a**_^**2+**^ and **1**_**b**_^**2+**^(Scheme 1). Nevertheless, we first studied the CB[7]-guest complexation processes by following by ^1^H/^31^P NMR the reactions between 1.0 mM solutions in D_2_O, of either **1**_**a**_^2+^ or **1**_**b**_^2+^ and increasing amounts of the host.^17^ In both cases, the data recorded were in good agreement with the formation of the expected 1:1 host–guest complexes **1**_**a**_^2+^/**1**_**b**_^2+^⊂CB[7], which appear in the spectra in a situation of rapid exchange in the NMR time scale. Further 1D/2D NMR and HR ESI-MS experiments additionally validated the formation of the pseudorotaxanes, and *K*_a_ = 1.46 ± 0.07 × 10^5^ M^–1^ could be estimated for **1**_**b**_^**2+**^⊂CB[7] through an UV–vis titration experiment, which showed a good fitting of the data to a 1:1 binding isotherm (Figure 1c).

**Figure 1:**
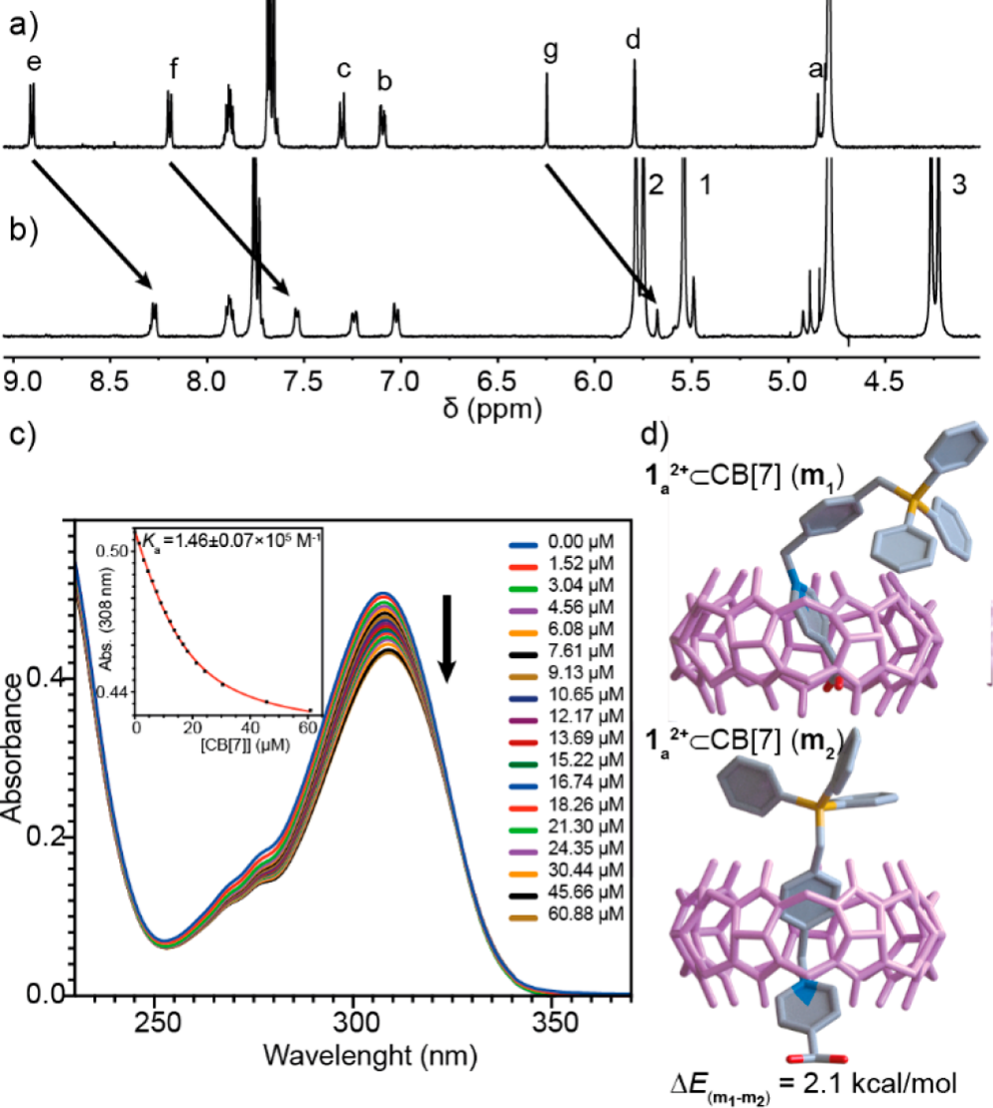
Partial ^1^H NMR (500 MHz, D_2_O) spectrum of (a) 1 mM solution of **1**_**a**_^2+^ and (b) 1 mM of **1**_**a**_^**2+**^ + 1 equiv of CB[7]. (c) Partial UV–vis and titration data (inset) for **1**_**b**_^2+^ (15 μM) in the presence of increasing concentrations of CB[7] in water. The red line in the inset shows the fit to a 1:1 binding model. (d) Schematic representation of the two energy minima **m**_**1**_ and **m**_**2**_ found for **1**_**a**_^2+^⊂CB[7] using DFT methods.^17−19^

Surprisingly, despite the semidumbbells having xylylene-based hydrophobic cores flanked with two positive charges, the binding sites observed in both cases for the CB[7] host were not these moieties^14c^ but, instead, the corresponding pyridinium rings. For instance, the ^1^H NMR signals corresponding to the pyridinium ring in **1**_**a**_^2+^ appear significantly deshielded (ΔδH_f_ = 0.62 ppm and ΔδH_e_ = 0.63 ppm, Figure 1a,b), while H_b,c_ within the xylylene core are only slightly altered. Furthermore, the ^31^P NMR signal for **1**_**a**_^2+^ was also downshifted ΔδP_a_ = 0.73 ppm, in good agreement with the discussed insertion mode.^17^ Dispersion-corrected calculations carried out for **1**_**a**_^2+^⊂CB[7] supported this end,^17−21^ being the local minimum **m**_**1**_ found for the insertion of the pyridyl group within CB[7], 2.1 kcal/mol more stable than that corresponding to the inclusion of the xylylene moiety (**m**_**2**_, Figure 2d).

**Figure 2:**
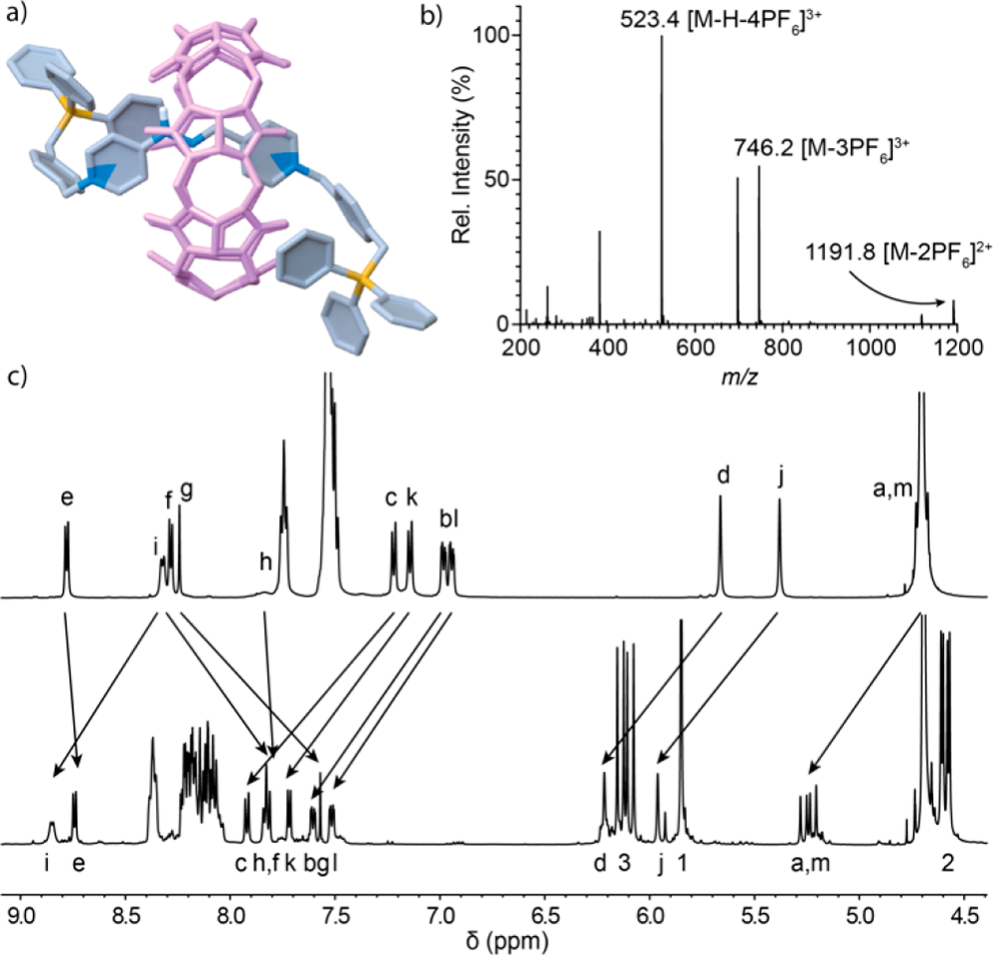
Rotaxane **2**^**4+**^⊂CB[7]: (a) DFT-optimized geometry; (b) LR ESI-MS spectrogram for the hexafluorophosphate salt; (c) partial ^1^H NMR (500 MHz, D_2_O) of (top) axle **2**^4+^ and (bottom) **2**^4+^⊂CB[7].

Once both the formation of **1**_**a**_^2+^/**1**_**b**_^2+^⊂CB[7] and the axle **2**^4+^ were firmly established, we undertook the synthesis of the [2]rotaxane **2**^**4+**^⊂CB[7]. Hence, the very same conditions used for the synthesis of **2**·4Cl were applied, but in the presence of 2 equiv of CB[7]. After 18 h, the expected CB[7]-based MIM was isolated as **2**·4PF_6_⊂CB[7] in 55% yield, being thoroughly characterized by ^31^P/^13^C/^1^H NMR and ESI-MS (Figure 2b).^17^ Additionally, an analytical sample of **2·**4Cl⊂CB[7] could be obtained by anion metathesis,^17^ allowing the characterization of the [2]rotaxane in aqueous media by 1D/2D NMR techniques. In particular, the ^1^H spectrum in D_2_O showed an appropriate integration of the asymmetric protons for CB[7] in relation with the axle nuclei, with chemical shifts for H_f-h_ consistent with the expected shielding caused by the positioning of the central bis-pyridinium moiety within the host (Figure 2c). This later observation was also found in good agreement with the DFT-optimized geometry obtained for a local minimum of the [2]rotaxane (Figure 2a).^17−21^ DOSY NMR also supported the formation of the MIM, with all of the resonances for the compound diffusing as a whole on the recorded spectrum (see Figure S5).

Continuing with our study, we then tackled the synthesis of the CB[8] analogue of the [2]rotaxane, first by employing again a slipping approach between thread **2**^4+^ and the host. In this case, sonication of a 2.0 mM solution of **2**^4+^ in D_2_O with excess macrocycle led to unexpected results: not only the relative integration of the CB[8] signals on the ^1^H NMR showed two units of the host strongly interacting with the axle, but the whole species displayed a sole diffusion coefficient on the corresponding DOSY experiment (Figure 3c). Assignment of the ^1^H nuclei could be carried out based on 2D NMR experiments,^17^ as H_a–d,j–m_ on the xylene moieties consistently shielded compared to the free axle, in good agreement with their inclusion within the hydrophobic cavity of CB[8]. Moreover, the signals for the six phenyl rings on the two nonequivalent R–P^+^Ph_3_ groups on **2**^4+^ appeared, in turn, divided into two very differently affected sets of signals on the ^1^H spectrum, slowly exchanging on the NMR time scale. In essence, 20 hydrogens accounting for two of the phenyl rings on each moiety appear slightly deshielded, as would be expected for an interaction of those with the outer rims of the CB[8]s. On the other hand, the remaining 10 nuclei (H_*x*–*z*/*x*′–*z*′_) appear significantly shielded by ca. 1 ppm, a situation that would imply their surprising inclusion within the cavity of the macrocycles forming an heteroternary binding motif in conjunction with the xylyl moiety. In contraposition with what discussed above for **2**^4+^⊂CB[7], the chemical shifts observed in this case for the nuclei of the pseudoviologen moiety appear more erratically affected by CB[8], as it would derive from their positioning outside of the host. Moreover, ^31^P NMR showed as well data consistent with the binding of the host to the phosphonium ends of the nonsymmetric axle (ΔδP = −1.61 and −1.77 ppm). Finally, ESI-MS experiments could be recorded for the pseudorotaxane **2·**4Cl⊂(CB[8])_2_, showing a clear peak for the proposed complex **2**^**4+**^⊂(CB[8])_2_ at *m*/*z* 897.0462 (calcd 897.0461) (Figure 3b).

**Figure 3:**
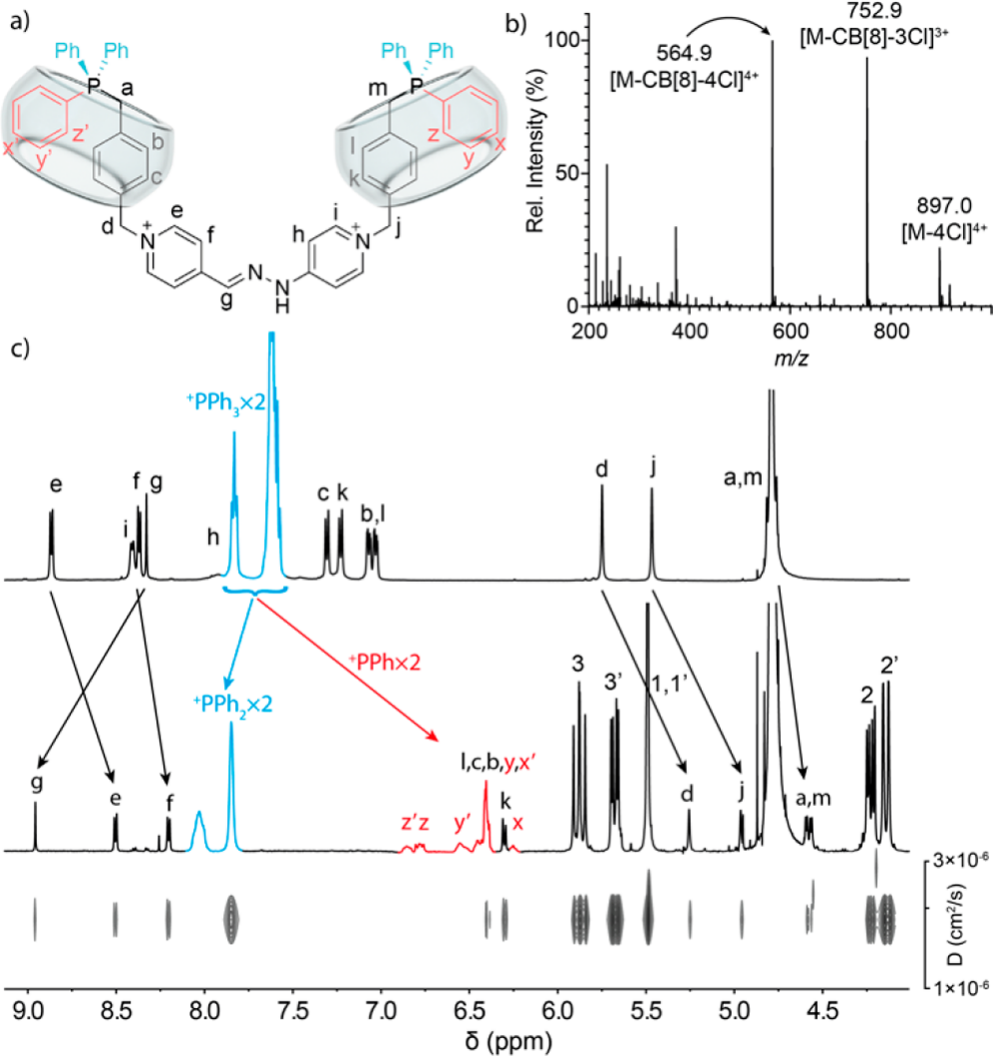
Pseudo[3]rotaxane **2**^4+^⊂(CB[8])_2_: (a) schematic representation; (b) LR ESI-MS spectrogram for the chloride salt; (c) partial ^1^H NMR (500 MHz, D_2_O) of (top) axle **2**^4+^, (middle) 2 mM **2**^4+^ + 2 equiv of CB[8], and (bottom) DOSY experiment for the precedent solution.

To further validate our hypothesis of the CB[8] host introducing within its cavity two of the four aromatic rings of the benzyltriphenylphosphonium moiety, we proceed to study the interaction of **1**_**a**_^2+^ and **1**_**b**_^2+^ with the macrocycle in aqueous media. Hence, we recorded the ^1^H NMR spectrum of equimolar 2 mM solutions of the guests, saturating with excess CB[8] by sonication.^17^ These experiments showed clear indications of interaction between the components, showing nearly the same patterns of integration, splitting of the CB[8] signals and complexation induced shifts, discussed above for the pseudo[3]rotaxane **2**^4**+**^⊂(CB[8])_2_. HR ESI-MS experiments further confirmed the identity of the **1**_**a**_^2+^/**1**_**b**_^2+^⊂CB[8] 1:1 complexes, showing diagnostic peaks for [**1**_**a**_^**2+**^⊂CB[8]]^2+^ at *m*/*z* 909.7973 (calcd 909.7965), and for [**1**_**b**_^2+^⊂CB[8]]^2+^ at *m*/*z* 901.8046 (calcd 901.8064). Interestingly, 2D ROESY NMR experiment recorded for **1**_**a**_^2+^/**1**_**b**_^2+^⊂CB[8] allowed us to obtain further information, as we observed three EXSY exchange peaks associated to each of the nonequivalent protons of the phenyl groups, implying its slow exchange *in* and *out* of the macrocycle in the NMR time scale (Figure 4). Consequently, the energy barrier for the exchange (Δ*G*^⧧^) could be estimated from VT-NMR experiments on both **1**_**a**_^2+^/**1**_**b**_^2+^⊂CB[8] (see Figures S8 and S9), showing similar values of approximately 15.0 kcal/mol. Finally, the strength of the association could be estimated in the case of **1**_**a**_^2+^⊂CB[8], by using NMR competitive experiments with (trimethylammonium)methylferrocene as a standard for the calculation (log*K*_a_ = 9.49).^4,17^ Thus, the host–guest interaction was observed to be stronger than that of the standard, and that the process was under no kinetic barriers, showing a quite impressive binding constant *K*_a_ = (3.6 ± 0.7)·10^10^ M^–1^, comparable to that found to other high-affinity substrates such as adamantane derivatives.^22^

**Figure 4:**
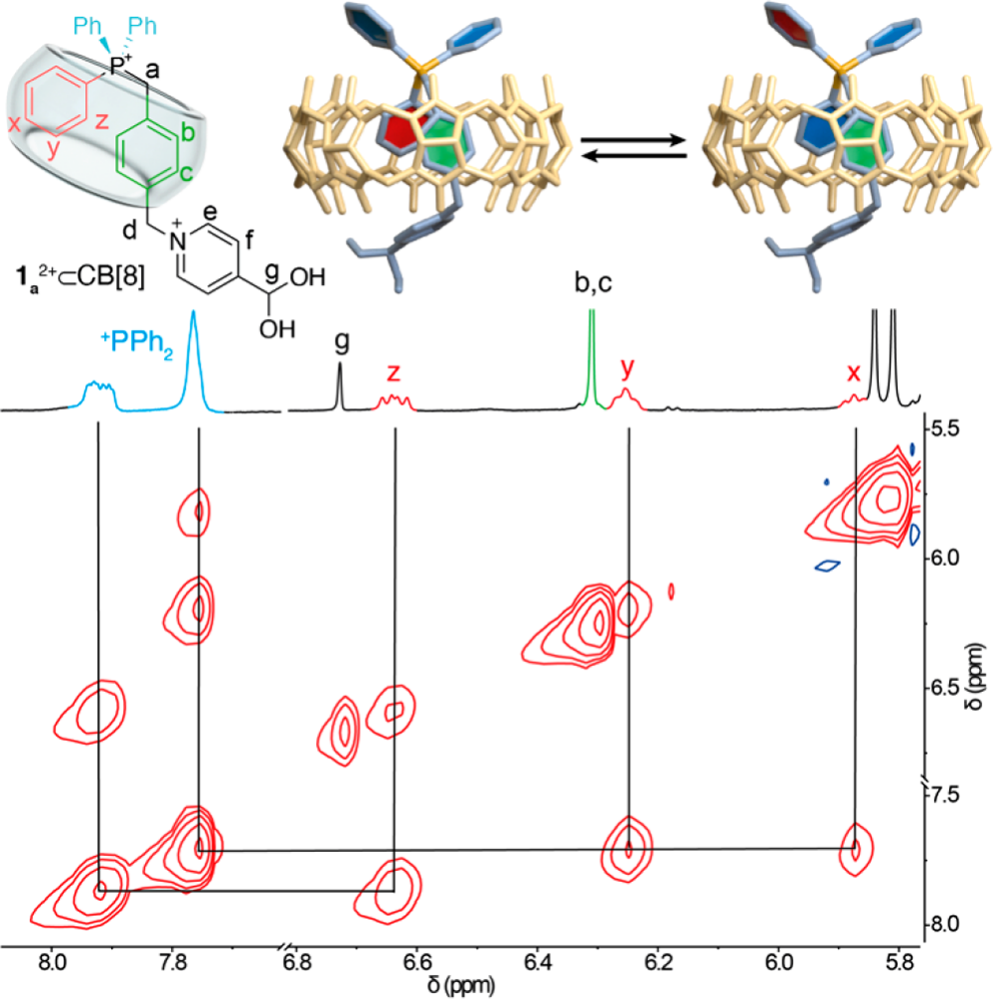
Partial 2D ROESY NMR (500 MHz, D_2_O) showing EXSY exchange cross peaks between phenyl groups in the complex **1**_**a**_^2+^⊂CB[8].

To gain more insight on the structural characteristics of these atypical heteroternary complexes, DFT calculations were carried out on **1**_**a**_^**2+**^⊂CB[8]. Among the four local minima found on the potential energy surface for the pseudorotaxane (**m′**_**1**–**4**_, Figure 5),^17^ the two lowest energy conformers show the proposed heteroternary binding mode, differing on the *syn*- (**m′**_**1**_) or *anti*- (**m′**_**2**_) relative disposition of the pyridinium moiety and the inserted phenyl ring, and being separated by Δ*E*= *E***m′**_**1**_ – *E***m′**_**2**_ = 7.7 kcal/mol. Furthermore, the two other minima identified for the complex display the same binding modes discussed before for **1**_**a**_^**2+**^⊂CB[7], but being in this case significantly higher in relative energy (Δ*E* = *E***m′**_**1**_-*E***m′**_**4**_ = 22.7 kcal/mol, Δ*E* = *E***m′**_**1**_ – *E***m′**_**3**_ = 19.3 kcal/mol). As can be seen in Figure 5, not only is the cavity of the macrocycle able to accommodate one of the phenyl rings and the benzyl group of **1**_**a**_^2+^ but also this insertion mode significantly optimizes the occupied volume with respect to the large volume of the CB[8] cavity with a p.c. of 58%, very close to the optimal occupancy value.^12,13^ Furthermore, the two positive charges of the binding motif in **m′**_**1,2**_ (N^+^ and P^+^) are located approximately in the center of the polygons defined by the oxygen atoms flanking the CB[8] portals, somehow optimizing as well the cation–dipole interactions.

**Figure 5:**
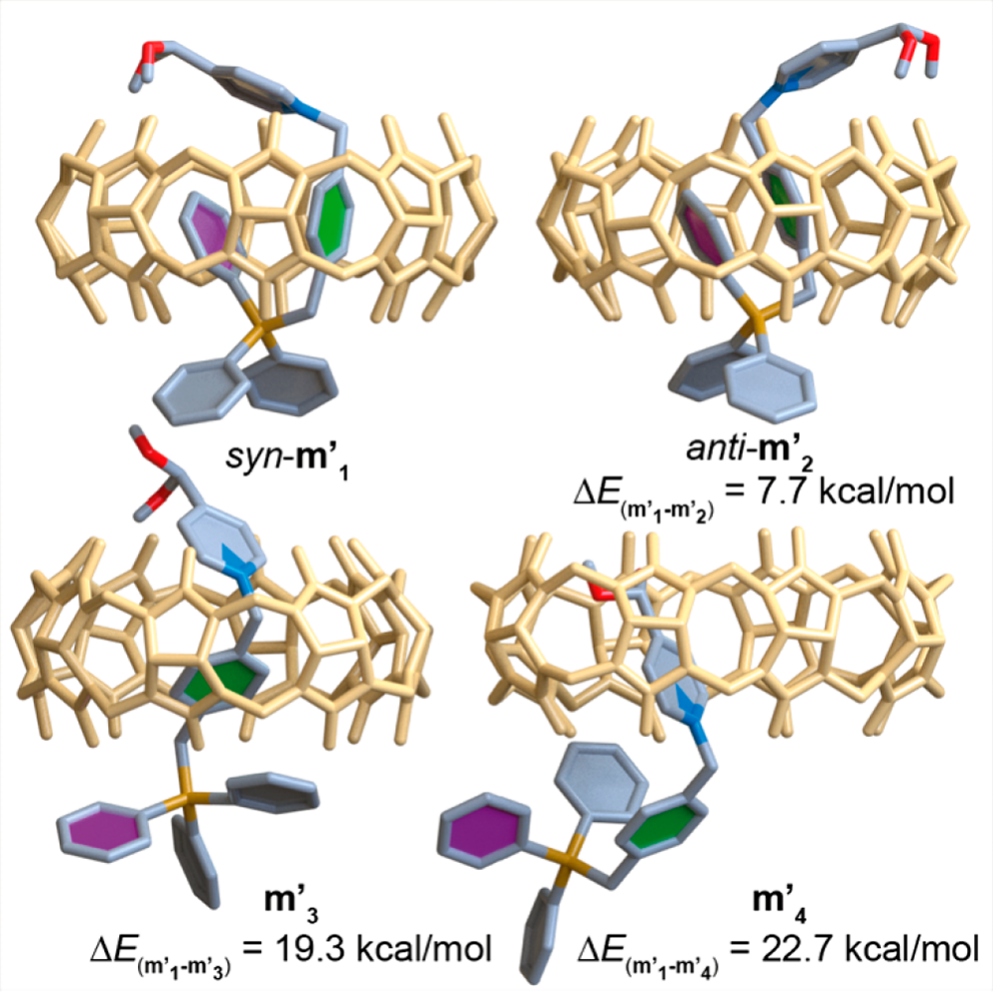
Schematic representation of the four energy minima **m′**_**1**_**-m′**_**4**_ found for complexes **1**_**a**_^2+^⊂CB[8] using DFT methods.^17−19^

In summary, we have discussed here our new results on the synthesis of tryphenylphosphonium-capped CB[7]- and CB[8]-based rotaxanes. The intended strategy has been studied in detail, planned to concomitantly produce CB[7] and CB[8] complexes with complementary aldehyde and hydrazone reactive ends, and their covalent irreversible junction by imine bond formation of the thread component on the MIM. While the method efficiently produced the expected CB[7]-based [2]-rotaxane, the use of CB[8] as a wheel component resulted in the formation of unexpected pseudo-heteroternary complexes between the host and the capped ends of the thread. This unusual result has been studied in depth, with the experimental data and dispersion-corrected DFT calculations being in excellent agreement with the proposed binding mode. The reported results open the door not only for the development of new water-soluble rotaxanes but also for the design of new high-affinity binding guests for CB[8] inspired by the pseudo-heteroternary complexation mode discussed herein.^22,23^

## Supporting Information Available

Experimental procedures, characterization data for new compounds, additional figures and computational details including the Cartesian coordinates for the DFT structures discussed in the text (PDF)
