# Supplementary material for: CB[7]- and CB[8]-Based [2]-(Pseudo)rotaxanes with Triphenylphosphonium-Capped Threads: Serendipitous Discovery of a New High-Affinity Binding Motif
Source: Org Lett. 2022 May 6;24(25):4491–5. doi: 10.1021/acs.orglett.2c01028 (PMC9251766; doi:10.1021/acs.orglett.2c01028)
Supplement: Supplementary file 1 — ol2c01028_si_001.pdf [file ol2c01028_si_001.pdf]

Supporting information

CB[7] and CB[8]-based [2]-(pseudo)rotaxanes with triphenylphosphonium-capped threads: Serendipitous discovery of a new high-affinity binding motif.

Iago Neira, Carlos Peinador*, Marcos D. García*

Departamento de Química and Centro de Investigaciones Científicas Avanzadas (CICA),

Facultad de Ciencias, Universidade da Coruña, 15071, A Coruña, Spain.

Content:

General S5

SP1: Synthesis of precursor P1·Br S5

SP2: Synthesis of semiaxle 1_a·2Br.. S6

SP3: Synthesis of semiaxle 1_b·2Br.. S7

SP4: Synthesis of axle 2·4Cl S8

SP5: Synthesis of pseudorotaxane 1_a·2Br⊂CB[7].. S9

SP6: Synthesis of pseudorotaxane 1_b·2Br ⊂CB[7].. S10

SP7: Synthesis of rotaxane 2·4PF_6⊂CB[7]: S11

SP8: Synthesis of pseudorotaxane 1_a·2Br⊂CB[8]:.. S13

SP9: Synthesis of pseudorotaxane 1_b·2Br⊂CB[8]:.. S14

SP10: Synthesis of [3]pseudorotaxane 2·4Cl⊂$(CB[8])_2$: S15

NMR titrations of semiaxles 1_a·2Br and 1_b·2Br with CB[7]:.. S16

DOSY of 2·4PF_6⊂CB[7]:.. S18

Calculation of Host-Guest Binding Constant by Competitive 1H NMR spectroscopy with ferrocenylmethyl trimethylammonium iodide (Fc·I): S19

Determination of the energy of the exchange barrier ($\Delta G^{\neq}$): S22

Computational details:.. S24

NMR spectra:.. S43

Figure S1 Partial ^{1}H NMR (400 MHz, D_2O): (a) 1 mM of semiaxle $\mathbf{1_a}$·2Br, (b) 1 mM of semiaxle $\mathbf{1_a}$·2Br + 0.5 eq of CB[7] and (c) 1 mM of semiaxle $\mathbf{1_a}$·2Br + 1.0 eq of CB[7]. S16
Figure S2 Partial $^{31}P\{^{1}H\}$ NMR (160 MHz, D_2O): (a) 1 mM of semiaxle $\mathbf{1_a}$·2Br, (b) 1 mM of semiaxle $\mathbf{1_a}$·2Br + 0.5 eq of CB[7] and (c) 1 mM of semiaxle $\mathbf{1_a}$·2Br + 1.0 eq of CB[7]. S16
Figure S3 Partial ^{1}H NMR (400 MHz, D_2O): (a) 1 mM of semiaxle $\mathbf{1_b}$·2Br, (b) 1 mM of semiaxle $\mathbf{1_b}$·2Br + 0.5 eq of CB[7] and (c) 1 mM of semiaxle $\mathbf{1_b}$·2Br + 1.0 eq of CB[7]. S17
Figure S4 Partial ^{31}P NMR (160 MHz, D_2O): (a) 1 mM of semiaxle $\mathbf{1_b}$·2Br, (b) 1 mM of semiaxle $\mathbf{1_b}$·2Br + 0.5 eq of CB[7] and (c) 1 mM of semiaxle $\mathbf{1_b}$·2Br + 1.0 eq of CB[7]. S17
Figure S5 DOSY NMR (500 MHz, CD_3CN) of **2**·4PF_6⊂CB[7] S18
Figure 6. Partial ^{1}H NMR spectrum (500 MHz, D_2O) of: (a) 1mM solution of $\mathbf{Fc^+}$ and (b) 1mM solution of $\mathbf{Fc^+}$⊂CB[8] complex. S19
Figure S7 Partial ^{1}H NMR spectrum (500 MHz, D_2O) of 500 μL of 1.00 mM solution of $\mathbf{1_a^{2+}}$⊂CB[8] complex with 250 μL of 5.00 mM solution of $\mathbf{Fc^+}$. S20
Figure S8 Partial ^{1}H NMR spectrum (500 MHz, D_2O) of 500 μL of 1.00 mM solution of $\mathbf{Fc^+}$⊂CB[8] complex with 200 μL of 2.00 mM solution of $\mathbf{1_a}^{2+}$. S21
Figure S9 Partial ^{1}H NMR spectra (500 MHz, D_2O) of $\mathbf{1_a^{2+}}$⊂CB[8] at different temperatures. S22
Figure S10 Partial ^{1}H NMR spectra (500 MHz, D_2O) of $\mathbf{1_b^{2+}}$⊂CB[8] at different temperatures. S23
Figure S11. ^{1}H NMR (500 MHz, D_2O) spectrum of **P1**·Br. S43
Figure S12 $^{13}C\{^{1}H\}$ NMR (125 MHz, D_2O) spectrum of **P1**·Br. S43
Figure S13 $^{13}C\{^{1}H\}$ and DEPT 135$\{^{1}H\}$ NMR (125 MHz, D_2O) spectrum of **P1**·Br. S44
Figure S14 COSY (500 MHz, D_2O) spectrum of **P1**·Br. S44
Figure S15 HSQC (500 MHz, D_2O) spectrum of **P1**·Br. S45
Figure S16 HMBC (500 MHz, D_2O) spectrum of **P1**·Br. S45
Figure S17 $^{31}P\{^{1}H\}$ (160 MHz, D_2O) spectrum of **P1**·Br. S46
Figure S18 ^{1}H NMR (500 MHz, D_2O) spectrum of **1a**·2Br. S46
Figure S19 $^{13}C\{^{1}H\}$ NMR (125 MHz, D_2O) spectrum of **1a**·2Br. S47
Figure S20 $^{13}C\{^{1}H\}$ and DEPT 135$\{^{1}H\}$ NMR (125 MHz, D_2O) spectrum of **1a**·2Br. S47
Figure S21 COSY (500 MHz, D_2O) spectrum of **1a**·2Br. S48
Figure S22 HSQC (500 MHz, D_2O) spectrum of **1a**·2Br. S48
Figure S23 HMBC (500 MHz, D_2O) spectrum of **1a**·2Br. S49
Figure S24 ^{31}P (500 MHz, D_2O) spectrum of **1a**·2Br. S49
Figure S25 ^{1}H NMR (500 MHz, D_2O) spectrum of **1b**·2Br. S50
Figure S26 $^{13}C\{^{1}H\}$ NMR (125 MHz, D_2O) spectrum of **1b**·2Br. S50
Figure S27 $^{13}C\{^{1}H\}$ and DEPT 135$\{^{1}H\}$ NMR (125 MHz, D_2O) spectrum of **1b**·2Br. S51
Figure S28 COSY (500 MHz, D_2O) spectrum of **1b**·2Br. S51
Figure S29 HSQC (500 MHz, D_2O) spectrum of **1b**·2Br. S52
Figure S30 HMBC (500 MHz, D_2O) spectrum of **1b**·2Br. S52
Figure S31 $^{31}P\{^{1}H\}$ NMR (160 MHz, D_2O) spectrum of **1b**·2Br. S53
Figure S32 ^{1}H NMR (500 MHz, D_2O) spectrum of **2**·4Cl. S53
Figure S33 $^{13}C\{^{1}H\}$ NMR (500 MHz, D_2O) spectrum of **2**·4Cl. S54
Figure S34 $^{13}C\{^{1}H\}$ and DEPT 135$\{^{1}H\}$ NMR (500 MHz, D_2O) spectrum of **2**·4Cl. S54
Figure S35 COSY (500 MHz, D_2O) spectrum of **2**·4Cl. S55
Figure S36 HSQC (500 MHz, D_2O) spectrum of **2**·4Cl. S55
Figure S37 HMBC (500 MHz, D_2O) spectrum of **2**·4Cl. S56

Figure S38 $^{31}P\{^1H\}$ NMR (160 MHz, D_2O) spectrum of **2**·4Cl............ S56
Figure S39 ^1H NMR (500 MHz, D_2O) spectrum of $\mathbf{1_a}$·2Br⊂CB[7]. S57
Figure 40. $^{13}C\{^1H\}$ NMR (500 MHz, D_2O) spectrum of $\mathbf{1_a}$·2Br⊂CB[7]. S57
Figure 41. $^{13}C\{^1H\}$ and DEPT 135$\{^1H\}$ NMR (500 MHz, D_2O) spectrum of $\mathbf{1_a}$·2Br⊂CB[7]. S58
Figure S42 $^{31}P\{^1H\}$ NMR (160 MHz, D_2O) spectrum of $\mathbf{1_a}$·2Br⊂CB[7]. S58
Figure S43 ^1H NMR (500 MHz, D_2O) spectrum of $\mathbf{1_b}$·2Br⊂CB[7] S59
Figure 44 $^{13}C\{^1H\}$ NMR (500 MHz, D_2O) spectrum of $\mathbf{1_b}$·2Br⊂CB[7]. S59
Figure 45 $^{13}C\{^1H\}$ and DEPT 135$\{^1H\}$ NMR (500 MHz, D_2O) spectrum of $\mathbf{1_b}$·2Br⊂CB[7]. S60
Figure S46 $^{31}P\{^1H\}$ NMR (160 MHz, D_2O) spectrum of $\mathbf{1_b}$·2Br⊂CB[7]. S60
Figure S47 ^1H NMR (500 MHz, CD_3CN) spectrum of **2**·4PF_6. .... S61
Figure S48 $^{13}C\{^1H\}$ NMR (125 MHz, CD_3CN) spectrum of **2**·4PF_6.⊂CB[7]. S61
Figure S49 $^{13}C\{^1H\}$ and DEPT 135$\{^1H\}$ NMR (125 MHz, CD_3CN) spectrum of **2**·4PF_6.⊂CB[7]. S62
Figure S50 COSY (500 MHz, CD_3CN) spectrum of **2**·4PF_6.⊂CB[7]. S62
Figure S51 HSQC(500 MHz, CD_3CN) spectrum of **2**·4PF_6.⊂CB[7]. S63
Figure S52 HMBC (500 MHz, CD_3CN) spectrum of **2**·4PF_6.⊂CB[7]. S63
Figure S53 ^{31}P NMR (160 MHz, CD_3CN) spectrum of **2**·4PF_6.⊂CB[7]. S64
Figure S54 ^1H NMR (500 MHz, D_2O) spectrum of **2**·4Cl⊂CB[7]. S64
Figure S55 $^{13}C\{^1H\}$ NMR (125 MHz, D_2O) spectrum of **2**·4Cl⊂CB[7] S65
Figure S56 $^{13}C\{^1H\}$ and DEPT 135$\{^1H\}$ NMR (125 MHz, D_2O) spectrum of **2**·4Cl⊂CB[7] S65
Figure S57 $^{31}P\{^1H\}$ NMR (160 MHz, D_2O) spectrum of **2**·4Cl⊂CB[7]. S66
Figure S58 ^1H NMR (500 MHz, D_2O) spectrum of **2**·4Cl⊂$(CB[8])_2$. S66
Figure S59 $^{13}C\{^1H\}$ NMR (125 MHz, D_2O) spectrum of **2**·4Cl⊂$(CB[8])_2$. S67
Figure S60 $^{13}C\{^1H\}$ and DEPT 135$\{^1H\}$ NMR (125 MHz, D_2O) spectrum of **2**·4Cl⊂$(CB[8])_2$. S67
Figure S61 COSY (500 MHz, D_2O) spectrum of **2**·4Cl⊂$(CB[8])_2$. S68
Figure S62 HSQC (500 MHz, D_2O) spectrum of **2**·4Cl⊂$(CB[8])_2$. S68
Figure S63 HMBC (500 MHz, D_2O) spectrum of **2**·4Cl⊂$(CB[8])_2$. S69
Figure S64 $^{31}P\{^1H\}$ NMR (160 MHz, D_2O) spectrum of **2**·4Cl⊂$(CB[8])_2$. S69
Figure S65 ^1H NMR (500 MHz, D_2O) spectrum of $\mathbf{1_a}$·2Br ⊂CB[8]. S70
Figure S66 $^{13}C\{^1H\}$ NMR (125 MHz, D_2O) spectrum of $\mathbf{1_a}$·2Br ⊂CB[8]. S70
Figure S67 $^{13}C\{^1H\}$ and DEPT 135$\{^1H\}$ NMR (125 MHz, D_2O) spectrum of $\mathbf{1_a}$·2Br ⊂CB[8] S71
Figure S68 COSY (125 MHz, D_2O) spectrum of $\mathbf{1_a}$·2Br ⊂CB[8] S71
Figure S69 HSQC (125 MHz, D_2O) spectrum of $\mathbf{1_a}$·2Br ⊂CB[8]. S72
Figure S70 HMBC (125 MHz, D_2O) spectrum of $\mathbf{1_a}$·2Br ⊂CB[8]. S72
Figure S71 $^{31}P\{^1H\}$ NMR (125 MHz, D_2O) spectrum of $\mathbf{1_a}$·2Br ⊂CB[8]. S73
Figure S72 ^1H NMR (500 MHz, D_2O) spectrum of $\mathbf{1_b}$·2Br ⊂CB[8]. S73
Figure S73 $^{13}C\{^1H\}$ NMR (500 MHz, D_2O) spectrum of $\mathbf{1_b}$·2Br ⊂CB[8]. S74
Figure S74 $^{13}C\{^1H\}$ and DEPT 135$\{^1H\}$ NMR (500 MHz, D_2O) spectrum of $\mathbf{1_b}$·2Br ⊂CB[8]. S74
Figure S75 COSY (500 MHz, D_2O) spectrum of $\mathbf{1_b}$·2Br ⊂CB[8]. S75
Figure S76 HSQC (500 MHz, D_2O) spectrum of $\mathbf{1_b}$·2Br ⊂CB[8]. S75
Figure S77 HMBC (500 MHz, D_2O) spectrum of $\mathbf{1_b}$·2Br ⊂CB[8]. S76
Figure S78 $^{31}P\{^1H\}$ NMR (500 MHz, D_2O) spectrum of $\mathbf{1_b}$·2Br ⊂CB[8]. S76

General

The chemicals used in this work were purchased from commercial suppliers and used without further purification. The purity of the CB[7] and CB[8] was assessed as previously reported by Kaifer *et al.*[1] Milli-Q water was purified with a Millipore Gradient A10 apparatus. Merck 60 F254 foils were used for thin layer chromatography, and Merck 60 (230-400 mesh) silica gel was used for flash chromatography. NMR spectra were recorder on a Bruker Advance 400 or 500 MHz for ^{1}H, 125 MHz for ^{13}C equipped each other with a dual cryoprobe and 160 MHz for ^{31}P. The solvent for NMR experiments was deuterated water (D_2O) and acetonitrile (CD_3CN). Mass spectrometry experiments were carried out in a LCQ-q-TOF Applied Biosystems QSTAR Elite spectrometer for low and high resolution ESI. UV/Vis spectra were recorded on a Jasco V-650 spectrometer. Structural assignments were made with additional information from gCOSY, gHSQC, and gHMBC experiments.

SP1: Synthesis of precursor P1·Br

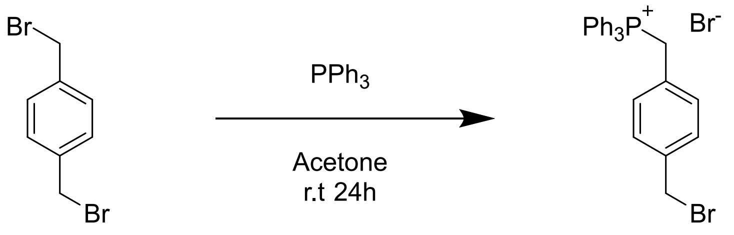

P1·Br

A mixture of 1,4-bis(bromomethyl)benzene (1.00 g, 3.78 mmol) and triphenylphosphine (1.00 g, 3.78 mmol) in 40 mL of acetone was left under stirring for 24 hours at room temperature. Then, the solvent was removed in vacuum to leave a white solid residue, which was subjected to flash chromatography (SiO_2, solvent: CH_2Cl_2/MeOH 9:1). The precursor containing fractions were combined and evaporated to yield **P1**·Br as a white solid (1.72 g, 86%).

^{1}H NMR (500 MHz, D_2O): δ 7.86 (td, *J* = 7.0, 1.6 Hz, 3H), 7.70 – 7.59 (m, 12H), 7.21 (d, *J* = 7.9 Hz, 2H), 6.98 (dd, *J* = 8.2, 2.5 Hz, 2H), 4.75 (d, *J* = 14.8 Hz, 2H), 4.57 (s, 2H) ppm. $^{13}C\{^{1}H\}$ NMR (125 MHz, D_2O): δ 140.7 (d, *J* = 4.0 Hz, C), 135.1 (d, *J* = 3.1 Hz, CH), 134.1 (d, *J* = 9.7 Hz, CH), 131.1 (d, *J* = 5.4 Hz, CH), 129.8 (d, *J* = 12.7 Hz, CH), 127.8 (d, *J* = 3.4 Hz, CH), 126.5 (d, *J=8.5* Hz C), 117.2 (d, *J* = 86.6 Hz, C), 63.3 (s, CH_2), 29.4 (d, *J* = 48.6 Hz, CH_2) ppm. $^{31}P\{^{1}H\}$ NMR (160 MHz, D_2O): δ 22.26 ppm. HRMS-(ESI) (*m/z*): calcd for [**P1**-Br]$^{+}$ $C_{26}H_{23}BrP^{+}$ 445.0715 found 445.0706.

[1] Yi, S.; Kaifer, A. E. Determination of the Purity of Cucurbit[n]uril (n = 7, 8) Host Samples. *J. Org. Chem.* **2011**, *76*, 10275.

SP2: Synthesis of semiaxle 1_a·2Br

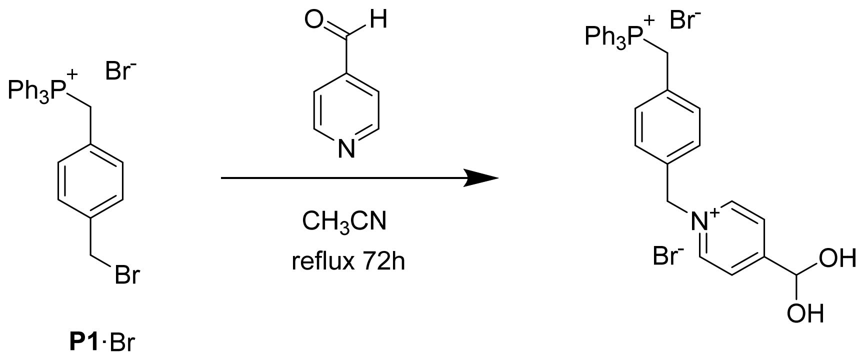

To a solution of **P1**·Br (0.60 g, 1.12 mmol) in 60 mL of CH_3CN 4-pyridilcarboxaldehide (428 mg, 3.40 mmol) was added. The mixture was heated under reflux using a heating mantle for 72 hours. Then, the solvent was removed in vacuum to leave an orange oil residue, which was subjected to flash chromatography (SiO_2, solvent: CH_2Cl_2/MeOH 8:2). The semiaxle containing fractions were combined and evaporated to yield $\mathbf{1_a}$·2Br as a pink oil (184 mg, 25%).

^{1}H NMR (500 MHz, D_2O): δ 8.90 (d, *J* = 6.7 Hz, 2H), 8.19 (d, *J* = 6.5 Hz, 2H), 7.92 – 7.84 (m, 3H), 7.73 – 7.61 (m, 12H), 7.30 (d, *J* = 7.9 Hz, 2H), 7.09 (dd, *J* = 8.4, 2.5 Hz, 2H), 6.25 (s, 1H), 5.79 (s, 2H), 4.83 (d, *J* = 15.0 Hz, 2H) ppm. ^{13}C{^{1}H} NMR (125 MHz, D_2O): δ 160.4 (C), 144.6 (CH), 135.2 (CH), 134.0 (d, *J* = 9.9 Hz, CH), 133.2 (d, *J* = 4.0 Hz, C), 131.9 (d, *J* = 5.4 Hz, CH), 129.9 (d, *J* = 12.8 Hz, CH), 129.9 (d, *J* = 12.8 Hz, C), 129.4 (d, *J* = 3.3 Hz, CH), 125.6 (CH), 117.0 (d, *J* = 86.7 Hz, C), 87.5 (CH), 63.6 (CH_2), 29.3 (d, *J* = 49.0 Hz, CH_2) ppm.^{31}P{^{1}H} NMR (160 MHz, D_2O): δ 22.71 ppm. HRMS-(ESI) (*m/z*): calcd. for [**1a**-Br-OH_2+MeOH]$^+$ $C_{32}H_{30}BrNO_2P^+$ 584.1349 found 584.1339.

SP3: Synthesis of semiaxle 1_b·2Br

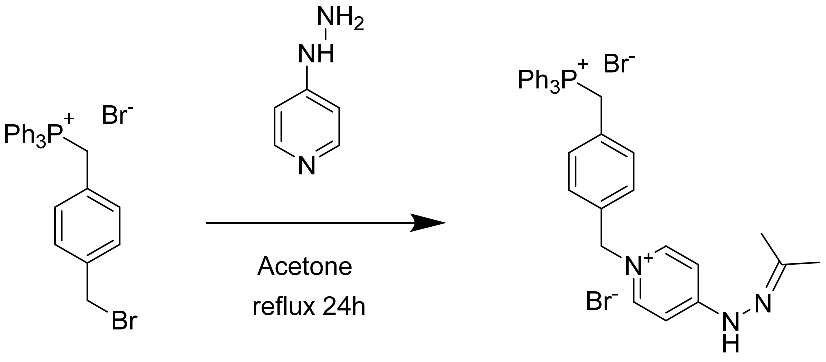

P1·Br

1b·2Br

4-hydrazineylpyridine (400 mg, 2.74 mmol) was suspended into 40 mL acetone, heated using a heating mantle, and stirred for 1 hour. After solubilization of the compound, **P1**·Br (300 mg, 0.54 mmol) was added, and the mixture was refluxed using a heating mantle for 18 hours. The white precipitate formed was filtered and washed with hot acetone and dried under vacuum to afford a white solid $\mathbf{1_b}$·2Br (245 mg, 67%).

^{1}H NMR (500 MHz, D_2O): δ 8.17 – 8.05 (m, 2H), 7.85 (ddd, *J* = 6.9, 5.3, 2.6 Hz, 3H), 7.69 – 7.58 (m, 12H), 7.40 – 7.32 (m, 2H), 7.19 (d, *J* = 8.3 Hz, 2H), 7.02 (dd, *J* = 8.3, 2.6 Hz, 2H), 5.32 (d, *J* = 1.4 Hz, 2H), 4.77 (s, 2H), 2.13 (s, 4H), 2.06 (s, 3H) ppm.^{13}C{^{1}H} NMR (125 MHz, D_2O): δ 163.1 (C), 154.3 (C), 142.4 (CH), 135.1 (d, *J* = 3.1 Hz, CH), 134.9 (d, *J* = 4.1 Hz, C), 134.0 (d, *J* = 9.9 Hz, CH), 131.7 (d, *J* = 5.4 Hz, CH), 129.8 (d, *J* = 12.7 Hz, CH), 128.6 (d, *J* = 3.3 Hz, CH), 128.5 (d, *J* = 8.5 Hz, C), 117.1 (d, *J* = 86.7 Hz, C), 107.0 (CH), 60.6 (CH_2), 29.3 (d, *J* = 49.0 Hz, CH_2), 24.3 (CH_3), 17.3 (CH_3).^{31}P{^{1}H} NMR (162 MHz, D_2O) δ 22.73 (s) ppm. HRMS-(ESI) (*m/z*): calcd for [**1b**-2Br]$^{2+}$ $C_{34}H_{34}N_3P^{2+}$ 257.6240 found 257.6238.

SP4: Synthesis of axle 2·4Cl

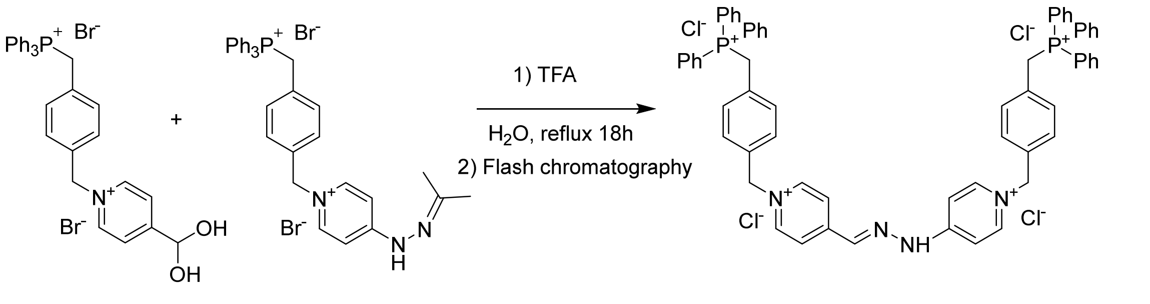

1$_a$·2Br **1**$_b$·2Br **2**·4Cl

A mixture of semiaxle **1**$_a$·2Br (15.5 mg, 0.024 mmol) and semiaxle **1**$_b$·2Br (16.0 mg, 0.024 mmol) was dissolved in water (4.8 mL) and trifluoroacetic acid (3.1 μL, 0.048 mmol) was added to assure the acidic conditions. The yellow solution was heated under reflux using a heating mantle for 18 hours. Then, the solvent was removed in vacuum to leave an orange oil residue, which was subjected to flash chromatography (SiO_2, solvent: CH_3CN/NaCl (0.6 M)/ MeOH 4:1:1). The axle containing fractions were combined and evaporated to yield **2**·4Cl as a hygroscopic yellow solid (18 mg, 69%). ^1H NMR (500 MHz, D_2O): δ 8.87 (d, J = 6.5 Hz, 2H), 8.41 (d, J = 6.9 Hz, 2H), 8.37 (d, J = 6.7 Hz, 2H), 8.33 (s, 1H), 7.83 (ddt, J = 7.6, 5.1, 2.1 Hz, 7H), 7.61 (ddt, J = 12.0, 6.5, 2.5 Hz, 28H), 7.31 (d, J = 7.9 Hz, 2H), 7.23 (d, J = 7.9 Hz, 2H), 7.07 (dd, J = 8.1, 2.4 Hz, 2H), 7.05 – 7.01 (m, 2H), 5.75 (s, 2H), 5.47 (s, 2H) ppm.$^{13}C\{^1H\}$ NMR (125 MHz, D_2O): δ 154.4 (C), 149.9 (C), 144.5 (CH), 143.9 (CH), 141.4 (CH), 135.2 (d, J = 3.0 Hz, CH), 134.5 (d, J = 3.9 Hz, C), 134.0 (d, J = 9.8 Hz, CH), 133.5 (d, J = 4.0 Hz, C), 132.0 (d, J = 5.5 Hz, CH), 131.8 (d, J = 5.4 Hz, CH), 129.9 (d, J = 12.6 Hz, CH), 129.4 (d, J = 3.3 Hz, CH), 129.3 (d, J = 8.6 Hz, C), 128.9 (d, J = 3.2 Hz, CH), 128.8 (d, J = 8.3 Hz, C), 125.1 (CH), 117.0 (d, J = 86.5 Hz, C), 109.5 (CH), 63.5 (CH_2), 61.4 (CH_2), 29.4 (d, J = 48.7 Hz, CH_2).$^{31}P\{^1H\}$ NMR (160 MHz, D_2O): δ 22.68 (s) ppm. HRMS-(ESI) (*m/z*): calcd for [**2**-H-3Cl]$^{3+}$ $C_{63}H_{55}N_4P_2^{3+}$ 309.7962 found 309.7954.

SP5: Synthesis of pseudorotaxane 1_a·2Br⊂CB[7]

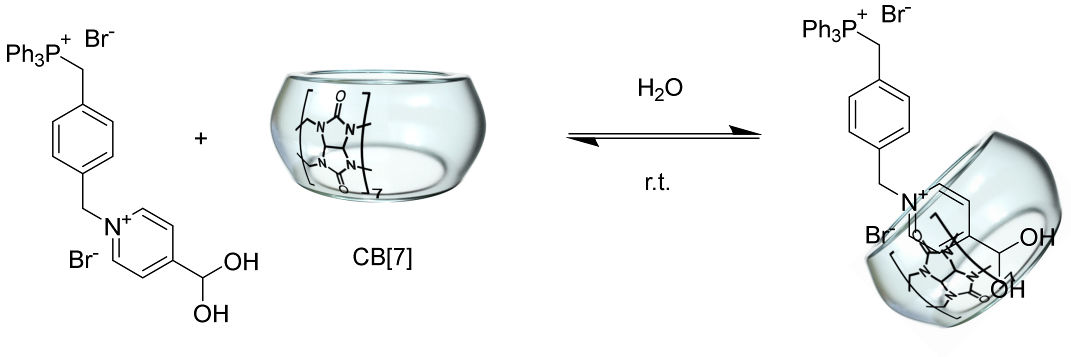

1$_a$·2Br **1**$_a$·2Br@CB[7]

The pseudorotaxane structure **1**$_a$·2Br⊂CB[7] was instantaneously formed after mixing appropriate amounts equimolar amounts of both components. Firstly, 2.0 mL of a 1.0 mM stock solution of **1**$_a$·2Br (1.3 mg, 0.002 mmol) were prepared in D_2O. Then, CB[7] (1.2 mg, 0.001 mmol) was weighted and dissolved using 1.0 mL of the previous 1.0 mM stock solution of **1**$_a$·2Br to yield the inclusion complex **1**$_a$·2Br⊂CB[7] as a pink oil (1.8 mg, 100%). ^{1}H NMR (400 MHz, D_2O): δ 8.27 (d, *J* = 6.3 Hz, 2H), 7.92 – 7.85 (m, 3H), 7.78 – 7.69 (m, 12H), 7.54 (d, *J* = 6.1 Hz, 2H), 7.24 (d, *J* = 7.8 Hz, 2H), 7.03 (d, *J* = 7.8 Hz, 2H), 5.77 (d, *J* = 15.4 Hz, 14H), 5.68 (s, 1H), 5.54 (s, 14H), 5.49 (s, 2H), 4.98 – 4.82 (m, overlapped, 2H), 4.24 (d, *J* = 15.4 Hz, 14H) ppm. ^{13}C NMR (125 MHz, D_2O): δ 161.1 (C), 156.2 (C), 144.2 (CH), 134.9 (d, *J* = 3.0 Hz, CH), 134.2 (d, *J* = 9.9 Hz, CH), 132.5 (C), 132.2 (d, *J* = 5.4 Hz, CH), 129.9 (d, *J* = 12.6 Hz, CH), 128.7 (CH), 124.4 (CH), 117.5 (d, *J* = 86.6 Hz, C), 87.3 (CH), 71.2 (CH), 63.8 (CH_2), 52.5 (CH_2), 29.3 (d, *J* = 48.9 Hz, CH_2).^{31}P{^{1}H} NMR (160 MHz, D_2O) δ 21.73 ppm. HRMS-(ESI) (*m/z*): calcd. for [**1a**⊂CB[7]-2Br-OH_2+MeOH]$^{2+}$ $C_{75}H_{74}N_{29}O_{16}P^{2+}$: calcd. 833.7797 exp. 833.7803.

SP6: Synthesis of pseudorotaxane 1_b·2Br ⊂CB[7]

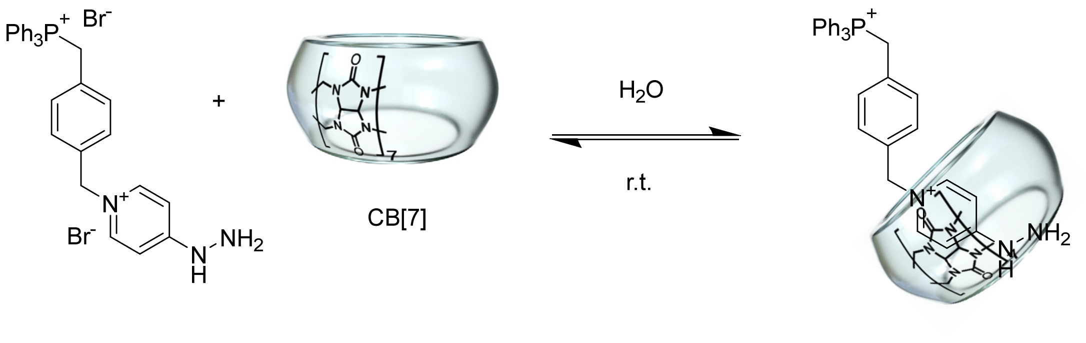

$\mathbf{1_b}$·2Br

$\mathbf{1_b}$·2Br@CB[7]

The pseudorotaxane structure $\mathbf{1_b}$ 2Br⊂CB[7] was instantaneously formed after mixing appropriate amounts equimolar amounts of both components. Firstly, 2.0 mL of a 1.0 mM stock solution of $\mathbf{1_b}^{\mathbf{2+}}$ (1.3 mg, 0.002 mmol) were prepared in D_2O. Then, CB[7] (1.2 mg, 0.001 mmol) was weighted and dissolved using 1.0 mL of the previous 1.0 mM stock solution of $\mathbf{1_b}$·2Br stock solution to yield the inclusion complex $\mathbf{1_a}$·2Br⊂CB[7] as a yellow oil (1.8 mg, 100%). ^{1}H NMR (400 MHz, D_2O): δ 7.88 (dd, *J* = 8.2, 5.6 Hz, 3H), 7.81 – 7.68 (m, 12H), 7.31 – 7.24 (m, 2H), 7.11 (d, *J* = 8.0 Hz, 2H), 5.80 (d, *J* = 15.4 Hz, 14H), 5.55 (s, 14H), 5.11 (s, 2H), 4.96 – 4.84 (m, 2H), 4.25 (d, *J* = 15.1 Hz, 14H) ppm.^{13}C NMR (125 MHz, D_2O): δ 158.7 (C), 156.4 (C), 134.8 (CH), 134.3 (d, *J* = 9.8 Hz, CH), 132.1 (CH), 129.8 (d, *J* = 12.7 Hz, CH), 128.5 (CH), 117.6 (d, *J* = 86.4 Hz, C), 71.2 (CH), 60.3 (CH_2), 52.5 (CH_2), 29.5 (d, *J* = 47.8 Hz, CH_2).^{31}P{^{1}H} NMR (160 MHz, D_2O): δ 20.81 ppm. HRMS-(ESI) (*m/z*): calcd. for [**1b**⊂CB[7]-2Br]$^{2+}$ $C_{73}H_{74}N_{31}O_{14}P^{2+}$: calcd. 819.7879 exp. 819.7869.

SP7: Synthesis of rotaxane 2·4PF_6⊂CB[7]:

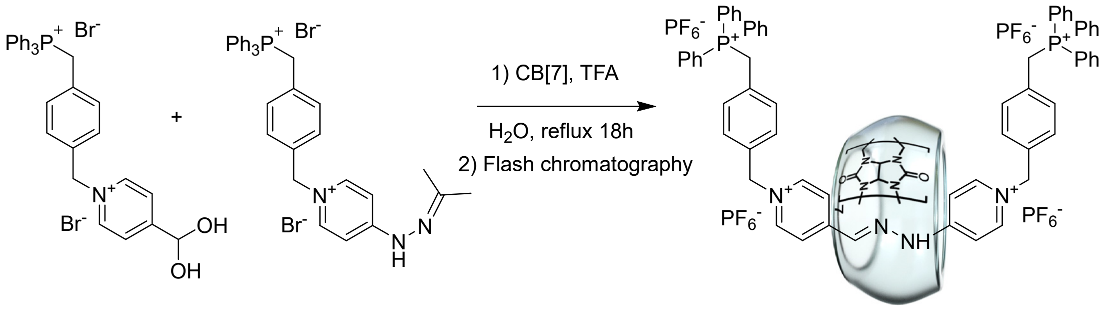

1a·2Br **1b**.2Br **2**·4PF6 @CB[7] **2**·4Cl@CB[7]

A mixture of semiaxle **1a**·2Br (9.4 mg, 0.014 mmol) and semiaxle **1b**·2Br (9.7 mg, 0.014 mmol), cucurbit[7]uril (45 mg, 0.026 mmol) was dissolved in water (6.0 mL) and trifluoroacetic acid (1.8 μL, 0.028 mmol) was added to assure the acidic conditions. The yellow solution was heated under reflux using a heating mantle for 18 hours. Then, the solvent was removed in vacuum to leave a yellow crude, which was subjected to flash chromatography (SiO_2) with two different eluent phases as solvents: (a) (CH_3CN/NaCl (0.6 M)/ MeOH 4:1:1) for eluting the impurities and (b) (CH_3CN/KPF_6 (0.6 M)/ MeOH 4:1:1) for eluting the rotaxane. The rotaxane containing fractions were combined and evaporated to yield **2**·4PF_6⊂CB[7] as a hygroscopic yellow oil (20 mg, 55%).^{1}H NMR (400 MHz, CD_3CN): δ 10.62 (s, 1H), 8.25 (d, *J* = 7.0 Hz, 2H), 8.15 (d, *J* = 6.2 Hz, 2H), 7.91 – 7.82 (m, 8H), 7.79 – 7.66 (m, 10H), 7.66 – 7.53 (m, 12H), 7.44 (d, *J* = 6.7 Hz, 2H), 7.27 (m, 4H), 7.22 (d, *J* = 7.6 Hz, 2H), 7.11 (d, *J* = 7.9 Hz, 2H), 7.02 (d, *J* = 7.7 Hz, 2H), 5.69 (dd, *J* = 23.3, 15.0 Hz, 14H), 5.69 (s, overlapped, 2H), 5.40 (s, 2H), 5.26 (s, 14H), 4.69 (t, *J* = 14.3 Hz, 4H), 4.02 (dd, *J* = 15.0, 4.1 Hz, 14H) ppm.^{13}C{^{1}H} NMR (125 MHz, CD_3CN): δ 156.2 (C), 156.0 (C), 155.2 (C), 150.0 (C), 145.9 (CH), 144.6 (CH), 139.3 (CH), 136.3 (d, *J* = 3.2 Hz, CH), 136.1 (d, *J* = 3.2 Hz, CH), 135.2 (d, *J* = 9.5 Hz, CH), 135.1 (d, *J* = 9.6 Hz, CH), 132.9 (d, *J* = 5.4 Hz, CH), 132.7 (d, *J* = 5.5 Hz, CH), 131.3 (d, *J* = 9.1 Hz, CH), 131.2 (d, overlapped, C), 131.2 (d, *J* = 9.3 Hz, CH), 131.1 (d, *J* = 2.5 Hz, C), 130.2 (d, *J* = 3.2 Hz, CH), 130.0 (d, *J* = 3.3 Hz, CH), 129.3 (d, *J* = 8.4 Hz, C), 129.1 (d, *J* = 8.1 Hz, C), 123.8 (CH), 118.5 (d, C, overlapped), 118.3 (d, *J* = 86.2 Hz, C),112.8 (CH), 71.6 (CH), 64.6 (CH_2), 62.2 (CH_2), 53.3 (CH_2), 53.2 (CH_2), 30.3 (d, *J* = 48.7 Hz, CH_2), 30.1 (d, *J* = 48.6 Hz, CH_2) ppm.^{31}P{^{1}H} NMR (160 MHz, CD_3CN): δ 22.81 (s), 22.54 (s) ppm. HRMS-(ESI) (*m/z*): calcd for [**2**⊂CB[7]-4PF_6]$^{4+}$ $C_{105}H_{98}N_{32}O_{14}P_2^{4+}$ 523.4357 found 523.4347. [**2**⊂CB[7]-H-3PF_6]$^{3+}$ $C_{105}H_{97}N_{32}O_{14}P_2^{3+}$697.5785 found 697.5774.[**2**⊂CB[7]-3PF_6]$^{3+}$ $C_{105}H_{98}N_{32}F_6O_{14}P_3^{3+}$

746.2358 found 746.2348.[**2**⊂CB[7]-H-PF_6]$^{2+}$ $C_{105}H_{97}F_6N_{32}F_{12}O_{14}P_3^{2+}$ 1118.8501 found 1118.8494. [**2**⊂CB[7]-2PF_6]$^{2+}$ $C_{105}H_{98}N_{32}F_{12}O_{14}P_4^{2+}$ 1191.8361 found 1191.8352.

Rotaxane **2**·4PF_6⊂CB[7] (10 mg, 0.004 mmol) was dissolved in 3 mL of a mixture of CH_3CN/ KCl_{aq} (1M). The mixture was stirred for 72 hours and the solvent was removed in order to obtain the rotaxane soluble in pure water as a chloride salt **2**·4Cl⊂CB[7]. ^1H NMR (500 MHz, D_2O) δ 8.95 (d, *J* = 6.6 Hz, 2H), 8.84 (d, *J* = 6.4 Hz, 2H), 8.51 – 8.42 (m, 6H), 8.34 – 8.11 (m, 24H), 8.02 (d, *J* = 8.0 Hz, 2H), 7.92 (t, *J* = 7.7 Hz, 4H), 7.82 (d, *J* = 6.7 Hz, 2H), 7.70 (dd, *J* = 8.3, 2.5 Hz, 2H), 7.67 (s, 1H), 7.61 (dd, *J* = 8.3, 2.5 Hz, 2H), 6.31 (s, 2H), 6.21 (dd, *J* = 23.7, 15.2 Hz, 14H), 6.06 (s, 2H), 5.95 (s, 14H), 5.34 (dd, *J* = 22.9, 14.7 Hz, 4H), 4.69 (dd, *J* = 15.3, 5.5 Hz, 14H).$^{13}C\{^1H\}$ NMR (125 MHz, D_2O): δ 155.3 (C), 155.2 (C), 153.6 (C), 148.4 (C), 144.4 (CH), 136.0 (C), 135.0 (CH), 134.9 (d, *J* = 3.0 Hz), 134.8 (d, *J* = 3.1 Hz), 134.40 (d, *J* = 3.8 Hz), 133.7 (d, *J* = 9.7 Hz), 133.6 (d, *J* = 10.0 Hz), 132.7 (d, C, *J* = 3.7 Hz), 131.5 (d, *J* = 5.4 Hz), 131.3 (d, *J* = 5.4 Hz), 129.9 (d, *J* = 3.9 Hz), 129.8 (d, *J* = 3.9 Hz), 128.8 (d, *J* = 3.2 Hz), 127.9 (dd, C, *J* = 8.4, 4.7 Hz), 121.8 (CH), 117.3 (d, *J* = 12.5 Hz), 116.6 (d, *J* = 12.7 Hz), 110.9, 70.4 (CH), 63.1 (CH_2), 60.9 (CH_2), 52.1 (CH_2), 28.8 (dd, CH_2 *J* = 48.8, 30.9 Hz). $^{31}P\{^1H\}$ NMR (160 MHz, D_2O): δ 22.80 (s), 22.53 (s) ppm. HRMS-(ESI) (*m/z*): calcd for [**2**⊂CB[7]-4Cl]$^{4+}$ $C_{105}H_{98}N_{32}O_{14}P_2^{4+}$ 523.4357 found 523.4349.

SP8: Synthesis of pseudorotaxane 1_a·2Br⊂CB[8]:

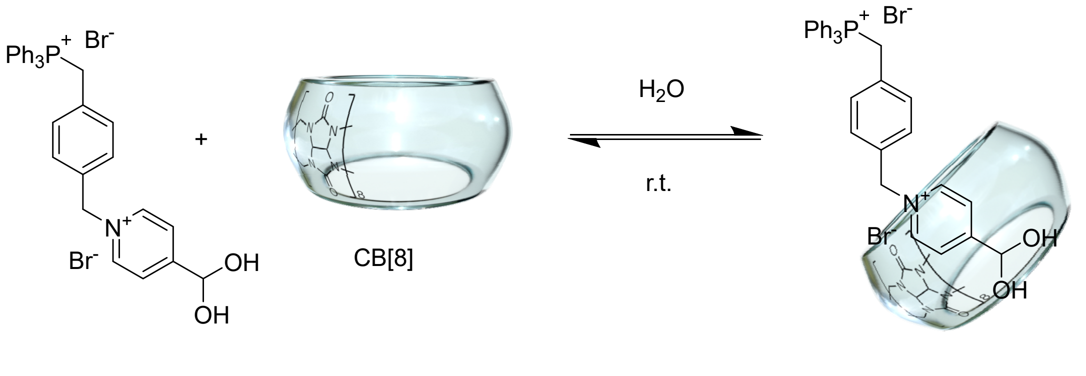

1$_a$·2Br **1**$_a$·2Br@CB[8]

A solution of **1**$_a$·2Br (2.6 mg, 0.004 mmol) in 2 mL of D_2O was prepared. Then, cucurbit[8]uril was added in excess and the mixture was sonicated and heated using an oil bath at 60 °C for 15 min. Finally, the sample was filtered off to remove the excess of CB[8] to yield the inclusion complex **1**$_a$·2Br⊂CB[8] as a pink oil (4 mg, 77%). ^{1}H NMR (500 MHz, D_2O): δ 8.34 (d, *J* = 6.4 Hz, 2H), 8.26 (d, *J* = 5.4 Hz, 2H), 7.97 – 7.87 (m, 4H), 7.77 (q, *J* = 3.4 Hz, 7H), 6.74 (s, 1H), 6.65 (dd, *J* = 12.8, 7.8 Hz, 2H), 6.32 (m, 4H), 6.27 (td, *J* = 7.8, 2.7 Hz, 2H), 5.71 (dd, *J* = 124.2, 15.2 Hz, 16H), 5.45 – 5.41 (m, 16H), 5.39 (s, 2H), 5.22 (s, 2H), 4.12 (dd, *J* = 60.8, 15.3 Hz, 16H) ppm. ^{13}C{^{1}H} NMR (125 MHz, D_2O): δ 161.6 (C), 156.8 (C), 156.1 (C), 143.3 (CH), 134.4 (d, *J* = 9.7 Hz, CH), 134.1, 133.0 (d, *J* = 9.9 Hz, CH), 132.0 (d, *J* = 12.3 Hz, CH), 131.0 (d, *J* = 5.8 Hz, CH), 130.5 (d, *J* = 12.8 Hz, CH), 127.4 – 127.0 (m), 126.3 (CH), 119.6 (d, *J* = 84.4 Hz, C), 87.6 (CH), 71.8 (CH), 63.9 (CH2), 53.4 (d, *J* = 34.5 Hz) ppm. ^{31}P{^{1}H} NMR (160 MHz, D_2O) δ 24.13, 24.04 ppm. HRMS-(ESI) (*m/z*): calcd for [**1**$_a$⊂CB[8]-2Br-OH_2+MeOH]$^{2+}$ $C_{80}H_{78}N_{33}O_{18}P^{2+}$ 909.7965, found 909.7973.

SP9: Synthesis of pseudorotaxane 1_b·2Br⊂CB[8]:

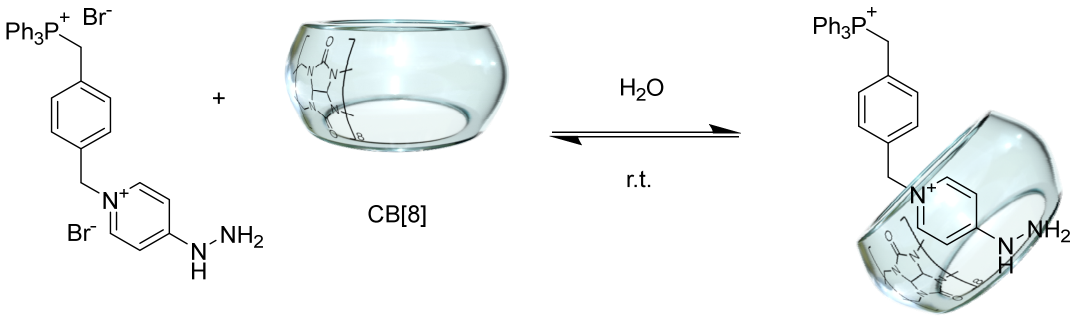

1$_b$·2Br **1**$_b$·2Br@CB[8]

A solution of **1**$_b$·2Br (2.7 mg, 0.004 mmol) in 2 mL of D_2O was prepared. Then, cucurbit[8]uril was added in excess and the mixture was sonicated and heated using an oil bath at 60 °C for 15 min. Finally, the sample was filtered off to remove the excess of CB[8] to yield the inclusion complex **1**$_b$·2Br⊂CB[8] as a white oil (3.5 mg, 66%). ^{1}H NMR (500 MHz, D_2O): δ 7.90 (dt, *J* = 13.0, 4.2 Hz, 4H), 7.72 (q, *J* = 3.3 Hz, 7H), 7.00 (s, 2H), 6.65 (dd, *J* = 12.8, 7.7 Hz, 2H), 6.33 – 6.25 (m, 5H), 6.18 (d, *J* = 7.9 Hz, 2H), 6.08 (t, *J* = 7.6 Hz, 1H), 5.66 (dd, *J* = 110.9, 15.2 Hz, 16H), 5.38 (s, 16H), 4.67 (s, 2H), 4.43 (d, *J* = 12.8 Hz, 2H), 4.07 (dd, *J* = 53.5, 15.3 Hz, 16H), 2.11 (m, 3H) ppm. ^{13}C{^{1}H} NMR (125 MHz, D_2O): δ 157.5 (C), 156.8 (C), 156.6 (C), 156.1 (C), 134.2 (d, *J* = 7.3 Hz, CH), 134.0 (CH), 133.0 (d, *J* = 9.8 Hz, CH), 130.9 (d, *J* = 7.5 Hz, C), 130.8 (d, *J* = 5.9 Hz, CH), 130.4 (d, *J* = 12.9 Hz, CH), 127.2 (d, *J* = 13.2 Hz, CH), 126.9 (d, *J* = 3.3 Hz, CH), 119.9 (d, *J* = 84.7 Hz, C), 71.8 (CH), 60.8 (CH_2), 53.5 (C), 53.2 (C), 30.0 (CH_3), 28.5 (d, *J* = 46.3 Hz, CH_2) ppm.^{31}P{^{1}H} NMR (160 MHz, D_2O): δ 24.28 ppm. HRMS-(ESI) (*m/z*): calcd for [**1**$_b$⊂CB[8]-2Br]$^{2+}$ $C_{79}H_{78}N_{35}O_{16}P^{2+}$ 901.8046, found 901.8064

SP10: Synthesis of [3]pseudorotaxane 2·4Cl⊂(CB[8])$_2$:

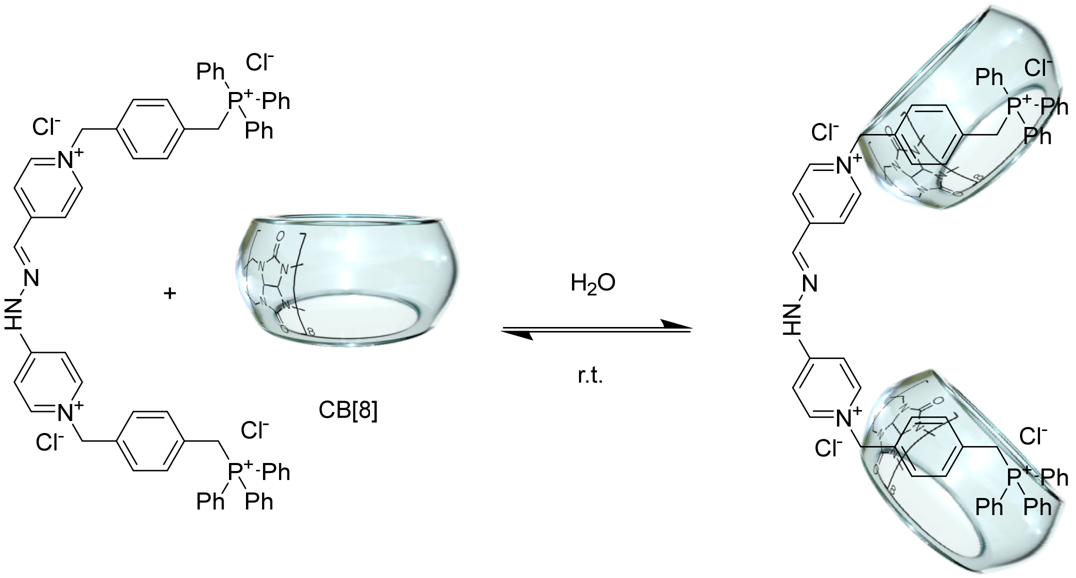

2·2Br **2**·4Cl@(CB[8])$_2$

A solution of **2**·4Cl (4.3 mg, 0.004 mmol) in 2 mL of D_2O was prepared. Then, cucurbit[8]uril was added in excess and the mixture was sonicated and heated using an oil bath at 60 °C for 15 min. Finally, the sample was filtered off to remove the excess of CB[8] to yield the inclusion complex **2**·4Cl⊂CB[8] as a pink oil (5.1 mg, 64%).^{1}H NMR (500 MHz, D_2O): δ 8.96 (s, 1H), 8.50 (d, *J* = 6.5 Hz, 2H), 8.20 (d, *J* = 6.5 Hz, 2H), 8.03 (m, 8H), 7.85 (m, 12H), 6.85 (m, 2H), 6.82 – 6.74 (m, 2H), 6.55 (m, 2H), 6.48 – 6.37 (m, 8H), 6.30 (d, *J* = 7.7 Hz, 2H), 6.25 (s, 1H), 5.88 (dd, *J* = 17.6, 15.3 Hz, 16H), 5.68 (dd, *J* = 15.2, 5.6 Hz, 16H), 5.49 (d, *J* = 1.6 Hz, 32H), 5.26 (s, 2H), 4.96 (d, *J* = 6.7 Hz, 2H), 4.59 (d, *J* = 4.9 Hz, 2H), 4.57 (d, *J* = 5.0 Hz, 2H), 4.33 – 4.18 (m, 16H), 4.14 (dd, *J* = 15.3, 2.0 Hz, 16H) ppm. ^{13}C{^{1}H} NMR (125 MHz, D_2O): δ 156.7 (C), 156.6 (C), 156.3 (C), 156.3 (C), 143.6 (CH), 142.9 (CH), 134.2 (CH), 133.1 (CH), 130.6 (CH), 127.9 (CH), 125.8 (CH), 71.9 (CH), 71.8 (CH), 53.6 (CH_2) 53.6 (CH_2), 53.4 (CH_2) ppm.^{31}P{^{1}H} NMR (160 MHz, D_2O): δ 24.45 (s), 24.29 (s) ppm. HRMS-(ESI) (*m/z*): calcd for [**2**⊂(CB[8])$_2$-4Cl]$^{4+}$ $C_{159}H_{152}N_{68}O_{32}P_2^{4+}$ 897.0461 found 897.0462.

NMR titrations of semiaxles 1_a·2Br and 1_b·2Br with CB[7]:

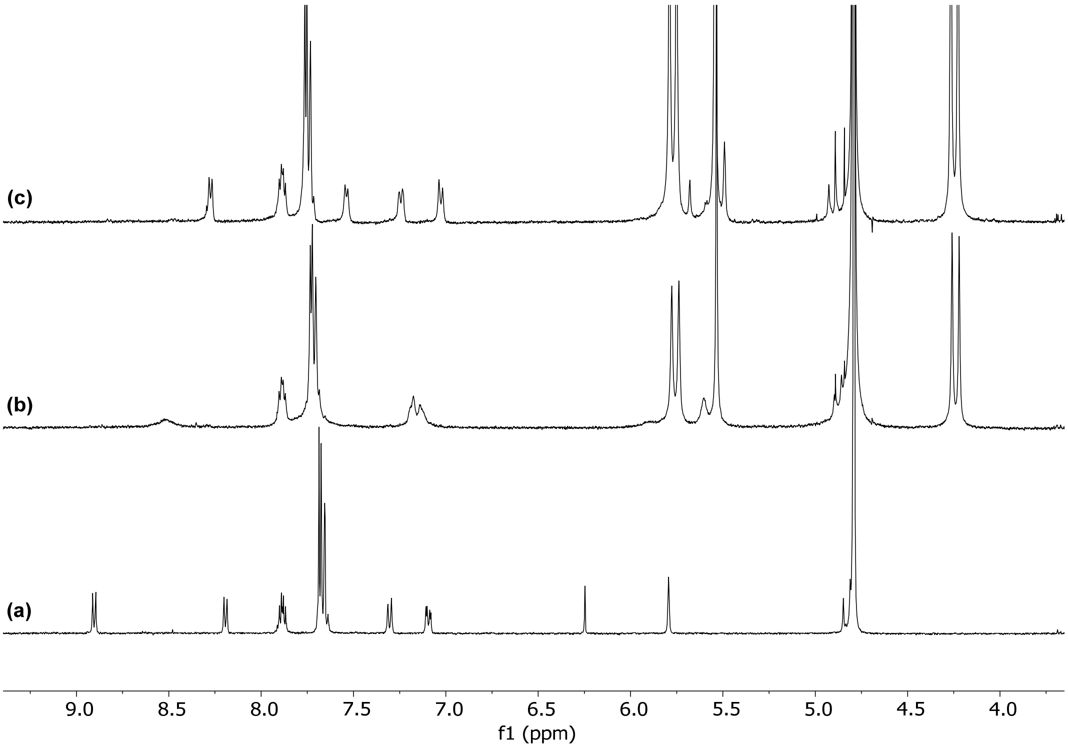

Figure S1 Partial ^{1}H NMR (400 MHz, D_2O): (a) 1 mM of semiaxle $\mathbf{1_a}$·2Br, (b) 1 mM of semiaxle $\mathbf{1_a}$·2Br + 0.5 eq of CB[7] and (c) 1 mM of semiaxle $\mathbf{1_a}$·2Br + 1.0 eq of CB[7].

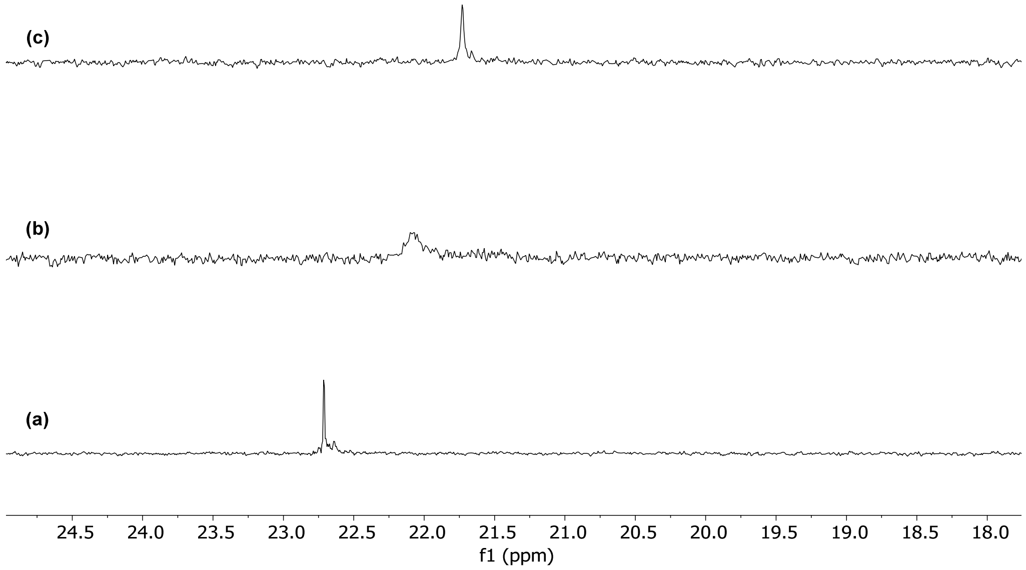

Figure S2 Partial $^{31}P\{^{1}H\}$ NMR (160 MHz, D_2O): (a) 1 mM of semiaxle $\mathbf{1_a}$·2Br, (b) 1 mM of semiaxle $\mathbf{1_a}$·2Br + 0.5 eq of CB[7] and (c) 1 mM of semiaxle $\mathbf{1_a}$·2Br + 1.0 eq of CB[7].

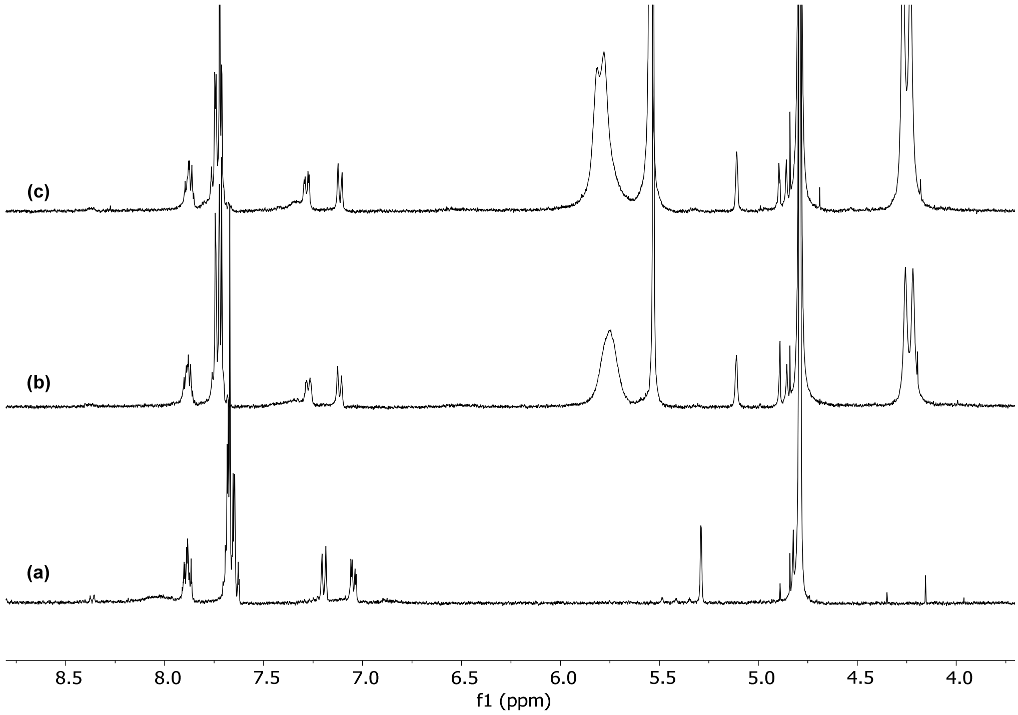

Figure S3 Partial ^{1}H NMR (400 MHz, D_2O): (a) 1 mM of semiaxle $\mathbf{1_b}$·2Br, (b) 1 mM of semiaxle $\mathbf{1_b}$·2Br + 0.5 eq of CB[7] and (c) 1 mM of semiaxle $\mathbf{1_b}$·2Br + 1.0 eq of CB[7].

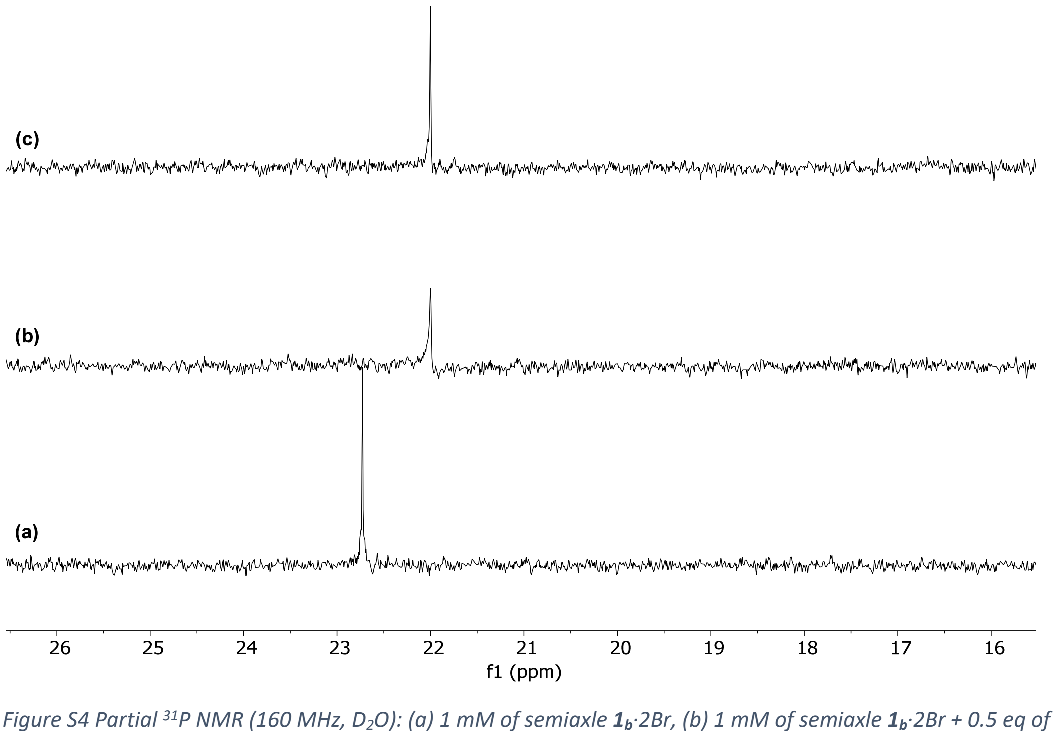

Figure S4 Partial ^{31}P NMR (160 MHz, D_2O): (a) 1 mM of semiaxle $\mathbf{1_b}$·2Br, (b) 1 mM of semiaxle $\mathbf{1_b}$·2Br + 0.5 eq of CB[7] and (c) 1 mM of semiaxle $\mathbf{1_b}$·2Br + 1.0 eq of CB[7].

DOSY of 2·4PF_6⊂CB[7]:

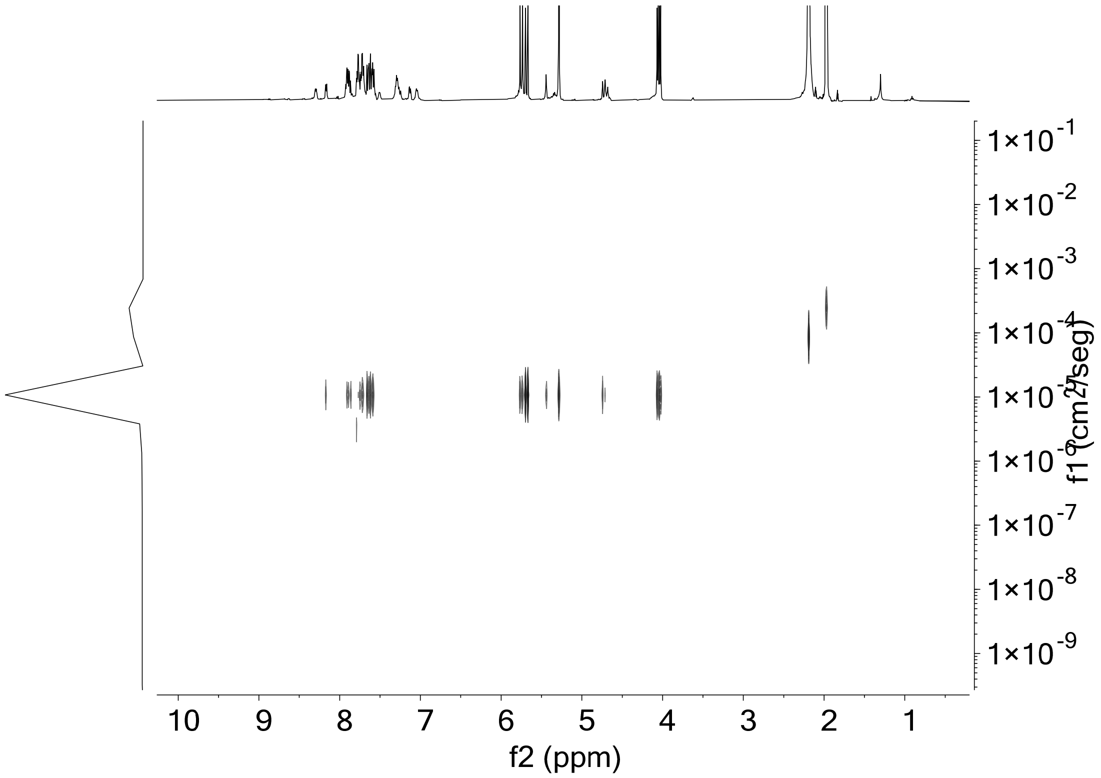

*Figure S5 DOSY NMR (500 MHz, CD_3CN) of **2**·4PF_6⊂CB[7].*

Calculation of Host-Guest Binding Constant by Competitive 1H NMR spectroscopy with ferrocenylmethyl trimethylammonium iodide (Fc·I):

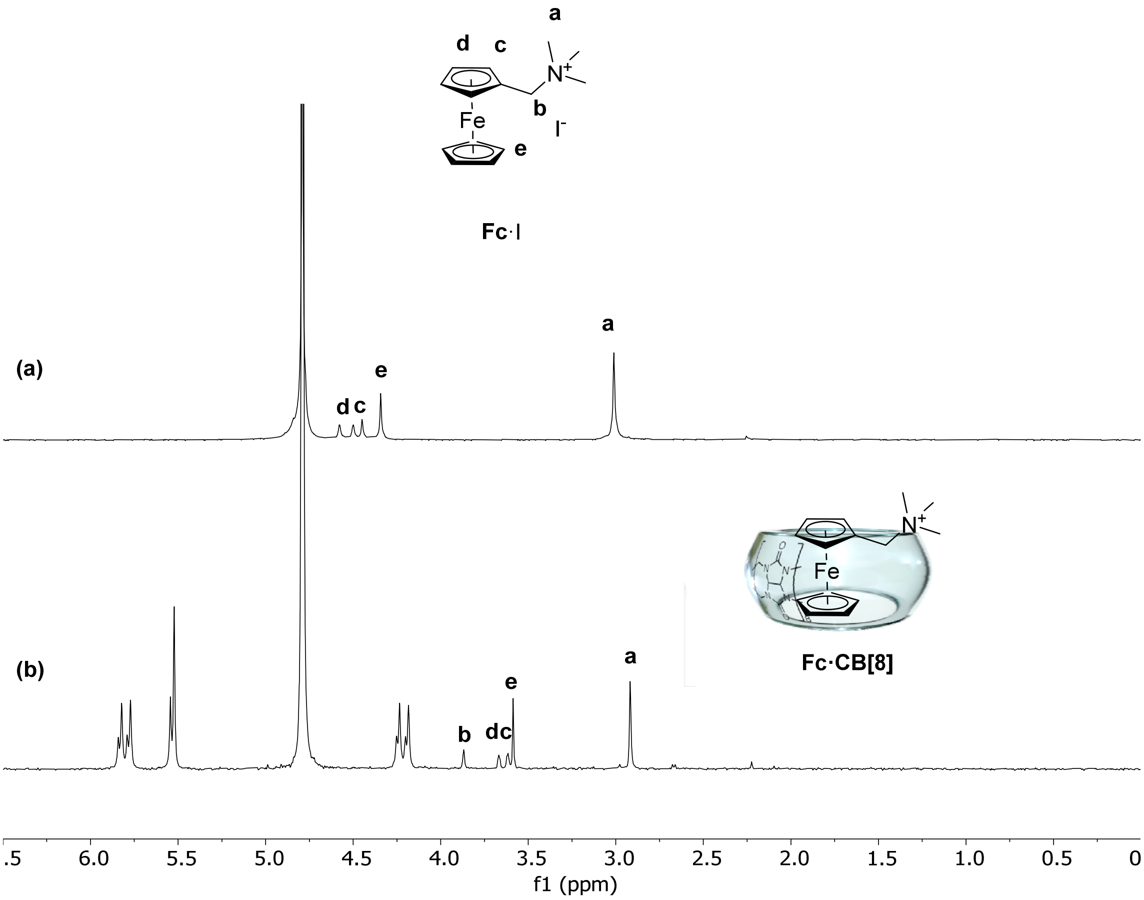

*Figure 6. Partial ^{1}H NMR spectrum (500 MHz, D_2O) of: (a) 1mM solution of **Fc+** and (b) 1mM solution of **Fc+**⊂CB[8] complex.*

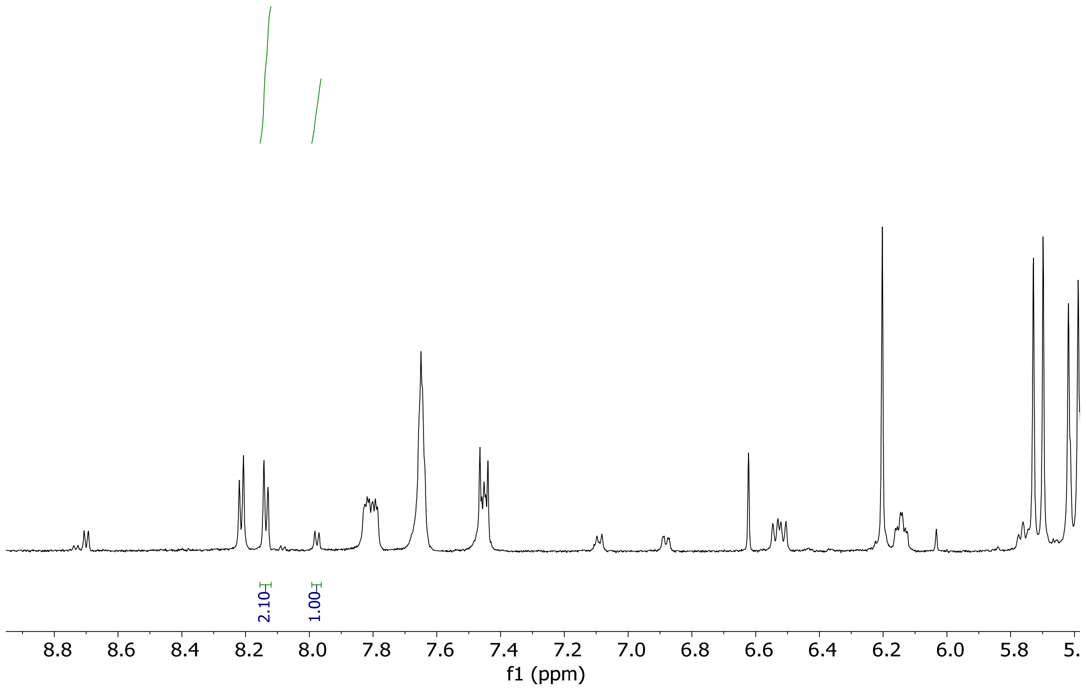

*Figure S7 Partial ^1H NMR spectrum (500 MHz, D_2O) of 500 μL of 1.00 mM solution of **1_a^{2+}**⊂CB[8] complex with 250 μL of 5.00 mM solution of **Fc^+**.*

$[1_a]_{total}$ = 1.00 mM·[0.50 mL/ (0.50 mL + 0.25 mL] = 0.66 mM.
$[Fc]_{total}$ = 5.00 mM [0.25 mL/(0.50 mL +0.25 mL)] = 1.66 mM.

$[1_a]_{free}$ = 0.66 mM [1.00/(1.00+2.10)] = 0.21 mM.
$[1_a]_{bound}$ = 0.66 mM [2.10/(1.00+2.10)] = 0.45 mM.

$[Fc]_{bound}$ = $[1a]_{free}$ = 0.21mM.
$[Fc]_{free}$ = $[Fc]_{total}$ - $[Fc]_{bound}$ =1.66 mM -0.21 mM= 1.45 mM.

$$K_{CB[8]\subset 1a} = \frac{[1_a]_{\mathrm{bound}} \cdot [Fc]_{free}}{[1_a]_{free} \cdot [Fc]_{bound}} \cdot K_{CB[8]\subset Fc} = \frac{0.45\ mM \cdot 1.45\ mM}{0.21\ mM \cdot 0.21\ mM} \cdot 10^{9.49}\ M^{-1} =$$

$$= 4.6 \cdot 10^{10}\ M^{-1}$$

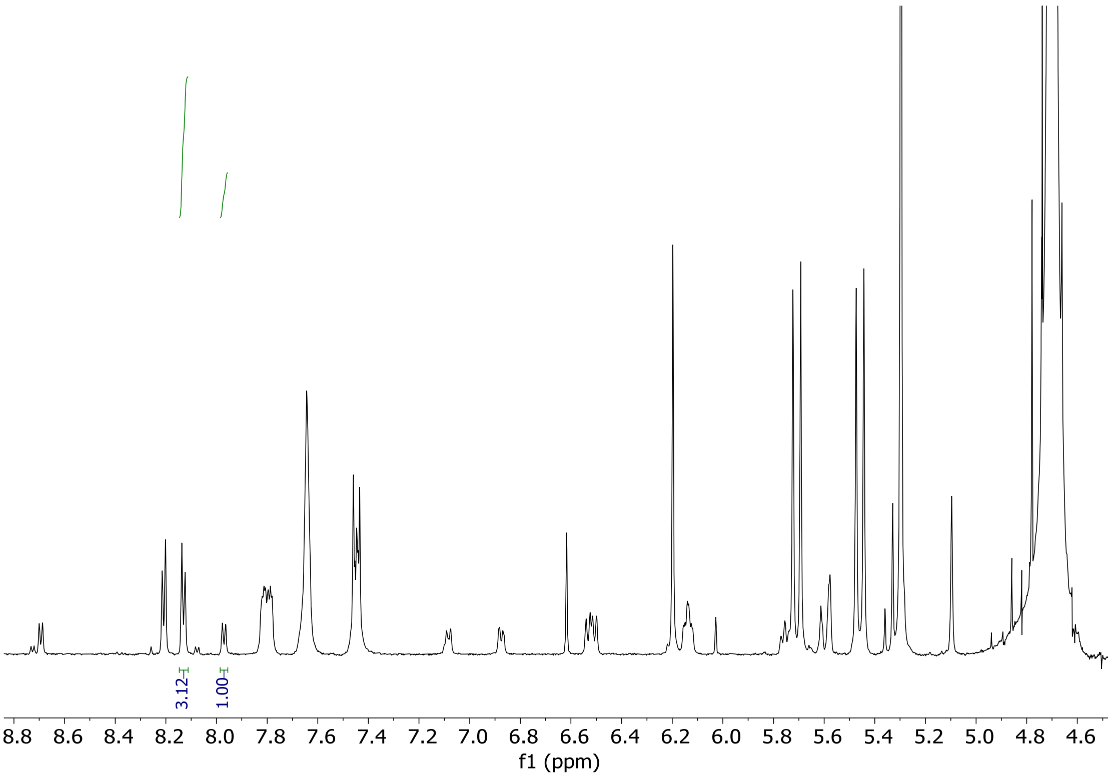

*Figure S8 Partial ^1H NMR spectrum (500 MHz, D_2O) of 500 μL of 1.00 mM solution of **Fc^+**⊂CB[8] complex with 200 μL of 2.00 mM solution of **1_a^{2+}**.*

$[1_a]_{total}$ = 2.00 mM·[0.20 mL/(0.50 mL +0.20 mL] = 0.57 mM.
$[Fc]_{total}$ = 1.00 mM [0.50 mL/(0.50 mL +0.20 mL)] = 0.71 mM.

$[1_a]_{free}$ = 0.66 mM [1.00/(1.00+3.12)] = 0.16 mM.
$[1_a]_{bound}$ = 0.66 mM [3.12/(1.00+3.12)] = 0.41 mM.

$[Fc]_{bound}$ = $[1a]_{free}$ = 0.16mM.
$[Fc]_{free}$ = $[Fc]_{total}$ - $[Fc]_{bound}$ =0.71 mM -0.16 mM= 0.55 mM.

$$K_{CB[8]\subset 1a} = \frac{[1_a]_{\text{bound}} \cdot [Fc]_{free}}{[1_a]_{free} \cdot [Fc]_{bound}} \cdot K_{CB[8]\subset Fc} = \frac{0.41\ mM \cdot 0.55\ mM}{0.16\ mM \cdot 0.16\ mM} \cdot 10^{9.49} M^{-1} =$$

$$= 2.7 \cdot 10^{10}\ M^{-1}$$

$$\boldsymbol{K_{CB[8]\subset 1a} = (3.6 \pm 0.7) \cdot 10^{10}\ M^{-1}}$$

Determination of the energy of the exchange barrier ($\Delta G^{\neq}$):

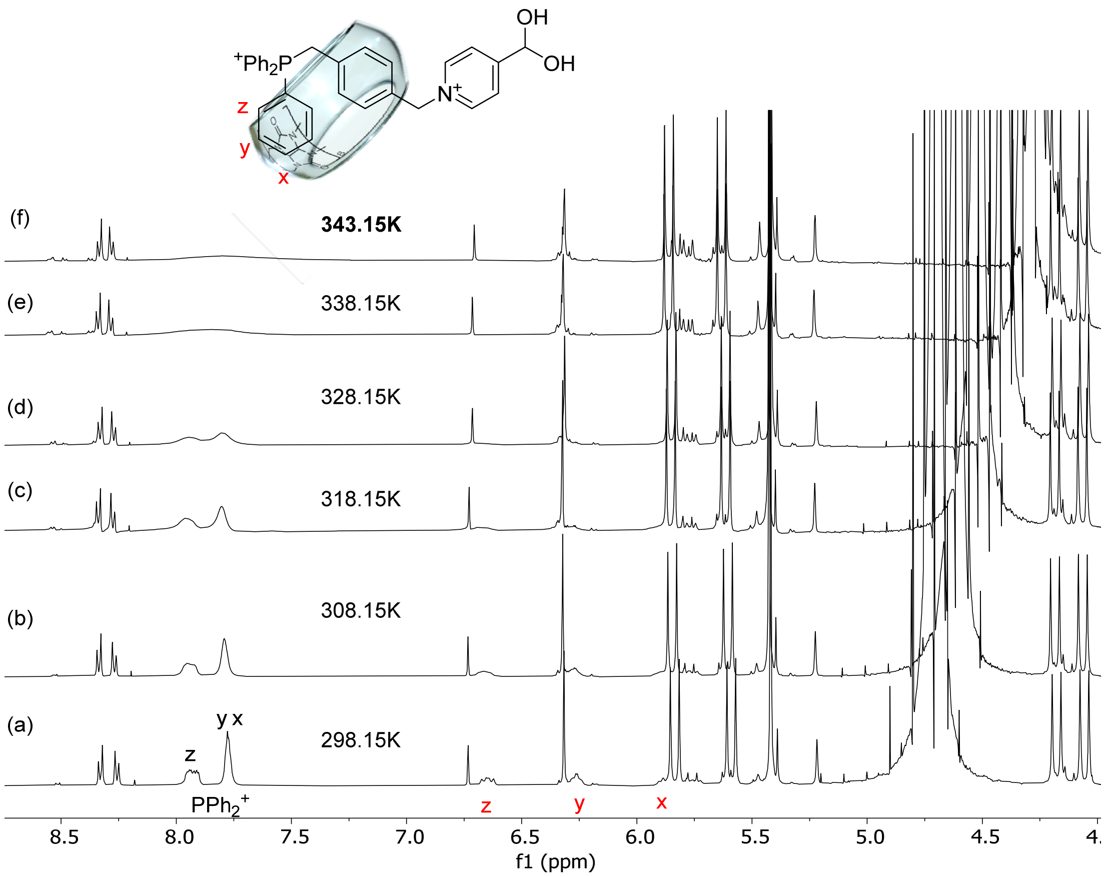

Figure S9 Partial ^1H NMR spectra (500 MHz, D_2O) of $\mathbf{1_a}^{2+}\subset$CB[8] at different temperatures.

$$\Delta G^{\neq} = 4.57 \cdot 10^{-3}\,\mathbf{Tc}\,(9.972 + \log \mathbf{Tc/\Delta\nu})\ (1)$$

Tc= 343.15 K

$\Delta\nu_z$ = 509.80 Hz	$\mathbf{\Delta G^{\neq}_z}$ = 15.36 kcal/mol
$\Delta\nu_y$ = 606.72 Hz	$\mathbf{\Delta G^{\neq}_y}$ = 15.24 kcal/mol
$\Delta\nu_x$ = 760.22 Hz	$\mathbf{\Delta G^{\neq}_x}$ = 15.09 kcal/mol

$\Delta G^{\neq}_{average}$ = 15.23 kcal/mol

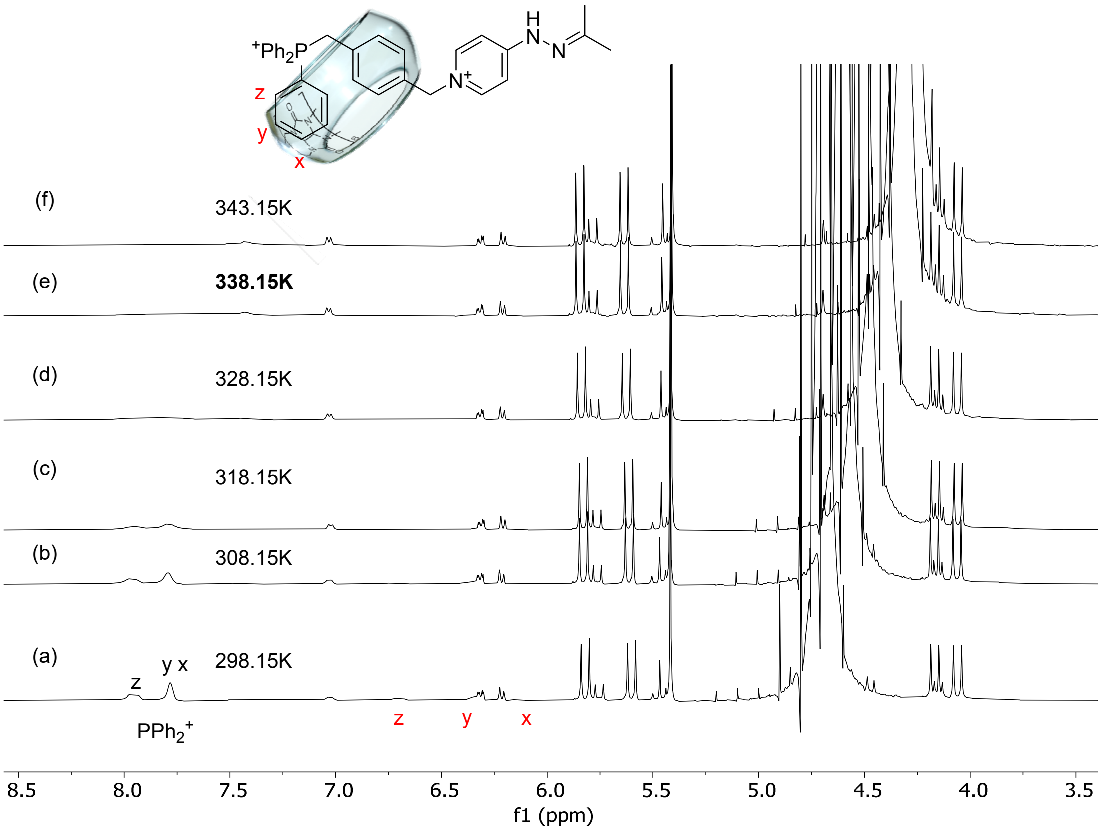

Figure S10 Partial ^{1}H NMR spectra (500 MHz, D_2O) of $\mathbf{1_b}^{2+}\subset$CB[8] at different temperatures.

Equation (1): $\Delta G^{\neq} = 4.57 \cdot 10^{-3}\,\mathbf{Tc}\,(9.972 + \log \mathbf{Tc}/\Delta\nu)$ (1)

Tc= 338.15 K

$\Delta\nu_z$ = 371.93 Hz $\quad$ $\mathbf{\Delta G^{\neq}_z}$ = 15.34 kcal/mol

$\Delta\nu_y$ = 430.37 Hz $\quad$ $\mathbf{\Delta G^{\neq}_y}$ = 15.24 kcal/mol

$\Delta\nu_x$ = 655.59 Hz $\quad$ $\mathbf{\Delta G^{\neq}_x}$ = 14.96 kcal/mol

$\Delta G^{\neq}_{average}$ = 15.01 kcal/mol

Computational details:

Semiempirical (pm6), and Density Functional Theory (DFT) calculations reported in this work were carried out using the G16 C.01 program.[2] Geometry optimizations at the DFT level of theory were performed using the BLYP-D3(BJ) functional: combination of the Becke exchange functional B and LYP correlation functional,[3,4] dispersion corrected by using the D3 version of Grimme's empirical dispersion scheme with Becke-Johnson damping (D3(BJ)).[5] The BLYP-D3(BJ) functional was used in combination with the valence double-zeta size polarization basis set def2-SVP for the energy minimizations this work, as it has been recently proven to afford reliable optimized geometries for CB[n]-based complexes at a reasonable computational cost.[6] Truhlar´s SMD model was used to account for solvation effects in water,[7] and the quadruple zeta valence quality basis set def2-QZVP was used in single point calculations, to more accurately evaluate the energy differences between isomeric species.[8]

In order to predict initial binding modes for $\mathbf{1_a}^{2+}$⊂CB[7,8], hosts and guest were geometrically minimized in vacuum at the PM6 semiempirical level. Then, docking experiments were carried out for each host-guest pair on a 1:1 stoichiometry, employing the program AutoDock Vina,[9] as implemented in UCSF Chimera 1.15.[10] For each docking experiment, cubes of 15 Å^3/1 Å grids centered on the hosts were used as search volume. The best 10 ranked poses obtained for each

[2] Gaussian 16, Revision C.01, Frisch, M. J.; Trucks, G. W.; Schlegel, H. B.; Scuseria, G. E.; Robb, M. A.; Cheeseman, J. R.; Scalmani, G.; Barone, V.; Petersson, G. A.; Nakatsuji, H.; Li, X.; Caricato, M.; Marenich, A. V.; Bloino, J.; Janesko, B. G.; Gomperts, R.; Mennucci, B.; Hratchian, H. P.; Ortiz, J. V.; Izmaylov, A. F.; Sonnenberg, J. L.; Williams-Young, D.; Ding, F.; Lipparini, F.; Egidi, F.; Goings, J.; Peng, B.; Petrone, A.; Henderson, T.; Ranasinghe, D.; Zakrzewski, V. G.; Gao, J.; Rega, N.; Zheng, G.; Liang, W.; Hada, M.; Ehara, M.; Toyota, K.; Fukuda, R.; Hasegawa, J.; Ishida, M.; Nakajima, T.; Honda, Y.; Kitao, O.; Nakai, H.; Vreven, T.; Throssell, K.; Montgomery, J. A., Jr.; Peralta, J. E.; Ogliaro, F.; Bearpark, M. J.; Heyd, J. J.; Brothers, E. N.; Kudin, K. N.; Staroverov, V. N.; Keith, T. A.; Kobayashi, R.; Normand, J.; Raghavachari, K.; Rendell, A. P.; Burant, J. C.; Iyengar, S. S.; Tomasi, J.; Cossi, M.; Millam, J. M.; Klene, M.; Adamo, C.; Cammi, R.; Ochterski, J. W.; Martin, R. L.; Morokuma, K.; Farkas, O.; Foresman, J. B.; Fox, D. J. Gaussian, Inc., Wallingford CT, 2016.

[3] Becke, A. D. Density-functional exchange-energy approximation with correct asymptotic-behavior. *Phys. Rev. A*, **1988**, *38*, 3098.

[4] Miehlich, B.; Savin, A.; Stoll, H.; Preuss, H. Results obtained with the correlation-energy density functionals of Becke and Lee, Yang and Parr. *Chem. Phys. Lett.*, **1989**, *157*, 200.

[5] Grimme, S.; Ehrlich, S.; Goerigk, L. Effect of the damping function in dispersion corrected density functional theory. *J. Comp. Chem.* **2011**, *32*, 1456.

[6] (a) Hostaš, J.; Sigwalt, D.; Šekutor, M.; Ajani, H.; Dubecký, M.; Řezáč, J.; Zavalij, P. Y.; Cao, L.; Wohlschlager, C.; Mlinarić-Majerski, K.; Isaacs, L.; Glaser, R.; P. Hobza. A Nexus between Theory and Experiment: Non-Empirical Quantum Mechanical Computational Methodology Applied to Cucurbit[n]uril·Guest Binding Interactions *Chem. Eur. J.* **2016**, *22*, 17226. (b) Fianchini, M.; Llorens, L.; Pericàs, M. A. Separating Enthalpic, Configurational, and Solvation Entropic Components in Host–Guest Binding: Application to Cucurbit[7]uril Complexes through a Full In Silico Approach via Water Nanodroplets. *J. Phys. Chem. B* **2020,** *124*, 10486-10499.

[7] Marenich, A. V.; Cramer, C. J.; Truhlar, D. G. Universal Solvation Model Based on Solute Electron Density and on a Continuum Model of the Solvent Defined by the Bulk Dielectric Constant and Atomic Surface Tensions. J. Phys. Chem. B **2009**, *113*, 6378.

[8] Weigend, F.; Ahlrichs, R. Balanced basis sets of split valence, triple zeta valence and quadruple zeta valence quality for H to Rn: Design and assessment of accuracy. *Phys. Chem. Chem. Phys.* **2005**, **7,** 3297.

[9] AutoDock Vina 1.2.0: New Docking Methods, Expanded Force Field, and Python Bindings. J. Eberhardt, D. Santos-Martins, A. F. Tillack, S. Forli, *J. Chem. Inform. Model.* **2021**, *61*, 3891.

[10] UCSF Chimera - A Visualization System for Exploratory Research and Analysis. E. F. Pettersen, T. D. Goddard, C. C. Huang, G. S. Couch, D. M. Greenblatt, E. C. Meng, T. E. Ferrin. *J. Comput. Chem.* **2004**,.*25*, 1605.

complex, $1_a^{2+}{\subset}CB[7]$ and $1_a^{2+}{\subset}CB[8]$, where then geometrically optimized at the BLYP-D3(BJ)/def2-SVP/SMD(water) level of theory, resulting in 2 unique conformers for $1_a^{2+}{\subset}CB[7]$ and 4 for $1_a^{2+}{\subset}CB[8]$, which were confirmed as local minima on the corresponding potential energy surface by running frequency calculations at the same level of theory. The total electronic energy of each unique isomer was then calculated at the BLYP-D3(BJ)/def2-QZVP/SMD(water) level of theory, in order to more accurately compare their relative energies. The starting geometry for the [2]rotaxane $2^{4+}{\subset}CB[7]$ was produced with the aid of the software Avogadro,[11] by manually centering the structure of the CB[7] host to the center of mass of the thread 2^{4+}, both minimized at the pm6 semiempirical level of theory. The obtained geometry was firstly re-optimized using the pm6 method to avoid steric clashes and, finally, at the BLYP-D3(BJ)/def2-SVP/SMD(water) level. The later structure was corroborated as a local minimum by running the appropriate frequency calculations at the same level of theory.

[11] Avogadro: An advanced semantic chemical editor, visualization, and analysis platform. M. D. Hanwell, D. E Curtis, D. C. Lonie, T. Vandermeersch, E. Zurek, G. R. Hutchison, *J. Cheminformatics.* **2012**, *4*,17

CARTESIAN COORDINATES

Complex $\mathbf{1_a}^{2+}$⊂CB[7], local minimum $\mathbf{m_1}$ BLYP-D3(BJ)/def2-SVP/SMD (water)

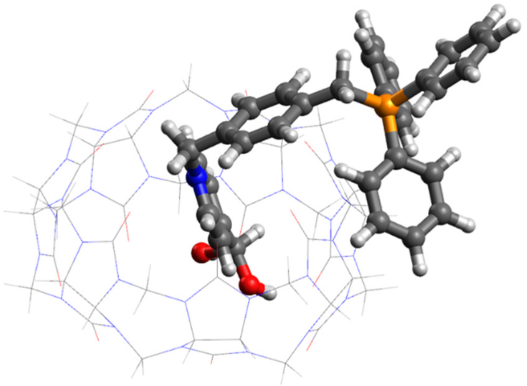

0 imaginary frequencies at BLYP-D3(BJ)/def2-SVP/SMD (water])

Total electronic energy BLYP-D3(BJ)/def2-QZVP/SMD (water): -5997.710368 hartree.

192

P	6.65200	0.56400	-1.04900
C	5.91300	1.01800	-2.69100
H	6.42600	0.32400	-3.38800
H	6.30800	2.03200	-2.89500
C	4.40600	0.96700	-2.86600
C	3.67800	2.15500	-3.10000
C	2.28100	2.12600	-3.25900
H	4.20900	3.11600	-3.14000
C	1.58100	0.90500	-3.20100
H	1.72500	3.06500	-3.39000
C	2.31000	-0.28800	-3.00900
C	3.70100	-0.25800	-2.84300
H	1.79100	-1.25600	-2.95400
H	4.23700	-1.19900	-2.68000
C	0.06500	0.87400	-3.28100
H	-0.29800	0.17000	-4.04700
H	-0.35700	1.87100	-3.48400
N	-0.50900	0.41000	-1.97400
C	-0.73400	-0.92300	-1.77900
C	-1.09700	-1.39300	-0.52400
H	-0.61200	-1.58200	-2.64500
C	-1.24100	-0.49400	0.55400
H	-1.26600	-2.46600	-0.38900
C	-1.59500	-1.05900	1.92400
C	-1.04000	0.87900	0.31400
C	-0.66700	1.30900	-0.96000
H	-1.15700	1.61500	1.11600
H	-0.47200	2.35800	-1.20800
C	6.23300	1.75100	0.26100
C	6.87700	1.61000	1.51500
H	7.61700	0.81500	1.67400
C	6.57000	2.49900	2.55800
H	7.05500	2.37500	3.53600
C	5.65700	3.55000	2.34500
H	5.42000	4.24700	3.16200
C	5.06100	3.71900	1.08300
H	4.37400	4.55500	0.90500
C	5.33400	2.81600	0.04100
H	4.84000	2.94600	-0.92600
C	8.46000	0.67300	-1.27300
C	9.28700	-0.46800	-1.20300
H	8.85700	-1.45800	-1.00700
C	10.67500	-0.32700	-1.38000
H	11.32100	-1.21500	-1.32400
C	11.23400	0.94000	-1.62500
H	12.32000	1.04500	-1.76200
C	10.40600	2.07900	-1.69100
H	10.84200	3.07000	-1.87600
C	9.01900	1.95100	-1.51200
H	8.37400	2.84100	-1.54900
C	6.23400	-1.14400	-0.56800
C	6.29500	-2.16000	-1.55300
C	6.03000	-3.49200	-1.19700
H	6.54700	-1.91600	-2.59300
C	5.72300	-3.82300	0.13700
H	6.06800	-4.27500	-1.96700
C	5.68000	-2.81600	1.11800
H	5.51700	-4.86600	0.41000
C	5.93100	-1.47800	0.77000
H	5.43900	-3.06500	2.16000
H	5.89000	-0.70700	1.54400
O	-7.22300	-0.46900	-0.22600
C	-6.77600	-0.50000	-1.37800
N	-6.71600	0.56900	-2.26400
C	-7.20300	1.89800	-1.93800
N	-6.18900	2.87200	-1.57400
C	-5.76500	3.08100	-0.26500
O	-6.19500	2.50300	0.74000
C	-5.41400	3.64500	-2.52400
H	-6.07400	4.07900	-3.30100
C	-4.69600	4.69600	-1.62700
N	-3.36000	4.75600	-2.19000
C	-2.38700	5.78600	-1.87400
N	-1.61300	5.57700	-0.66400
C	-0.31500	5.08600	-0.67500
O	0.32300	4.72700	-1.67500
H	-5.16200	5.70100	-1.63100
N	-4.79900	4.08700	-0.29000
C	-4.43800	4.80900	0.92000
N	-3.01500	4.80900	1.22100
C	-2.51700	4.21400	2.38000
O	-3.10700	3.39500	3.09300
C	-2.10300	5.82700	0.68500

H	-2.57200	6.82800	0.74400
C	-0.82400	5.65900	1.57500
N	0.15700	5.12300	0.62800
C	1.54300	4.86700	0.96500
N	1.76700	3.63100	1.68700
C	2.14800	2.45300	1.06400
O	2.21500	2.26700	-0.15500
H	-0.45000	6.60100	2.02100
N	-1.25400	4.74300	2.62200
C	-0.50600	4.46700	3.83800
N	0.44800	3.37400	3.74700
C	0.32900	2.19600	4.47800
O	-0.63200	1.86400	5.18200
C	1.77000	3.52200	3.13400
H	2.31400	4.37800	3.58300
C	2.43100	2.13000	3.39300
N	2.47600	1.53200	2.05300
C	3.35000	0.41100	1.75000
N	2.77900	-0.88800	2.05000
H	3.45200	2.18200	3.81900
N	1.51800	1.48800	4.32400
C	1.82400	0.26700	5.04200
N	1.74900	-0.97000	4.27700
C	2.80600	-1.45500	3.40000
H	3.79900	-1.30000	3.86600
C	2.42600	-2.95500	3.16100
H	3.11000	-3.68700	3.63300
N	1.08600	-3.05000	3.75600
C	0.70300	-1.87200	4.38300
O	-0.37600	-1.67200	4.96800
C	0.38200	-4.31100	3.94700
N	-0.32700	-4.82800	2.78900
C	0.28600	-5.60700	1.71600
N	1.05900	-4.83000	0.74800
C	2.40300	-4.34100	1.00400
N	2.46000	-3.08200	1.71500
C	2.61300	-1.86200	1.07200
O	2.62800	-1.68500	-0.15100
H	0.90200	-6.42200	2.14000
C	-0.94300	-6.09300	0.87100
H	-1.13100	-7.18400	0.91800
N	-2.06400	-5.36300	1.47500
C	-1.68200	-4.63500	2.58700
O	-2.43500	-3.95600	3.31300
C	-3.45600	-5.60000	1.13000
N	-3.92500	-4.88800	-0.04900
C	-3.79100	-5.44800	-1.40200
N	-2.48600	-5.29400	-2.01800
C	-1.32800	-6.10000	-1.67500
N	-0.59900	-5.69600	-0.48600
C	0.53000	-4.88600	-0.53600
O	1.00900	-4.35900	-1.54700
H	-4.09700	-6.51300	-1.40700
C	-4.70000	-4.51100	-2.25300
H	-5.36200	-5.04000	-2.96700
N	-5.48700	-3.81100	-1.25500
C	-4.98400	-3.98200	0.03000
O	-5.42700	-3.46300	1.06000
C	-6.62200	-2.96600	-1.56500
N	-6.28900	-1.62900	-2.03900
C	-6.01700	-1.35400	-3.45500
N	-4.65200	-1.60100	-3.89100
C	-4.11900	-2.91800	-4.16600
N	-3.72600	-3.68500	-2.98800
C	-2.43700	-4.21100	-2.88700
O	-1.43700	-3.81100	-3.49800
H	-6.73100	-1.91300	-4.09300
C	-6.15100	0.20000	-3.55100
H	-6.80700	0.55100	-4.37300
N	-4.77200	0.64000	-3.79400
C	-3.92100	-0.43200	-4.04900
O	-2.73600	-0.35700	-4.39800
C	-4.46100	1.98900	-4.23500
N	-4.27800	2.94600	-3.15500
C	-3.10000	3.68000	-3.03000
O	-2.02600	3.44400	-3.59900
H	-7.89400	1.79300	-1.08300
H	-7.75400	2.29300	-2.81300
H	-2.93000	6.74600	-1.78100
H	-1.67300	5.84600	-2.71300
H	-4.96100	4.31800	1.75900
H	-4.78000	5.86200	0.83300
H	1.92700	5.69900	1.58800
H	2.10600	4.82400	0.01700
H	0.03200	5.39400	4.11800
H	-1.22600	4.20300	4.63100
H	4.28700	0.53300	2.32900
H	3.57500	0.43800	0.67200
H	1.10600	0.18700	5.87600
H	2.85400	0.34800	5.44400
H	-0.35700	-4.15500	4.75100
H	1.11700	-5.07700	4.26300
H	2.89400	-4.20200	0.02700
H	2.95500	-5.09800	1.59500
H	-3.59500	-6.68900	0.97200
H	-4.07100	-5.26900	1.98400
H	-1.66800	-7.14400	-1.53400
H	-0.62700	-6.05300	-2.52700
H	-7.23100	-3.48000	-2.33600
H	-7.21400	-2.84800	-0.64200
H	-3.22000	-2.79300	-4.79300
H	-4.88600	-3.49200	-4.72500
H	-5.28200	2.34200	-4.89400
H	-3.51900	1.95200	-4.80800
O	-2.86700	-1.68500	1.81700
O	-1.56300	-0.03400	2.89800

H	-0.82900	-1.82900	2.15900
H	-1.32100	-0.45500	3.75900
H	-2.86100	-2.46700	2.42900

Complex 1_a^{2+}⊂CB[7], local minimum $\mathbf{m_2}$ BLYP-D3(BJ)/def2-SVP/SMD (water).

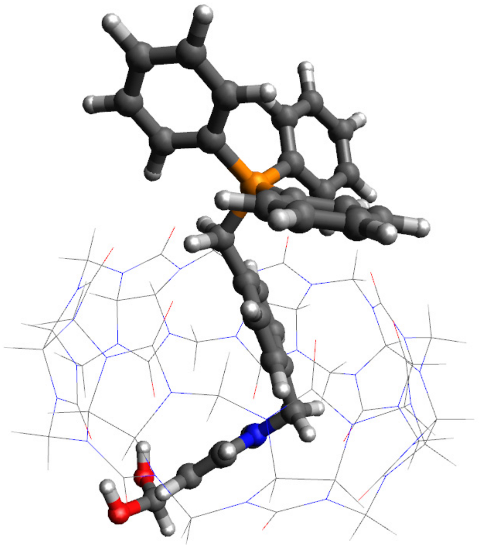

0 imaginary frequencies at BLYP-D3(BJ)/def2-SVP/SMD (water])
Total electronic energy BLYP-D3(BJ)/def2-QZVP/SMD (water): -5997.706052 hartree.
192

C	1.12300	0.12700	-0.54000
C	1.76600	0.31000	-1.89600
N	0.74700	0.52900	-2.97700
C	0.58100	1.77000	-3.51600
C	-0.36400	1.98200	-4.51600
C	-1.14200	0.90700	-4.99000
C	-0.94800	-0.36200	-4.40900
C	-0.01100	-0.52300	-3.39400
C	0.97400	-1.17000	-0.00800
C	0.27200	-1.37600	1.18900
C	-0.30900	-0.28300	1.86700
C	-1.23300	-0.52000	3.03900
P	-0.49000	-0.85200	4.68900
C	0.72300	-2.21000	4.61800
C	2.11400	-1.97200	4.65900
C	3.00400	-3.06100	4.66500
C	2.51300	-4.37800	4.63800
C	1.12600	-4.61300	4.57400
C	0.22700	-3.53500	4.55700
C	-1.84100	-1.39600	5.78100
C	-3.19000	-1.14200	5.45000
C	-4.20700	-1.54800	6.33300
C	-3.88400	-2.19500	7.54000
C	-2.53700	-2.44300	7.86900
C	-1.51300	-2.04700	6.99400
C	0.25100	0.65100	5.39400
C	1.28900	1.32400	4.70700
C	1.86600	2.47100	5.27900
C	1.40500	2.95900	6.51600
C	0.35800	2.30100	7.18800
C	-0.22000	1.14600	6.63400
C	-0.10700	1.02500	1.36900
C	0.60500	1.22800	0.17600
H	2.32800	-0.59100	-2.18100
H	2.43500	1.18100	-1.92400
H	1.23200	2.56500	-3.14000
H	-0.48900	2.99500	-4.91100
H	-1.52500	-1.23500	-4.72900
H	0.16400	-1.48100	-2.89700
H	1.38100	-2.02500	-0.56100
H	0.14000	-2.39100	1.57800
H	-1.86100	-1.41000	2.85100
H	-1.90100	0.34600	3.19300
H	2.50900	-0.95100	4.69500
H	4.08600	-2.87200	4.68800
H	3.21200	-5.22500	4.64900
H	0.74000	-5.64100	4.53000
H	-0.85300	-3.72400	4.50100
H	-3.46200	-0.64800	4.51000
H	-5.25700	-1.35800	6.07100
H	-4.68300	-2.51100	8.22500
H	-2.28200	-2.95000	8.81100
H	-0.46400	-2.24400	7.25300
H	1.65100	0.96800	3.73600
H	2.67400	2.98000	4.74100
H	1.86100	3.85800	6.95500
H	-0.01100	2.68300	8.15000
H	-1.03300	0.63700	7.16500
H	-0.52600	1.87900	1.91400
H	0.73300	2.24300	-0.22200
O	-4.10000	-0.49500	-3.87900
C	-4.63600	-0.70600	-2.78200
N	-4.91300	-1.94900	-2.24100
C	-4.61700	-3.20200	-2.92000
N	-3.36800	-3.83500	-2.53400
C	-2.23200	-3.83100	-3.33400
O	-2.11400	-3.26500	-4.42800
C	-3.22800	-4.69500	-1.36700
H	-4.04800	-5.43900	-1.34600
C	-1.80500	-5.31800	-1.54200
N	-1.14600	-5.03000	-0.27500

C	0.10000	-5.66600	0.12000
N	1.30400	-5.05800	-0.42600
C	2.24200	-4.43100	0.38500
O	2.08500	-4.11900	1.57000
H	-1.81000	-6.41000	-1.72700
N	-1.26800	-4.61900	-2.71300
C	-0.08800	-5.08800	-3.43000
N	1.18100	-4.67300	-2.86200
C	1.97000	-3.66900	-3.41000
O	1.66400	-2.94100	-4.36300
C	1.83500	-5.36300	-1.75600
H	1.82700	-6.45700	-1.93000
C	3.27200	-4.72600	-1.72800
N	3.41000	-4.27600	-0.35000
C	4.63100	-3.74000	0.22500
N	4.90800	-2.34300	-0.06700
C	4.61400	-1.29900	0.80400
O	4.01200	-1.39300	1.88100
H	4.08600	-5.43100	-1.98600
N	3.18900	-3.66600	-2.73800
C	4.34600	-2.94800	-3.24900
N	4.74800	-1.78600	-2.46800
C	4.75900	-0.50000	-3.00500
O	4.24700	-0.15000	-4.07500
C	5.58800	-1.89000	-1.26900
H	6.46900	-2.52900	-1.47400
C	5.93400	-0.39900	-0.95400
N	5.16700	-0.13600	0.27000
C	5.39200	1.04800	1.08000
N	4.68200	2.23500	0.63100
H	7.01100	-0.20900	-0.77700
N	5.48600	0.31300	-2.14100
C	5.82300	1.69700	-2.43600
N	5.02100	2.71200	-1.77000
C	5.22100	3.13800	-0.39300
H	6.29400	3.33600	-0.20300
C	4.30100	4.39700	-0.26400
H	4.84700	5.35900	-0.22000
N	3.48600	4.33200	-1.48400
C	3.96400	3.37800	-2.37900
O	3.54600	3.18200	-3.52800
C	2.64000	5.43800	-1.91000
N	1.30300	5.43800	-1.34000
C	0.98000	6.02200	-0.03500
N	1.37400	5.24500	1.13200
C	2.73700	5.15500	1.62600
N	3.58200	4.16500	0.97800
C	3.75500	2.87100	1.45100
O	3.22000	2.38200	2.45200
H	1.39700	7.04700	0.03300
C	-0.58600	5.96400	0.00500
H	-1.07000	6.93900	0.21300
N	-0.94500	5.50100	-1.32900

C	0.16700	5.20800	-2.11000
O	0.15100	4.85200	-3.29500
C	-2.30000	5.52200	-1.84800
N	-3.14200	4.40900	-1.43500
C	-3.85700	4.36500	-0.16500
N	-3.06200	3.94900	0.99000
C	-2.18200	4.87000	1.69200
N	-0.86400	5.02500	1.09600
C	0.29200	4.66800	1.78400
O	0.33900	3.99900	2.82300
H	-4.33600	5.34400	0.03400
C	-4.86500	3.18400	-0.35200
H	-5.92900	3.49200	-0.37200
N	-4.48500	2.62100	-1.65100
C	-3.51500	3.38100	-2.28600
O	-3.07200	3.20600	-3.43100
C	-5.30500	1.64200	-2.34400
N	-5.10000	0.26800	-1.90800
C	-5.83800	-0.32200	-0.78200
N	-5.32800	-0.01100	0.54400
C	-5.51600	1.26500	1.21300
N	-4.61200	2.32900	0.80000
C	-3.55700	2.78900	1.58200
O	-3.15500	2.28100	2.63700
H	-6.91000	-0.05300	-0.85500
C	-5.54900	-1.85400	-0.93400
H	-6.45300	-2.49300	-0.90400
N	-4.68900	-2.14200	0.21900
C	-4.65300	-1.08100	1.11700
O	-4.14700	-1.10100	2.24500
C	-4.31700	-3.49500	0.60700
N	-3.14100	-4.01400	-0.07400
C	-1.94900	-4.29300	0.58800
O	-1.67200	-3.97400	1.75300
H	-4.56700	-2.99700	-4.00300
H	-5.44700	-3.90500	-2.71300
H	0.06500	-6.72700	-0.20000
H	0.17100	-5.61300	1.22000
H	-0.14800	-4.68900	-4.45600
H	-0.10600	-6.19700	-3.45900
H	5.48400	-4.33900	-0.14800
H	4.55400	-3.84900	1.32000
H	5.20000	-3.65500	-3.30200
H	4.09700	-2.59000	-4.26300
H	6.48000	1.27000	1.09100
H	5.05100	0.82100	2.10400
H	5.70300	1.84200	-3.52300
H	6.88100	1.85900	-2.15400
H	2.53300	5.37400	-3.00700
H	3.13900	6.39100	-1.64000
H	2.68600	4.90900	2.70000
H	3.21800	6.14400	1.49800
H	-2.78800	6.46100	-1.51800

H	-2.23700	5.50500	-2.94900
H	-2.67200	5.86500	1.73200
H	-2.04000	4.48500	2.71600
H	-6.37200	1.90500	-2.19400
H	-5.06000	1.69600	-3.41900
H	-5.37200	1.10100	2.29500
H	-6.55300	1.60400	1.02700
H	-5.17000	-4.17200	0.40000
H	-4.10700	-3.48800	1.69000
C	-2.14100	1.11400	-6.13700
O	-2.99700	0.00300	-6.32000
H	-1.55300	1.18900	-7.07800
O	-2.83000	2.34500	-6.00400
H	-3.04800	2.49800	-5.04900
H	-3.45900	-0.17000	-5.46200

Rotaxane 2^{4+}⊂CB[7], local minimum at BLYP-D3(BJ)/def2-SVP/SMD (water).

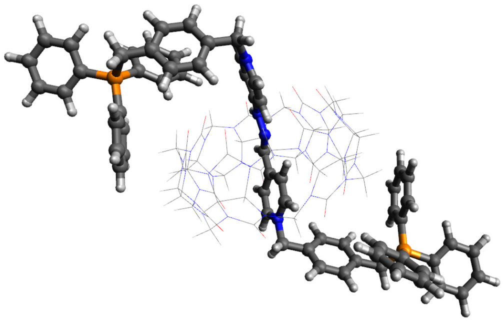

0 imaginary frequencies at BLYP-D3(BJ)/def2-SVP/SMD (water]), Total electronic energy BLYP-D3(BJ)/def2-SVP/SMD (water): -7541.4926 hartree.
251

C	-2.63300	-0.37000	1.48700
C	-2.05600	0.71800	0.59800
N	-1.39100	0.14500	-0.60200
C	-1.98500	0.23900	-1.82400
C	-1.40000	-0.33500	-2.94500
C	-0.15000	-0.98500	-2.82800
C	0.54200	-1.45700	-4.02300
N	1.80200	-1.75200	-3.89800
N	2.53300	-2.09200	-4.96500
C	3.89800	-2.14800	-4.81500
C	4.72900	-2.43000	-5.93300
C	6.10400	-2.39200	-5.77500
N	6.67200	-2.12800	-4.55700
C	8.14700	-1.91100	-4.45500
C	8.50800	-0.48200	-4.81900
C	8.16700	0.59300	-3.96700
C	8.46600	1.91400	-4.33600
C	9.11700	2.18500	-5.56000
C	9.34700	3.59900	-6.03900
P	7.83800	4.27500	-6.89200
C	8.35400	5.56400	-8.07100
C	7.65400	6.79000	-8.13900
C	8.05000	7.76600	-9.07000
C	9.13700	7.52500	-9.93100
C	9.83600	6.30500	-9.86000
C	9.45200	5.32300	-8.93200
C	6.66800	4.98500	-5.69900
C	5.33000	5.19400	-6.11000
C	4.44100	5.86900	-5.25800
C	4.87200	6.31700	-3.99500
C	6.19700	6.09100	-3.58100
C	7.10100	5.43200	-4.43100
C	7.04500	2.87500	-7.73900
C	6.24600	2.00500	-6.96000
C	5.77900	0.80500	-7.51900
C	6.09700	0.47500	-8.85000
C	6.87700	1.35100	-9.62900
C	7.35800	2.55300	-9.07800
C	9.45700	1.10900	-6.40800
C	9.15300	-0.21100	-6.04300
C	5.88700	-1.89000	-3.46200
C	4.51100	-1.89200	-3.55700
C	0.43000	-1.09000	-1.53200
C	-0.21000	-0.52900	-0.44000
C	-3.20500	-1.54100	0.94600
C	-3.73400	-2.52800	1.79300
C	-3.72400	-2.35700	3.19400
C	-4.26900	-3.43100	4.10700
P	-3.00100	-4.60400	4.78600
C	-2.17800	-5.54200	3.46800
C	-0.83800	-5.95700	3.64200
C	-0.24100	-6.79300	2.68300
C	-0.97600	-7.22000	1.56300
C	-2.31300	-6.80900	1.39500
C	-2.91900	-5.97200	2.34400
C	-3.88100	-5.78000	5.86100
C	-4.80600	-5.30000	6.81900
C	-5.47500	-6.21000	7.65400
C	-5.22500	-7.59200	7.54000
C	-4.30400	-8.06600	6.58800
C	-3.62900	-7.16600	5.74600
C	-1.75100	-3.70300	5.75000

C	-0.85600	-2.83800	5.07500	H	-5.75200	-8.30000	8.19500
C	0.10300	-2.12300	5.81200	H	-4.10800	-9.14400	6.49700
C	0.17700	-2.27100	7.21000	H	-2.91200	-7.54000	5.00400
C	-0.71000	-3.13600	7.87800	H	-0.90300	-2.72100	3.98700
C	-1.67600	-3.85500	7.15300	H	0.79000	-1.44800	5.28500
C	-3.16800	-1.17400	3.73100	H	0.93200	-1.71100	7.78100
C	-2.61400	-0.19900	2.88600	H	-0.65000	-3.25700	8.96800
H	0.01000	-1.45900	-4.99100	H	-2.36100	-4.53200	7.67800
H	2.11400	-2.21300	-5.91600	H	-3.15300	-1.02700	4.81900
H	-1.30300	1.31500	1.14000	H	-2.15400	0.70200	3.31600
H	-2.84300	1.40300	0.23200	O	1.54800	-2.56600	-7.69000
H	-2.93200	0.78800	-1.86800	C	0.31100	-2.50400	-7.81600
H	-1.91700	-0.25900	-3.90700	N	-0.57900	-3.52900	-7.55900
H	4.29200	-2.64300	-6.91500	C	-0.13600	-4.89100	-7.29700
H	6.79700	-2.55600	-6.60900	N	0.03600	-5.21600	-5.89800
H	8.63600	-2.62900	-5.13400	C	1.27300	-5.27700	-5.26900
H	8.43200	-2.14900	-3.41600	O	2.37500	-5.09100	-5.80100
H	7.64900	0.40700	-3.01600	C	-1.07100	-5.56200	-5.01700
H	8.17500	2.74300	-3.67700	H	-1.64800	-6.40500	-5.44600
H	9.61000	4.30500	-5.23100	C	-0.36000	-5.89100	-3.66600
H	10.14900	3.63600	-6.79900	N	-0.97400	-4.96900	-2.71300
H	6.80700	6.98700	-7.47000	C	-0.98500	-5.26600	-1.29000
H	7.50500	8.71900	-9.11900	N	0.25300	-4.94200	-0.59900
H	9.44300	8.29200	-10.65700	C	0.24900	-4.20600	0.58400
H	10.68800	6.11500	-10.52800	O	-0.68300	-3.51600	1.01000
H	10.00700	4.37700	-8.88300	H	-0.50100	-6.93600	-3.32600
H	4.98700	4.84000	-7.09000	N	1.05300	-5.64000	-3.94300
H	3.40700	6.04300	-5.58100	C	2.10700	-6.21300	-3.12200
H	4.17000	6.83800	-3.33000	N	2.29800	-5.54400	-1.84900
H	6.53400	6.43000	-2.59200	C	3.52800	-5.00400	-1.47400
H	8.13600	5.27700	-4.10200	O	4.47200	-4.75100	-2.23400
H	5.99300	2.24600	-5.92200	C	1.43600	-5.80300	-0.70200
H	5.15900	0.13800	-6.91100	H	1.13000	-6.86800	-0.68500
H	5.73100	-0.46700	-9.28200	C	2.30500	-5.37500	0.52300
H	7.11700	1.10000	-10.67100	N	1.47200	-4.40200	1.21300
H	7.97400	3.22600	-9.68800	C	1.73100	-3.98400	2.58000
H	9.94300	1.31400	-7.37200	N	2.59000	-2.82300	2.71600
H	9.41400	-1.04200	-6.71400	C	2.09700	-1.52700	2.76600
H	6.39800	-1.69900	-2.51300	O	0.90300	-1.19800	2.76000
H	3.91100	-1.69400	-2.66600	H	2.56100	-6.20700	1.21000
H	1.37900	-1.61500	-1.38300	N	3.51500	-4.84100	-0.09600
H	0.18100	-0.59100	0.58400	C	4.67500	-4.42900	0.67000
H	-3.20900	-1.71100	-0.14000	N	4.57300	-3.10100	1.26100
H	-4.15800	-3.44400	1.36000	C	5.36500	-2.04800	0.81700
H	-5.01400	-4.07500	3.60000	O	5.98800	-2.01000	-0.25200
H	-4.74600	-2.99600	5.00500	C	4.04200	-2.86200	2.61000
H	-0.26500	-5.63400	4.52000	H	4.46100	-3.60200	3.32000
H	0.80300	-7.10900	2.81200	C	4.46100	-1.38000	2.89800
H	-0.50400	-7.87000	0.81300	N	3.18100	-0.66300	2.87400
H	-2.88600	-7.13900	0.51800	C	3.02000	0.69600	3.37600
H	-3.96500	-5.66600	2.21300	N	3.13900	1.74000	2.37700
H	-5.00100	-4.22400	6.92300	H	4.96200	-1.22900	3.87400
H	-6.19300	-5.83700	8.39800	N	5.37500	-1.07300	1.80500

C	6.00900	0.21900	1.61900	N	-1.82600	1.43500	-6.75100
N	5.16100	1.24800	1.02600	C	-1.93100	2.03000	-5.49900
C	4.40800	2.21100	1.84300	O	-2.88000	1.91600	-4.71300
H	5.05000	2.59400	2.66000	H	-2.29700	-1.47300	-9.23700
C	3.97800	3.29700	0.80400	C	-1.97200	-3.11300	-7.70700
H	4.17200	4.33900	1.12500	H	-2.50300	-3.80000	-8.39300
N	4.77700	2.97100	-0.36600	N	-2.72000	-2.95800	-6.45800
C	5.41400	1.74200	-0.25300	C	-3.03000	-1.63500	-6.17800
O	6.11700	1.20500	-1.11800	O	-3.65500	-1.22800	-5.19000
C	4.81700	3.77700	-1.57200	C	-3.05500	-4.03900	-5.55100
N	3.68400	3.60400	-2.46600	N	-1.97800	-4.47100	-4.66900
C	2.45600	4.37600	-2.34300	C	-1.95000	-4.17100	-3.30900
N	1.47900	3.86000	-1.38700	O	-2.68900	-3.36800	-2.72600
C	1.63800	4.04100	0.04300	H	0.83900	-5.03600	-7.79200
N	2.52700	3.07700	0.67600	H	-0.88100	-5.58000	-7.73800
C	2.05300	2.19500	1.64100	H	-1.19100	-6.34900	-1.16900
O	0.86600	1.90500	1.84200	H	-1.79400	-4.68300	-0.82000
H	2.69600	5.43200	-2.10800	H	3.04900	-6.13500	-3.68900
C	1.75000	4.16900	-3.71900	H	1.87300	-7.28200	-2.93100
H	1.66200	5.09300	-4.32300	H	2.20300	-4.82500	3.12100
N	2.61400	3.20200	-4.40200	H	0.76700	-3.74700	3.05300
C	3.78700	2.95800	-3.69100	H	4.84400	-5.16100	1.48600
O	4.75700	2.29400	-4.07800	H	5.54100	-4.43000	-0.01400
C	2.53400	3.01600	-5.84200	H	3.78300	0.87400	4.15800
N	1.39300	2.22700	-6.27800	H	2.01300	0.77600	3.82000
C	0.07600	2.82800	-6.47700	H	6.87500	0.07200	0.95200
N	-0.79400	2.80900	-5.30300	H	6.36000	0.57600	2.60600
C	-0.71500	3.81900	-4.26200	H	5.73100	3.51100	-2.12900
N	0.42200	3.67700	-3.36500	H	4.86300	4.84000	-1.27000
C	0.27100	3.52400	-1.98700	H	0.64300	3.94200	0.50900
O	-0.76600	3.18200	-1.40200	H	2.03500	5.06000	0.22800
H	0.18600	3.86500	-6.85400	H	2.47200	4.01000	-6.33000
C	-0.63400	1.86000	-7.47900	H	3.45400	2.50400	-6.16900
H	-0.92500	2.33100	-8.43900	H	-0.66200	4.82200	-4.73700
N	0.37100	0.83200	-7.71500	H	-1.63600	3.74500	-3.65800
C	1.55300	1.05900	-7.02000	H	-0.34400	0.21600	-9.57400
O	2.57900	0.37300	-7.08600	H	1.25300	-0.46200	-9.07700
C	0.23900	-0.19700	-8.73000	H	-3.78600	0.76800	-6.77100
N	-0.40500	-1.42000	-8.28700	H	-2.98100	0.83300	-8.38600
C	-1.85300	-1.63400	-8.23700	H	-3.38400	-4.91200	-6.14600
N	-2.57200	-0.85500	-7.23200	H	-3.88900	-3.68700	-4.91900
C	-2.84800	0.57200	-7.31900				

Complex $\mathbf{1_a}^{2+}\subset$CB[8], local minimum $\mathbf{m'_1}$ BLYP-D3(BJ)/def2-SVP/SMD (water).

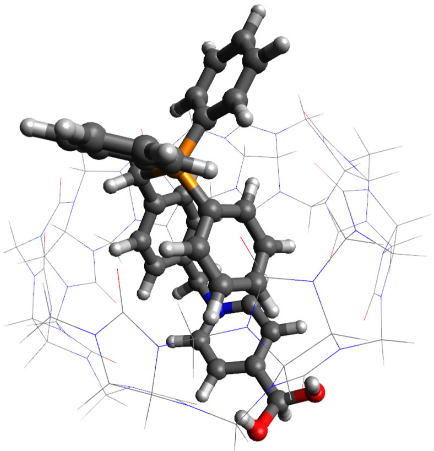

0 imaginary frequencies at BLYP-D3(BJ)/def2-SVP/SMD (water])

Total electronic energy BLYP-D3(BJ)/def2-QZVP/SMD (water): -6592.179592 hartree.

210

C	6.57900	0.72200	0.94200
H	7.66700	0.53200	1.02400
N	6.12800	0.47300	-0.43300
C	5.64800	1.61800	-1.04500
O	5.25800	1.68900	-2.22000
N	5.74800	2.66300	-0.14700
C	5.55800	4.05600	-0.50900
N	4.27000	4.62200	-0.16700
C	3.28500	4.91000	-1.10100
O	3.31300	4.61900	-2.30400
N	2.29200	5.64100	-0.45800
C	1.36200	6.47200	-1.20400
N	-0.04000	6.14700	-1.03400
C	-0.77500	5.39900	-1.94300
O	-0.33700	4.86600	-2.97200
N	-2.10600	5.39300	-1.51500
C	-3.18600	5.22900	-2.48000
N	-4.17700	4.22800	-2.11600
C	-4.52300	3.21200	-3.00600
O	-3.84500	2.83000	-3.97000
N	-5.78000	2.74200	-2.65300
C	-6.49200	1.71600	-3.39600
N	-6.30500	0.35500	-2.92700
C	-5.57600	-0.58800	-3.64200
O	-4.91500	-0.37700	-4.66700
N	-5.77200	-1.82300	-3.03600
C	-5.41000	-3.07500	-3.67200
N	-4.36000	-3.82300	-3.00400
C	-3.09800	-4.01100	-3.55000
O	-2.63300	-3.42100	-4.53500
N	-2.47300	-5.02600	-2.83400
C	-1.27700	-5.69100	-3.31800
N	-0.07000	-5.42600	-2.55600
C	0.91000	-4.53500	-2.96900
O	0.87500	-3.83200	-3.98700
N	1.96500	-4.60600	-2.05800
C	3.31600	-4.23700	-2.45300
N	3.99900	-3.36100	-1.51500
C	4.57100	-2.16100	-1.92400
O	4.25500	-1.52800	-2.94300
N	5.57600	-1.83600	-1.02900
C	5.67600	-2.77700	0.08000
H	6.71600	-3.14800	0.16800
N	5.20700	-2.28900	1.37600
C	5.99100	-1.41800	2.24000
N	5.85900	0.00500	1.98300
C	4.93800	0.81000	2.64200
O	4.17200	0.44700	3.54300
N	5.07400	2.10400	2.15400
C	4.52300	3.25200	2.85500
N	3.53500	4.01300	2.10300
C	2.22400	4.16600	2.55100
O	1.66500	3.47400	3.41000
N	1.67700	5.26700	1.90100
C	0.40800	5.86400	2.26700
N	-0.67500	5.66800	1.30600
C	-1.91600	5.19000	1.73700
O	-2.13000	4.55300	2.77400
N	-2.87300	5.59800	0.81800
C	-4.30200	5.43500	1.01300
N	-4.93000	4.39000	0.22700
C	-5.38600	3.19600	0.77000
O	-5.20800	2.80700	1.93100
N	-6.12100	2.53800	-0.21000
C	-7.00500	1.43200	0.12000
N	-6.64100	0.16300	-0.48100
C	-6.03500	-0.86700	0.22600
O	-5.62600	-0.80900	1.39400
N	-6.02100	-1.99200	-0.59100
C	-5.82200	-3.33200	-0.06400
N	-4.64100	-4.01600	-0.56400
C	-3.63000	-4.45500	0.28200
O	-3.47200	-4.12500	1.46500
N	-2.86600	-5.38300	-0.41400
C	-1.79000	-6.13500	0.20000
N	-0.44800	-5.67000	-0.12100
C	0.40500	-5.15200	0.85000
O	0.08200	-4.79900	1.99200
N	1.69300	-5.14300	0.32500
C	2.88200	-5.05100	1.15500
N	3.74200	-3.91800	0.86400
C	4.09500	-2.98700	1.83600
O	3.54400	-2.84300	2.93300

C	4.62500	-3.87600	-0.29300
H	5.06300	-4.87500	-0.48400
C	1.73600	-5.64000	-1.04300
H	2.49300	-6.44400	-1.13800
C	0.27200	-6.11100	-1.31800
H	0.15700	-7.20500	-1.44500
C	-3.32600	-5.58200	-1.78300
H	-3.50400	-6.65900	-1.97200
C	-4.61300	-4.69200	-1.86200
H	-5.55000	-5.26200	-2.01600
C	-6.66200	-1.74800	-1.88400
H	-7.51900	-2.43600	-2.01800
C	-7.05800	-0.23500	-1.82100
H	-8.14000	-0.04000	-1.95400
C	-6.27200	3.34700	-1.42500
H	-7.32700	3.65900	-1.54800
C	-5.27300	4.52400	-1.17900
H	-5.69100	5.52900	-1.38300
C	-2.28500	6.25400	-0.33200
H	-2.86200	7.16100	-0.60200
C	-0.82300	6.53500	0.12300
H	-0.62000	7.59000	0.39400
C	2.60000	5.87700	0.95400
H	2.68700	6.96400	1.14900
C	3.92500	5.07500	1.17200
H	4.75400	5.67100	1.60100
C	6.16100	2.21400	1.17500
H	6.97000	2.86100	1.56500
C	6.47400	-0.71200	-1.20000
H	5.68600	4.14500	-1.60100
H	6.34400	4.64500	0.00200
H	1.60800	6.36600	-2.27400
H	1.50200	7.52800	-0.89500
H	-3.69800	6.20600	-2.61100
H	-2.73700	4.91900	-3.43800
H	-6.14200	1.75300	-4.44200
H	-7.57100	1.96100	-3.35800
H	-5.06200	-2.84300	-4.69300
H	-6.31200	-3.71900	-3.72400
H	-1.10000	-5.35600	-4.35500
H	-1.45500	-6.78500	-3.30800
H	3.25100	-3.71300	-3.42100
H	3.92000	-5.16300	-2.57300
H	7.06000	-1.68800	2.13300
H	5.66600	-1.60300	3.27800
H	5.35400	3.93500	3.12900
H	4.03600	2.87800	3.77100
H	0.56800	6.95400	2.40700
H	0.08500	5.41300	3.22100
H	-4.79300	6.39700	0.76500
H	-4.47000	5.20000	2.07800
H	-8.03400	1.68600	-0.20600
H	-6.98500	1.30700	1.21600
H	-6.71600	-3.94200	-0.31000
H	-5.71800	-3.24900	1.03100
H	-1.91500	-6.06600	1.29400
H	-1.87800	-7.19300	-0.12000
H	3.47000	-5.98500	1.02900
H	2.55400	-4.95900	2.20500
H	6.47600	-0.43300	-2.26700
H	7.49000	-1.03700	-0.90300
C	2.17800	0.97700	-4.61600
C	1.28600	1.92700	-4.07400
C	-0.03400	1.56900	-3.81200
C	1.70400	-0.33400	-4.83100
C	0.37500	-0.64600	-4.56200
N	-0.47500	0.30700	-4.08300
C	-1.88900	-0.06800	-3.74400
C	-1.98400	-0.47200	-2.28700
C	-2.66700	0.33900	-1.35900
C	-2.69000	0.00200	0.00500
C	-2.00300	-1.14300	0.46200
C	-1.87300	-1.47500	1.92900
P	-0.69500	-0.33000	2.79400
C	0.42500	0.38900	1.55100
C	0.07400	1.59300	0.89600
C	0.83700	2.03000	-0.20300
C	1.95500	1.28800	-0.63200
C	2.30300	0.09300	0.02700
C	1.53900	-0.36700	1.11400
C	-1.60900	0.96200	3.70000
C	-0.86600	1.83800	4.52700
C	-1.53500	2.74700	5.35900
C	-2.94300	2.79300	5.37000
C	-3.67900	1.94500	4.52500
C	-3.02000	1.02600	3.68600
C	0.28100	-1.27800	4.00300
C	1.46500	-0.70500	4.52100
C	2.20400	-1.39900	5.49200
C	1.77300	-2.66100	5.94200
C	0.59500	-3.23000	5.42300
C	-0.15900	-2.54200	4.45600
C	-1.31900	-1.95200	-0.47100
C	-1.32300	-1.63300	-1.83400
H	1.61300	2.95000	-3.85600
H	-0.77400	2.27100	-3.40900
H	2.36400	-1.11600	-5.22200
H	-0.04800	-1.64400	-4.72400
H	-2.16600	-0.90100	-4.41100
H	-2.52400	0.80400	-3.96600
H	-3.15700	1.25500	-1.70700
H	-3.21300	0.65500	0.71700
H	-1.44400	-2.48400	2.04700
H	-2.83600	-1.44600	2.47000
H	-0.79400	2.17400	1.23500
H	0.55500	2.94700	-0.73600

H	2.55400	1.63700	-1.48300	H	0.26000	-4.21800	5.76700
H	3.16600	-0.48800	-0.31500	H	-1.07700	-2.99700	4.06400
H	1.80300	-1.30800	1.61400	H	-0.77500	-2.83600	-0.11600
H	0.22900	1.82000	4.51900	H	-0.78900	-2.27700	-2.54300
H	-0.95300	3.42600	5.99700	C	3.58700	1.38200	-5.07500
H	-3.46500	3.50300	6.02500	O	4.03900	2.57500	-4.46800
H	-4.77500	1.99800	4.50400	H	3.48700	1.62500	-6.15700
H	-3.61900	0.38000	3.03400	O	4.50700	0.31000	-5.00400
H	1.83200	0.25800	4.15000	H	4.31400	2.36500	-3.53800
H	3.13500	-0.95800	5.87400	H	4.43400	-0.14700	-4.12700
H	2.36100	-3.20700	6.69400				

Complex 1_a^{2+}⊂CB[8], local minimum **m'$_2$** BLYP-D3(BJ)/def2-SVP/SMD (water).

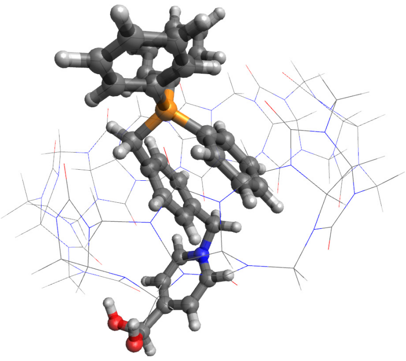

0 imaginary frequencies at BLYP-D3(BJ)/def2-SVP/SMD (water])
Total electronic energy BLYP-D3(BJ)/def2-QZVP/SMD (water): -6592.201832 hartree.
210

C	-0.75300	-0.14000	-0.01600
H	-1.07600	-0.15400	1.04400
N	0.69800	-0.15300	-0.10000
C	1.19300	0.80900	-0.97000
O	2.38300	0.99200	-1.25600
N	0.11600	1.57400	-1.41500
C	0.32700	2.91800	-1.92900
N	-0.27400	3.19100	-3.21900
C	0.44700	3.31900	-4.40000
O	1.65300	3.08300	-4.54700
N	-0.42100	3.80600	-5.38300
C	0.10100	4.56600	-6.51300
N	-0.35200	4.10100	-7.81400
C	0.55200	3.80300	-8.83100
O	1.76500	3.59000	-8.69200
N	-0.13300	3.83500	-10.03800
C	0.49100	3.58900	-11.32300
N	0.37200	2.22300	-11.82000
C	1.50400	1.46000	-12.10200
O	2.65400	1.68600	-11.70400
N	1.12100	0.42700	-12.94800
C	2.05400	-0.51400	-13.53800
N	2.12800	-1.81200	-12.88900
C	3.14400	-2.17600	-12.02400
O	4.07400	-1.43800	-11.66300
N	2.96200	-3.50400	-11.66600
C	4.05100	-4.29200	-11.10300
N	3.74600	-4.92500	-9.83300
C	4.20900	-4.47000	-8.61100
O	4.84600	-3.42000	-8.41700
N	3.88500	-5.41100	-7.64000
C	4.61600	-5.47100	-6.38100
N	3.77600	-5.53200	-5.19400
C	4.03600	-4.69100	-4.10400
O	4.59700	-3.59000	-4.15400
N	3.58700	-5.33200	-2.95600
C	3.79700	-4.82400	-1.61300
N	2.68200	-4.08700	-1.04500
C	2.77600	-2.72700	-0.77000
O	3.67900	-1.96800	-1.14300
N	1.70800	-2.38900	0.05100
C	0.78200	-3.49100	0.24600
H	0.53700	-3.60100	1.32000
N	-0.45200	-3.43500	-0.54900
C	-1.56400	-2.55100	-0.24400
N	-1.44100	-1.19700	-0.76400
C	-2.29600	-0.68300	-1.73700
O	-3.06800	-1.34000	-2.44700
N	-2.17000	0.70400	-1.72500
C	-3.09200	1.60600	-2.38800
N	-2.55500	2.30400	-3.55600
C	-3.33500	2.42100	-4.71200
O	-4.29000	1.70200	-5.02300
N	-2.87600	3.51800	-5.43000
C	-3.50600	4.01400	-6.63900
N	-2.79900	3.72600	-7.87500
C	-3.22900	2.77700	-8.79300
O	-4.18400	2.00700	-8.64700
N	-2.41600	2.87600	-9.92600
C	-2.86700	2.34000	-11.20600
N	-1.96100	1.39400	-11.82400
C	-2.09600	0.01600	-11.71300
O	-2.98200	-0.57900	-11.08600
N	-1.09100	-0.57400	-12.47300
C	-1.17300	-1.96200	-12.89800
N	-0.09000	-2.80300	-12.41100
C	-0.34100	-3.95300	-11.66500
O	-1.41000	-4.24900	-11.11500
N	0.80900	-4.73400	-11.68800
C	0.89200	-6.05600	-11.10300
N	1.57900	-6.11300	-9.81900
C	0.95800	-6.65200	-8.69400
O	-0.25200	-6.87600	-8.56900
N	1.94200	-6.93600	-7.75400
C	1.69500	-7.76400	-6.58500
N	1.88200	-7.09600	-5.31100
C	0.86300	-6.92100	-4.37900
O	-0.34400	-7.09900	-4.58200
N	1.45100	-6.55600	-3.17100
C	0.71900	-6.60100	-1.91200
N	0.54900	-5.32500	-1.24300
C	-0.59200	-4.54200	-1.37600
O	-1.58300	-4.81800	-2.06400
C	1.54200	-4.71700	-0.37000
H	1.90000	-5.45300	0.37600
C	2.91500	-6.58800	-3.23700

H	3.30800	-7.38800	-2.57800
C	3.19500	-6.80900	-4.75700
H	3.89600	-7.64000	-4.97100
C	3.27500	-6.61000	-8.23600
H	3.95300	-7.47800	-8.11000
C	3.04000	-6.19200	-9.72200
H	3.43300	-6.91200	-10.46700
C	1.88000	-4.12400	-12.45300
H	2.30800	-4.85500	-13.16600
C	1.19000	-2.89700	-13.12600
H	1.01000	-3.01500	-14.21300
C	-0.30900	0.42500	-13.21400
H	-0.49600	0.32400	-14.30100
C	-0.79300	1.77700	-12.59900
H	-1.06500	2.54700	-13.34800
C	-1.55500	4.06800	-9.85800
H	-1.91200	4.83400	-10.57400
C	-1.66400	4.49000	-8.36000
H	-1.83300	5.57400	-8.20300
C	-1.73200	4.14900	-4.80400
H	-1.86000	5.25000	-4.78200
C	-1.68100	3.48200	-3.39900
H	-2.05300	4.12400	-2.57600
C	-1.14700	1.15900	-0.79300
H	-1.54500	1.97100	-0.15300
C	1.54800	-1.07100	0.63500
H	1.41700	3.06200	-2.02600
H	-0.08300	3.65100	-1.20300
H	1.20200	4.48800	-6.49500
H	-0.19400	5.63000	-6.39100
H	0.03200	4.27900	-12.06000
H	1.56600	3.81500	-11.22800
H	3.06000	-0.06300	-13.50100
H	1.76000	-0.67500	-14.59300
H	4.91500	-3.62200	-10.96000
H	4.32500	-5.09100	-11.82100
H	5.23200	-4.55800	-6.31300
H	5.27700	-6.36500	-6.39800
H	4.66200	-4.13900	-1.64000
H	4.02700	-5.68800	-0.95800
H	-1.67600	-2.49000	0.85800
H	-2.47300	-2.99900	-0.68100
H	-3.42900	2.35800	-1.64300
H	-3.95900	1.01700	-2.73100
H	-3.61000	5.11300	-6.54900
H	-4.50500	3.55100	-6.71000
H	-3.02000	3.17800	-11.91500
H	-3.82800	1.82800	-11.02900
H	-1.18000	-1.99400	-14.00800
H	-2.11900	-2.37800	-12.51200
H	1.41700	-6.71800	-11.82100
H	-0.13500	-6.42500	-10.94300
H	0.64800	-8.10800	-6.63400
H	2.37800	-8.63900	-6.62700
H	1.25000	-7.28200	-1.21600
H	-0.28400	-7.00500	-2.13200
H	2.54900	-0.61100	0.70400
H	1.12500	-1.19500	1.65000
C	6.15300	-0.42400	-9.18500
C	5.25800	0.49900	-9.76300
C	4.22300	1.02900	-9.00000
C	6.07700	-0.64000	-7.79300
C	5.03800	-0.07500	-7.06300
N	4.10200	0.70200	-7.68200
C	2.77700	0.92400	-7.02000
C	1.83800	-0.19100	-7.45700
C	0.65600	0.09700	-8.16700
C	-0.25300	-0.92900	-8.49000
C	0.02700	-2.26300	-8.12500
C	-1.03300	-3.34000	-8.13600
P	-2.06000	-3.17000	-6.59100
C	-0.95700	-2.51200	-5.30700
C	-0.77500	-1.11600	-5.17600
C	0.31300	-0.63100	-4.43000
C	1.19500	-1.52500	-3.79400
C	0.98100	-2.91300	-3.89000
C	-0.08400	-3.41400	-4.65500
C	-3.47400	-2.06800	-6.87800
C	-3.87700	-1.73600	-8.19000
C	-5.05700	-0.99800	-8.38600
C	-5.83600	-0.60400	-7.28400
C	-5.43100	-0.93700	-5.97700
C	-4.24700	-1.66000	-5.76400
C	-2.69000	-4.79200	-6.07200
C	-3.13900	-4.95500	-4.74200
C	-3.63400	-6.20100	-4.32800
C	-3.69200	-7.27600	-5.23600
C	-3.25700	-7.10600	-6.56300
C	-2.75700	-5.86300	-6.99000
C	1.26300	-2.55800	-7.50600
C	2.15300	-1.53600	-7.16200
H	5.30300	0.74200	-10.82900
H	3.43800	1.66500	-9.42400
H	6.77800	-1.31300	-7.28700
H	4.88500	-0.26700	-5.99500
H	2.40400	1.91400	-7.32500
H	2.94600	0.91400	-5.92900
H	0.41700	1.13400	-8.42800
H	-1.20500	-0.68100	-8.97700
H	-0.58200	-4.34500	-8.07400
H	-1.70100	-3.31900	-9.01400
H	-1.45400	-0.41600	-5.68000
H	0.48500	0.45000	-4.35600
H	2.04500	-1.14100	-3.21400
H	1.64300	-3.61100	-3.36900
H	-0.22900	-4.49500	-4.75700

H	-3.28500	-2.03400	-9.06100
H	-5.35200	-0.72300	-9.40600
H	-6.75700	-0.02500	-7.44200
H	-6.02900	-0.61300	-5.11500
H	-3.92200	-1.89300	-4.74200
H	-3.07300	-4.12500	-4.02700
H	-3.95700	-6.33500	-3.28600
H	-4.07100	-8.25400	-4.90400
H	-3.29300	-7.94500	-7.27100
H	-2.40500	-5.74500	-8.02100
H	1.48800	-3.59400	-7.22800
H	3.07600	-1.79500	-6.62600
C	7.12600	-1.24300	-10.04500
O	6.78900	-1.19900	-11.41700
H	8.14100	-0.79700	-9.96700
O	7.23700	-2.56600	-9.53700
H	5.82500	-1.42700	-11.51300
H	6.33600	-2.88600	-9.26200

Complex 1_a^{2+}⊂CB[8], local minimum **m'$_3$** BLYP-D3(BJ)/def2-SVP/SMD (water).

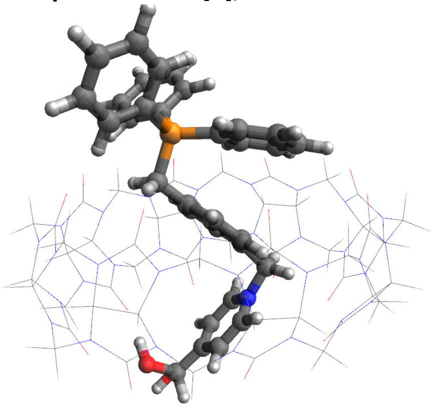

0 imaginary frequencies at BLYP-D3(BJ)/def2-SVP/SMD (water])
Total electronic energy BLYP-D3(BJ)/def2-QZVP/SMD (water): -6592.231333 hartree.
210

C	7.22200	0.96600	-0.12100
H	8.31600	0.86400	-0.26500
N	6.51700	0.69900	-1.38400
C	5.96700	1.85900	-1.92700
O	5.41400	1.96100	-3.02800
N	6.19900	2.89300	-1.02800
C	5.89700	4.28200	-1.31300
N	4.60800	4.75400	-0.84500
C	3.53300	4.99200	-1.69000
O	3.49100	4.76000	-2.90400
N	2.52600	5.58800	-0.93400
C	1.48800	6.38600	-1.56700
N	0.14000	5.91900	-1.32700
C	-0.61900	5.27300	-2.29200
O	-0.23800	4.97100	-3.43000
N	-1.89600	5.07000	-1.76800
C	-3.03500	4.89600	-2.66100
N	-3.91100	3.79400	-2.31900
C	-3.96300	2.60200	-3.02400
O	-3.17900	2.28000	-3.93300
N	-5.05400	1.87100	-2.56700
C	-5.67500	0.83100	-3.38200
N	-5.68700	-0.48500	-2.76600
C	-5.18400	-1.59800	-3.44100
O	-4.45600	-1.58500	-4.43900
N	-5.68300	-2.73400	-2.80900
C	-5.35800	-4.08500	-3.22700
N	-4.14000	-4.63700	-2.66400
C	-2.94000	-4.68800	-3.36200
O	-2.75000	-4.29000	-4.51700
N	-1.99900	-5.31300	-2.54400
C	-0.81700	-5.93500	-3.12300
N	0.43800	-5.53000	-2.52700
C	1.31300	-4.62200	-3.10900
O	1.11200	-3.97900	-4.14700
N	2.47800	-4.59800	-2.33700
C	3.73900	-4.18900	-2.93900
N	4.51700	-3.26800	-2.13000
C	4.94600	-2.04400	-2.64500
O	4.45400	-1.44600	-3.61000
N	6.05400	-1.64200	-1.91400
C	6.41300	-2.57800	-0.86000
H	7.47100	-2.88700	-0.97100
N	6.17100	-2.13100	0.51300
C	7.06800	-1.22300	1.21700
N	6.78900	0.19100	1.03100
C	5.89100	0.89600	1.82500
O	5.33500	0.46700	2.84300
N	5.76500	2.18000	1.29800
C	5.19700	3.27200	2.07700
N	4.08800	3.96500	1.43500
C	2.80900	4.00400	1.99500
O	2.36800	3.23500	2.85700
N	2.13300	5.08800	1.44600
C	0.84000	5.54800	1.91800
N	-0.28700	5.28000	1.02800
C	-1.43200	4.63200	1.49900
O	-1.50900	3.95100	2.52800
N	-2.47800	4.92500	0.62800
C	-3.87200	4.63800	0.91000
N	-4.46300	3.58900	0.08100
C	-5.23500	2.58700	0.67000
O	-5.22200	2.26200	1.86200
N	-6.06000	2.04600	-0.30600
C	-6.99100	0.96400	-0.03700
N	-6.55100	-0.35800	-0.44400

C	-5.83000	-1.22500	0.37000
O	-5.47100	-0.99500	1.53200
N	-5.62300	-2.40300	-0.34400
C	-5.21100	-3.63400	0.31700
N	-3.99500	-4.22800	-0.22200
C	-2.84400	-4.37700	0.54900
O	-2.60400	-3.80800	1.62400
N	-2.02400	-5.30700	-0.07600
C	-0.83300	-5.86300	0.53700
N	0.43000	-5.42600	-0.05500
C	1.47700	-4.99500	0.76700
O	1.36600	-4.58600	1.92900
N	2.66700	-5.14600	0.06200
C	3.98800	-5.02900	0.65600
N	4.74000	-3.85000	0.25600
C	5.27800	-2.96300	1.18300
O	5.02900	-2.94400	2.39400
C	5.38400	-3.74000	-1.05000
H	5.84600	-4.70700	-1.33100
C	2.44200	-5.63100	-1.28800
H	3.15300	-6.44600	-1.52900
C	0.94900	-6.06400	-1.27800
H	0.78900	-7.16000	-1.23300
C	-2.60600	-5.82000	-1.30800
H	-2.59400	-6.92800	-1.30400
C	-4.03700	-5.19700	-1.32700
H	-4.85300	-5.92600	-1.15400
C	-6.40700	-2.40200	-1.60100
H	-7.30300	-3.04400	-1.49300
C	-6.71400	-0.88900	-1.78100
H	-7.72800	-0.66800	-2.16800
C	-5.85400	2.65400	-1.60600
H	-6.82500	2.92600	-2.06500
C	-4.93000	3.86900	-1.28900
H	-5.44200	4.85100	-1.32300
C	-2.05700	5.76300	-0.47900
H	-2.74700	6.62200	-0.59600
C	-0.59800	6.16700	-0.10200
H	-0.48500	7.22500	0.20700
C	2.92900	5.80200	0.45700
H	2.94700	6.88500	0.68700
C	4.32500	5.10300	0.53800
H	5.13200	5.74300	0.94600
C	6.74900	2.41300	0.22400
H	7.55400	3.08600	0.58000
C	6.81700	-0.44900	-2.22400
H	5.91700	4.41700	-2.40700
H	6.68700	4.90200	-0.84700
H	1.66300	6.36000	-2.65600
H	1.56600	7.43000	-1.20000
H	-3.64300	5.82400	-2.66200
H	-2.63800	4.71600	-3.67400
H	-5.11000	0.75000	-4.32700
H	-6.71700	1.14100	-3.60500
H	-5.23800	-4.08400	-4.32400
H	-6.20600	-4.73800	-2.94700
H	-0.79100	-5.65900	-4.19000
H	-0.90100	-7.03700	-3.02600
H	3.50300	-3.68700	-3.89200
H	4.35400	-5.09400	-3.13800
H	8.10500	-1.41100	0.87800
H	6.98700	-1.44700	2.29500
H	5.99400	4.01600	2.28800
H	4.83600	2.84700	3.02900
H	0.90600	6.64400	2.08600
H	0.63100	5.04100	2.87600
H	-4.45000	5.57600	0.77000
H	-3.94900	4.31200	1.96000
H	-7.94000	1.17800	-0.56400
H	-7.16900	0.94900	1.05200
H	-6.03000	-4.37900	0.23400
H	-5.03700	-3.40100	1.38100
H	-0.82200	-5.55500	1.59600
H	-0.89600	-6.96900	0.47200
H	4.56400	-5.93600	0.37900
H	3.86900	-4.98800	1.75100
H	6.59000	-0.16800	-3.26700
H	7.89400	-0.69100	-2.12900
C	0.01700	0.74400	-4.87400
C	0.39700	1.95300	-4.26000
C	1.25100	1.93200	-3.15900
C	0.55300	-0.46200	-4.37300
C	1.40100	-0.43500	-3.27200
N	1.72500	0.74900	-2.67500
C	2.38200	0.71000	-1.32100
C	1.29100	0.40300	-0.30900
C	0.38100	1.41500	0.06900
C	-0.75500	1.10000	0.83100
C	-1.00400	-0.23300	1.22400
C	-2.19800	-0.57300	2.08800
P	-1.77400	-0.46100	3.89700
C	0.00000	-0.77700	4.03800
C	0.89500	0.27100	3.70900
C	2.26700	0.00300	3.62000
C	2.75100	-1.29800	3.85100
C	1.86000	-2.34100	4.16200
C	0.48000	-2.08900	4.24800
C	-2.17600	1.17900	4.58000
C	-1.31500	1.83500	5.48900
C	-1.69800	3.07300	6.03300
C	-2.93600	3.64700	5.68900
C	-3.80200	2.97600	4.80600
C	-3.42800	1.74100	4.25100
C	-2.74000	-1.68900	4.82600
C	-2.31900	-2.05000	6.12800
C	-3.08700	-2.95600	6.87700

C	-4.27600	-3.48900	6.34000
C	-4.70200	-3.10900	5.05400
C	-3.93900	-2.20700	4.28900
C	-0.07600	-1.24000	0.87300
C	1.06100	-0.92600	0.11100
H	0.01800	2.91700	-4.61200
H	1.56800	2.84200	-2.63700
H	0.29500	-1.43300	-4.81600
H	1.81700	-1.34200	-2.82200
H	2.85700	1.69200	-1.15600
H	3.15100	-0.07700	-1.34800
H	0.54800	2.44700	-0.26800
H	-1.45800	1.88700	1.12900
H	-2.50600	-1.62400	1.93100
H	-3.06200	0.08900	1.90100
H	0.52900	1.28200	3.49500
H	2.96000	0.80800	3.35300
H	3.82300	-1.50200	3.75700
H	2.23700	-3.36100	4.31300
H	-0.21700	-2.91100	4.45300
H	-0.35400	1.39000	5.77400
H	-1.02300	3.58900	6.73000
H	-3.22800	4.61900	6.11300
H	-4.77100	3.41600	4.53100
H	-4.11100	1.23400	3.56100
H	-1.39700	-1.62800	6.55100
H	-2.75800	-3.24600	7.88400
H	-4.87400	-4.19800	6.92900
H	-5.63500	-3.51300	4.63700
H	-4.29500	-1.90500	3.29700
H	-0.24600	-2.27300	1.20300
H	1.76500	-1.71900	-0.17800
C	-1.01600	0.69100	-5.99800
O	-2.25300	0.21600	-5.49700
H	-0.69300	-0.06700	-6.74600
O	-1.10900	1.98000	-6.57600
H	-2.58800	0.87400	-4.82800
H	-1.75200	1.91200	-7.31200

Complex 1_a^{2+}⊂CB[8], local minimum m'_4 BLYP-D3(BJ)/def2-SVP/SMD (water).

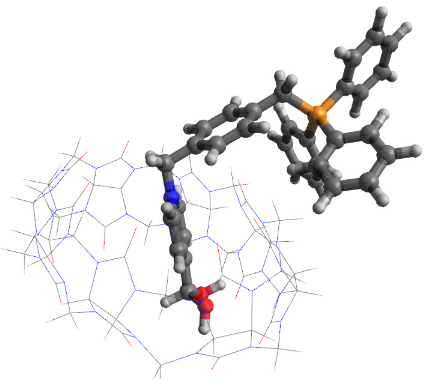

0 imaginary frequencies at BLYP-D3(BJ)/def2-SVP/SMD (water])
Total electronic energy BLYP-D3(BJ)/def2-QZVP/SMD (water): -6592.247783hartree.
210

C	5.56800	0.25400	0.66600
H	6.55900	0.18100	1.15800
N	5.66800	-0.14200	-0.72300
C	5.05400	0.76800	-1.57700
O	5.01900	0.69700	-2.81000
N	4.50800	1.79600	-0.79400
C	4.32700	3.11400	-1.40000
N	3.19800	3.86200	-0.87300
C	2.22500	4.40600	-1.71500
O	1.95700	4.01900	-2.85900
N	1.64600	5.48900	-1.06300
C	0.80600	6.45700	-1.75400
N	-0.61300	6.35700	-1.46300
C	-1.51900	5.84200	-2.37200
O	-1.24000	5.35200	-3.48100
N	-2.79400	6.00600	-1.85600
C	-3.99000	5.57900	-2.57200
N	-4.39700	4.19800	-2.36600
C	-4.17700	3.19200	-3.30200
O	-3.60700	3.31700	-4.39100
N	-4.78900	2.03200	-2.82900
C	-5.06000	0.88700	-3.67900
N	-4.46900	-0.36900	-3.22200
C	-3.70000	-1.17000	-4.07800
O	-3.18100	-0.80200	-5.13500
N	-3.64200	-2.45300	-3.53400
C	-3.26400	-3.60300	-4.35500
N	-2.31900	-4.51100	-3.73500
C	-1.02000	-4.70300	-4.20300
O	-0.44500	-4.01600	-5.05400
N	-0.50100	-5.82900	-3.56400
C	0.70200	-6.49600	-4.03400
N	1.79100	-6.52300	-3.06900
C	2.99800	-5.87700	-3.32400
O	3.20000	-5.02500	-4.19700
N	3.95100	-6.39400	-2.45000
C	5.28500	-5.83100	-2.34400
N	5.39400	-4.65000	-1.49700
C	5.65600	-3.39000	-2.02900
O	5.62000	-3.07800	-3.22500
N	6.01400	-2.55400	-0.97700

C	5.98000	-3.22700	0.31200
H	6.93100	-3.06400	0.85700
N	4.84000	-2.87800	1.17400
C	4.88900	-1.74100	2.08100
N	4.55800	-0.46000	1.46600
C	3.71700	0.44200	2.12400
O	2.90700	0.16700	3.01900
N	3.96700	1.71400	1.61400
C	3.60700	2.93400	2.31300
N	2.76200	3.83300	1.54400
C	1.67200	4.46600	2.13400
O	1.09000	4.09700	3.16200
N	1.39800	5.61300	1.39900
C	0.31800	6.53000	1.72200
N	-0.92300	6.32000	0.99500
C	-1.90000	5.43600	1.43500
O	-1.84000	4.72400	2.44500
N	-2.98000	5.54200	0.57200
C	-4.24700	4.90000	0.85700
N	-4.51300	3.71600	0.06000
C	-4.25600	2.43200	0.51400
O	-3.70400	2.13200	1.58000
N	-4.77600	1.53700	-0.42100
C	-5.20200	0.20700	-0.02100
N	-4.62900	-0.86800	-0.81000
C	-4.06300	-1.98200	-0.19900
O	-3.72200	-2.06400	0.98600
N	-3.96800	-2.99600	-1.14900
C	-3.82600	-4.39000	-0.77100
N	-2.66400	-5.04700	-1.35000
C	-1.87700	-5.88600	-0.55600
O	-1.80000	-5.85300	0.67800
N	-1.23100	-6.78400	-1.39700
C	-0.34300	-7.83200	-0.91900
N	1.07300	-7.50600	-0.90800
C	1.69800	-6.90500	0.18100
O	1.17600	-6.64700	1.27100
N	3.03200	-6.69400	-0.16200
C	4.03100	-6.41900	0.86300
N	4.61000	-5.09000	0.81200
C	4.07700	-4.00400	1.49500
O	3.12200	-4.03700	2.28000
C	5.70800	-4.72100	-0.06600
H	6.57200	-5.39500	0.09800
C	3.36000	-7.28800	-1.46900
H	3.99500	-8.18500	-1.33000
C	1.94700	-7.58900	-2.06600
H	1.85700	-8.58300	-2.54600
C	-1.52900	-6.54300	-2.79800
H	-1.78500	-7.49500	-3.30300
C	-2.69400	-5.50900	-2.74600
H	-3.68800	-5.93500	-2.98700
C	-4.57500	-2.61000	-2.41700
H	-5.37500	-3.32900	-2.68600
C	-5.09300	-1.16000	-2.16200
H	-6.19600	-1.06300	-2.21800
C	-5.43000	2.23000	-1.53500
H	-6.49900	1.94400	-1.58400
C	-5.19200	3.74600	-1.22700
H	-6.12000	4.34800	-1.15000
C	-2.76900	6.52700	-0.49100
H	-3.48600	7.36400	-0.39000
C	-1.26600	6.93400	-0.28000
H	-1.09000	8.02700	-0.24100
C	2.26200	5.75900	0.23600
H	2.72600	6.76500	0.23100
C	3.27900	4.58100	0.39600
H	4.31900	4.91000	0.58500
C	4.99800	1.69600	0.59200
H	5.75100	2.48500	0.79100
C	6.53500	-1.21600	-1.17600
H	4.16300	2.96300	-2.48000
H	5.25000	3.71700	-1.24900
H	0.92800	6.29900	-2.83900
H	1.15700	7.47200	-1.48400
H	-4.82000	6.24300	-2.26500
H	-3.79800	5.70400	-3.65100
H	-4.64300	1.10800	-4.67700
H	-6.16000	0.75000	-3.75700
H	-2.80500	-3.21000	-5.27800
H	-4.18100	-4.17500	-4.60900
H	1.06000	-5.95000	-4.92400
H	0.44800	-7.54000	-4.31500
H	5.61000	-5.54000	-3.35800
H	5.95700	-6.61800	-1.95300
H	5.90900	-1.67700	2.51900
H	4.15500	-1.92100	2.88500
H	4.54300	3.46500	2.59300
H	3.05500	2.65800	3.22700
H	0.66100	7.56200	1.52100
H	0.10500	6.41700	2.79900
H	-5.06800	5.62300	0.68400
H	-4.23500	4.60100	1.91900
H	-6.30600	0.16200	-0.09400
H	-4.89400	0.04000	1.02200
H	-4.73900	-4.93700	-1.08600
H	-3.72300	-4.43900	0.32500
H	-0.63800	-8.07000	0.11700
H	-0.48500	-8.72300	-1.55800
H	4.85300	-7.15500	0.77300
H	3.53700	-6.53500	1.84300
H	6.70100	-1.08200	-2.25900
H	7.50100	-1.12900	-0.63800
C	-0.70200	1.10600	-0.80500
C	-0.48800	1.93900	0.31100
C	-0.36900	1.38000	1.57900

C	-0.83500	-0.28100	-0.59600
C	-0.72600	-0.79600	0.69200
N	-0.47300	0.03100	1.74700
C	-0.37600	-0.55100	3.12800
C	-1.75300	-0.79900	3.71300
C	-2.54100	0.27900	4.17200
C	-3.82100	0.04900	4.69700
C	-4.35100	-1.26200	4.75600
C	-5.72100	-1.52400	5.34200
P	-7.19000	-1.30900	4.22600
C	-7.00300	-2.18700	2.64900
C	-6.45400	-3.49100	2.62900
C	-6.43500	-4.21300	1.42600
C	-6.96500	-3.64800	0.25000
C	-7.51600	-2.35500	0.27400
C	-7.54000	-1.62100	1.47200
C	-7.54700	0.44400	3.89100
C	-8.79500	0.99100	4.27000
C	-9.07900	2.33800	3.98800
C	-8.12500	3.14000	3.33400
C	-6.88400	2.59400	2.95400
C	-6.59200	1.24700	3.22200
C	-8.61400	-2.02900	5.10300
C	-9.56700	-2.78700	4.38500
C	-10.68600	-3.30900	5.05600
C	-10.85600	-3.08000	6.43400
C	-9.90600	-2.32300	7.14700
C	-8.78500	-1.79400	6.48700
C	-3.55900	-2.33600	4.29600
C	-2.27100	-2.10700	3.78400
H	-0.40900	3.02300	0.19700
H	-0.17700	1.98000	2.47600
H	-1.03200	-0.96200	-1.43200
H	-0.82600	-1.86400	0.91900
H	0.20200	0.17200	3.72600
H	0.20400	-1.48500	3.04100
H	-2.15200	1.30500	4.11700
H	-4.41600	0.89100	5.07400
H	-5.93300	-0.84700	6.19200
H	-5.81000	-2.56600	5.70300
H	-6.05700	-3.94400	3.54600
H	-6.00200	-5.22300	1.40600
H	-6.94800	-4.21800	-0.68900
H	-7.92700	-1.91100	-0.64300
H	-7.98000	-0.61600	1.49100
H	-9.54300	0.37000	4.77800
H	-10.05000	2.76000	4.28100
H	-8.34900	4.19400	3.11700
H	-6.13800	3.21900	2.45100
H	-5.62700	0.83200	2.91600
H	-9.43800	-2.96800	3.31000
H	-11.42700	-3.89900	4.49800
H	-11.73200	-3.49200	6.95500
H	-10.03800	-2.14200	8.22300
H	-8.05600	-1.19500	7.04900
H	-3.95100	-3.36000	4.34700
H	-1.66700	-2.95300	3.42600
C	-0.75200	1.70600	-2.20900
O	-1.37000	2.96700	-2.09100
H	0.29500	1.81200	-2.57300
O	-1.38400	0.86000	-3.15400
H	-1.19100	3.51200	-2.89400
H	-2.31200	0.72600	-2.86000

NMR spectra:

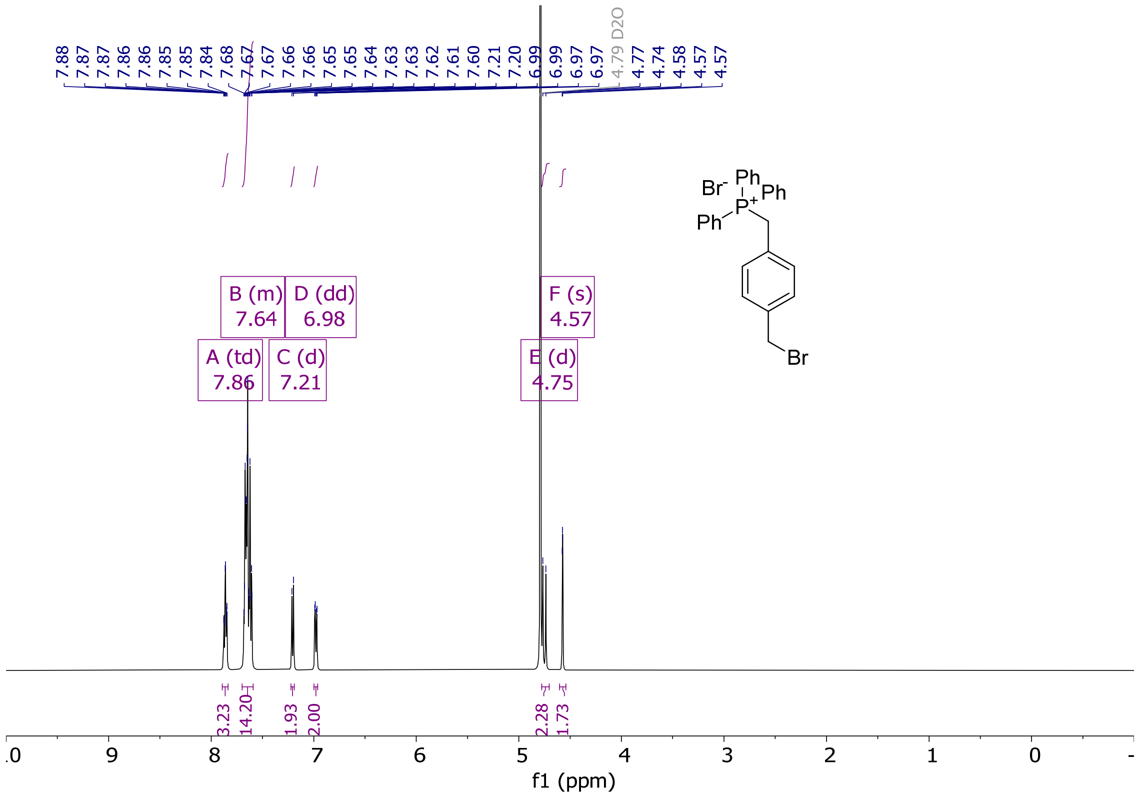

*Figure S11. ^{1}H NMR (500 MHz, D_2O) spectrum of **P1**·Br.*

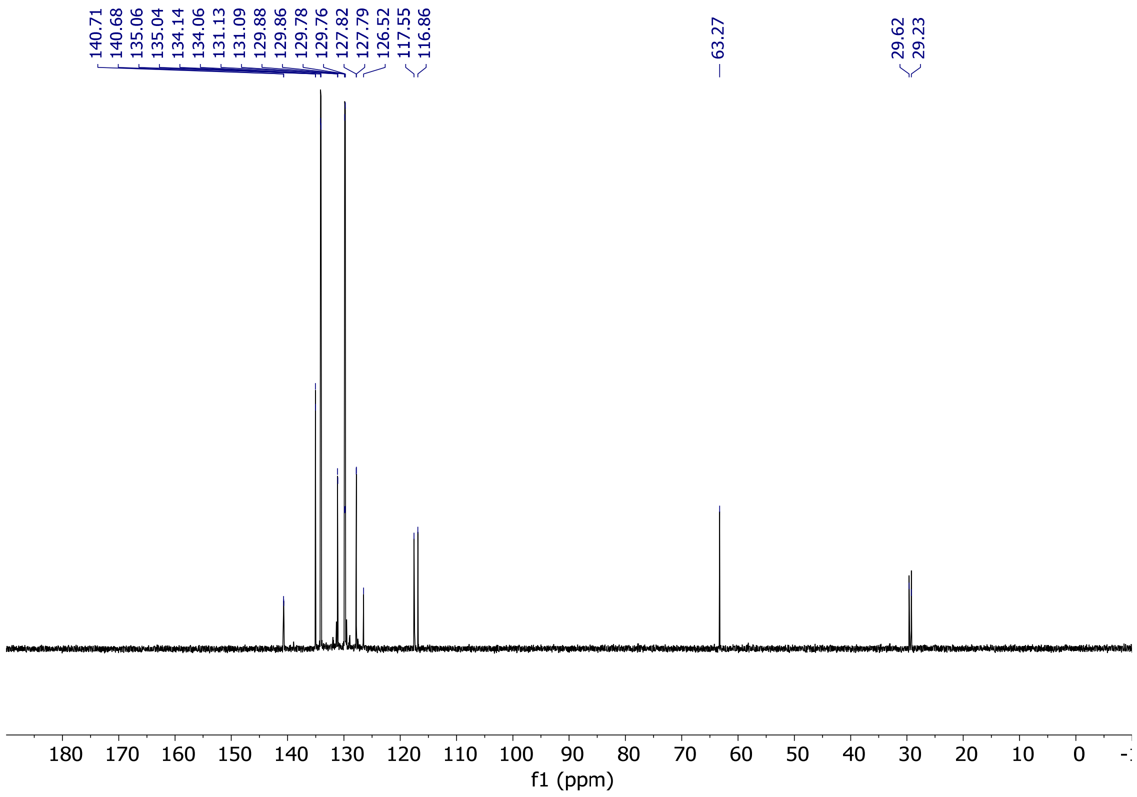

*Figure S12 $^{13}C\{^{1}H\}$ NMR (125 MHz, D_2O) spectrum of **P1**·Br.*

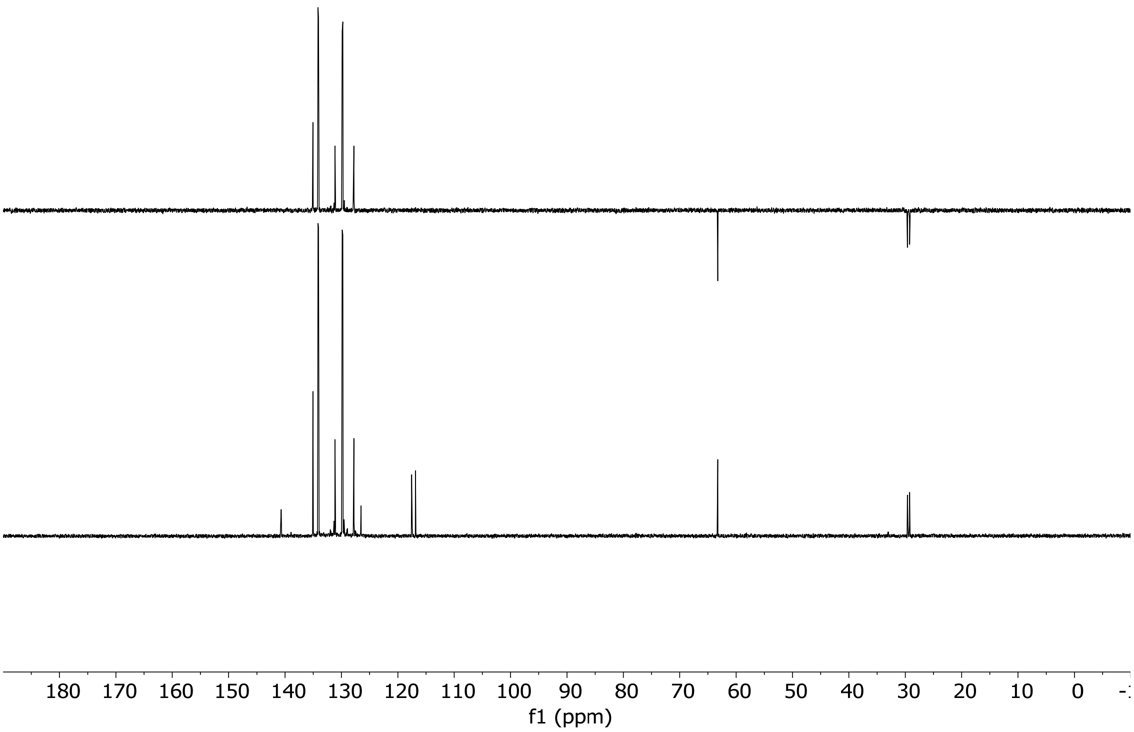

*Figure S13 $^{13}C\{^1H\}$ and DEPT 135$\{^1H\}$ NMR (125 MHz, D_2O) spectrum of **P1**·Br.*

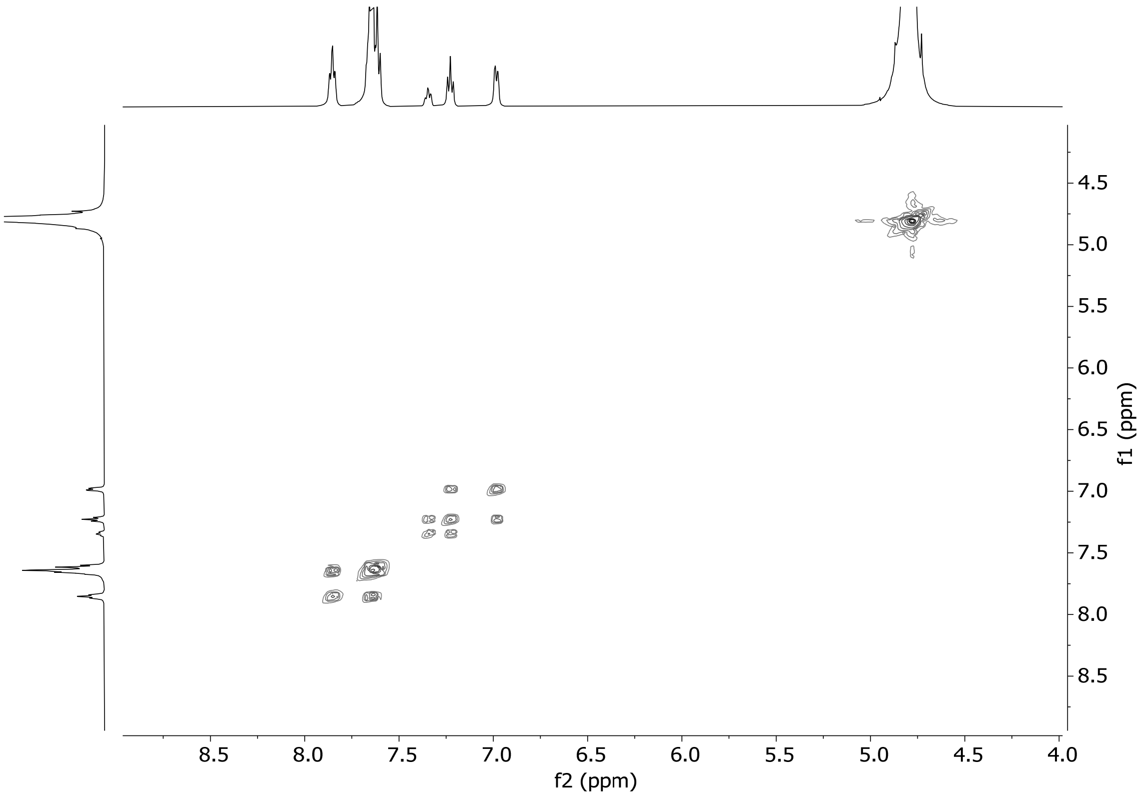

*Figure S14 COSY (500 MHz, D_2O) spectrum of **P1**·Br.*

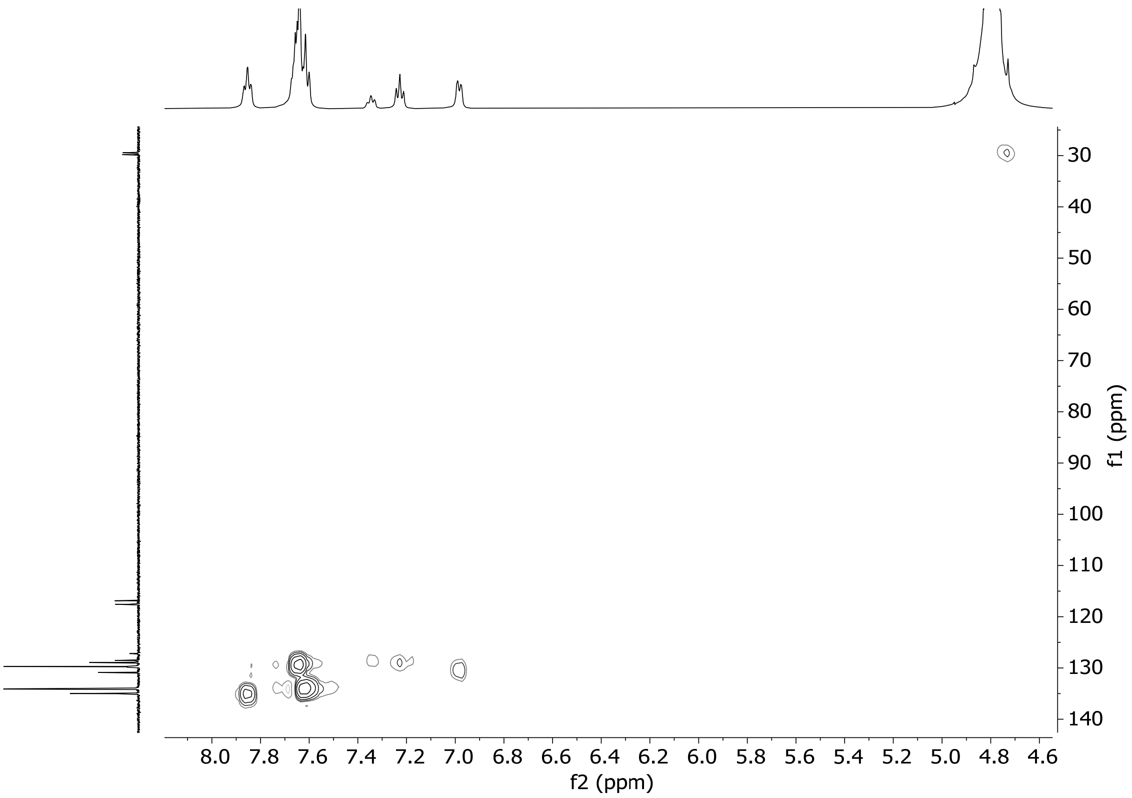

*Figure S15 HSQC (500 MHz, D_2O) spectrum of **P1**·Br.*

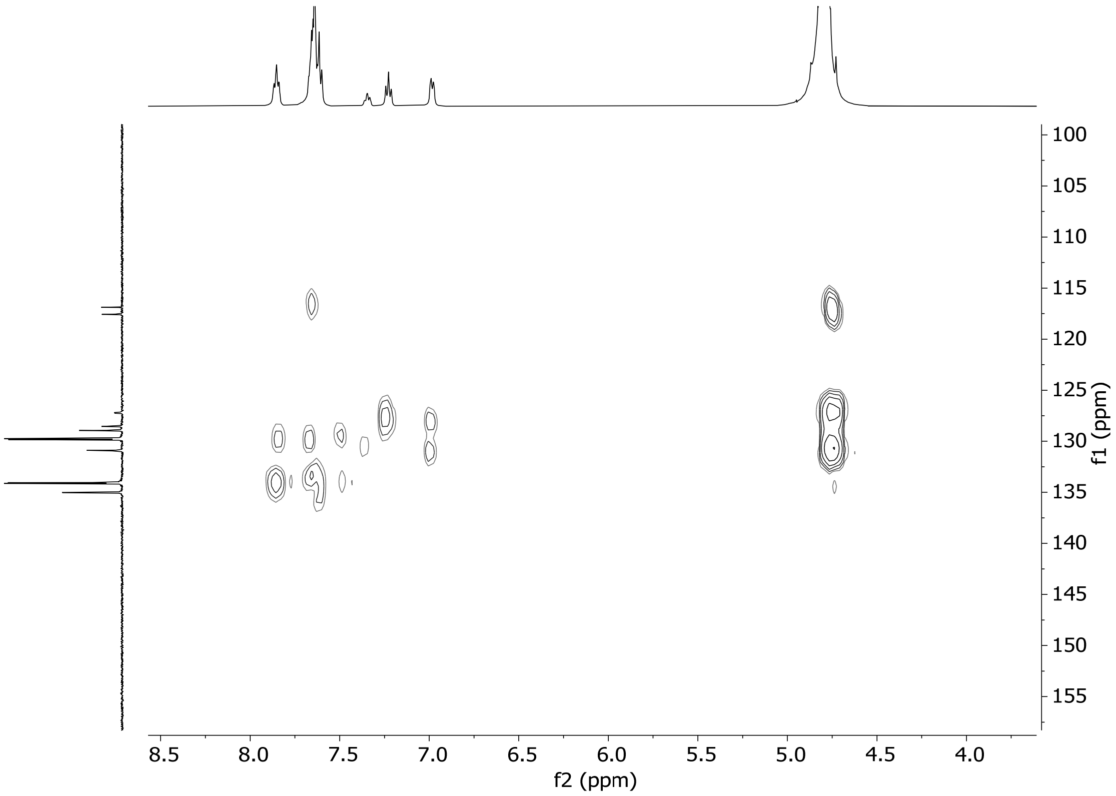

*Figure S16 HMBC (500 MHz, D_2O) spectrum of **P1**·Br.*

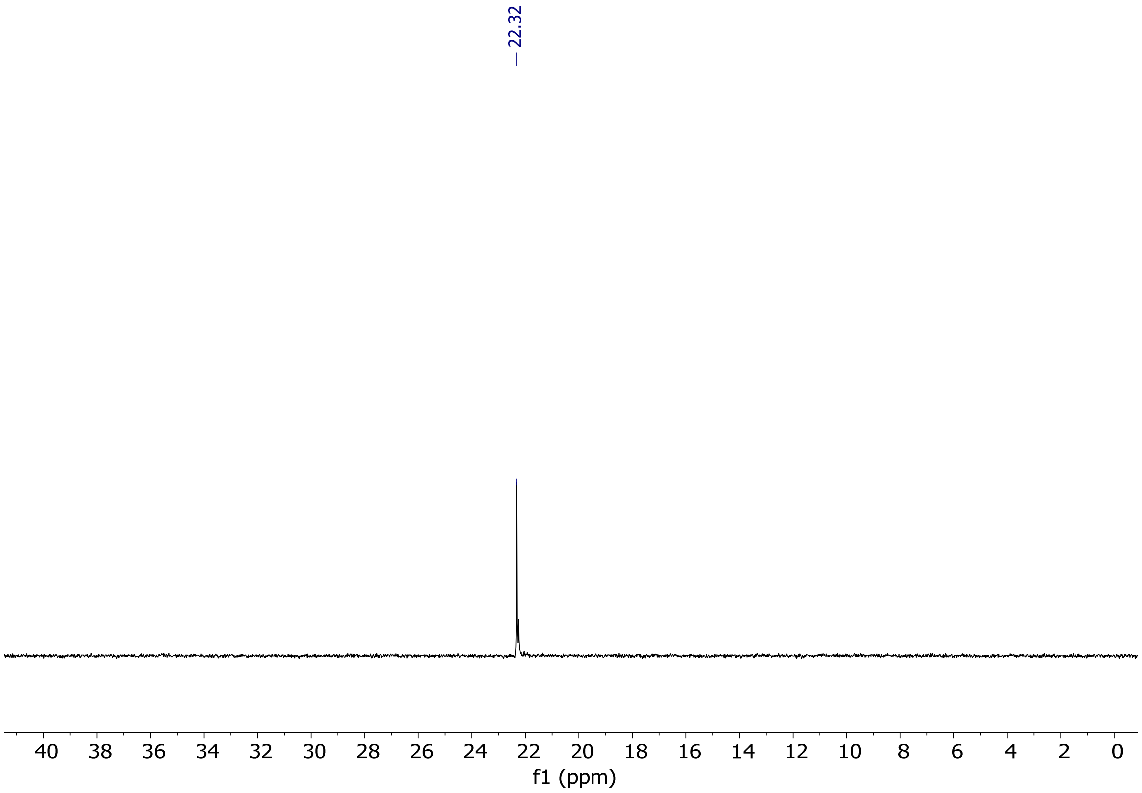

*Figure S17 $^{31}P\{^1H\}$ (160 MHz, D_2O) spectrum of **P1**·Br.*

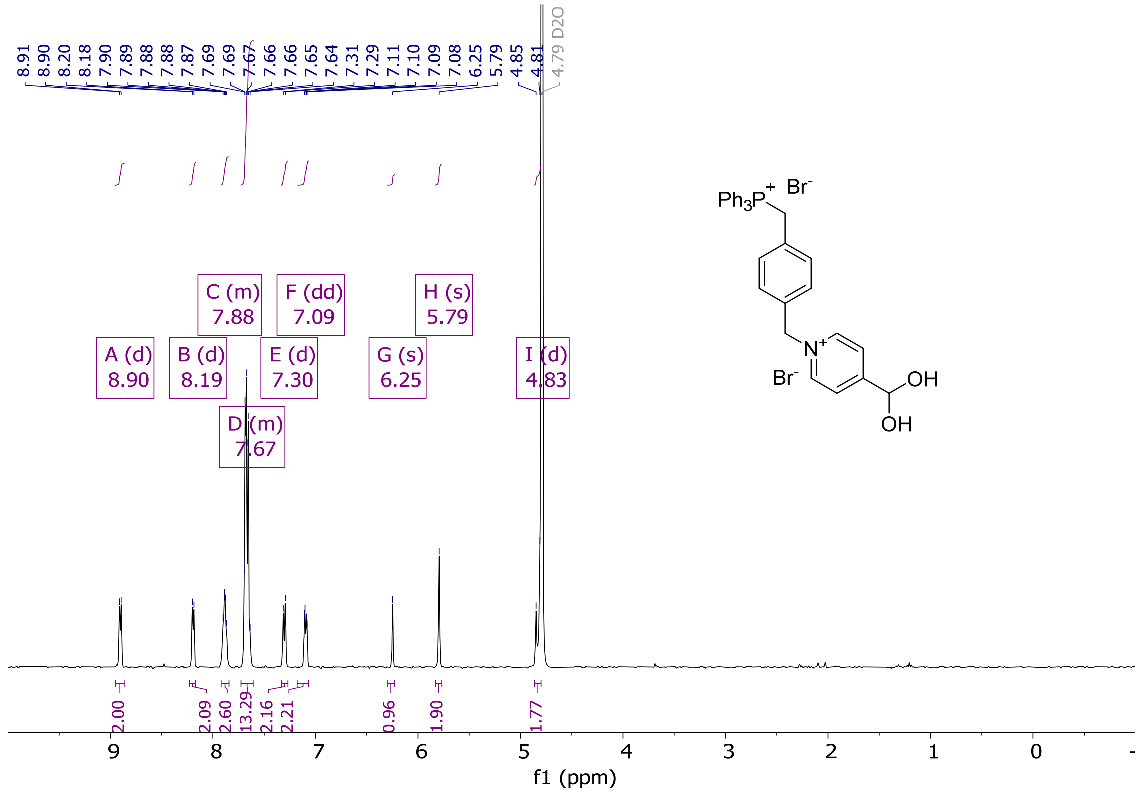

*Figure S18 ^1H NMR (500 MHz, D_2O) spectrum of **1a**·2Br.*

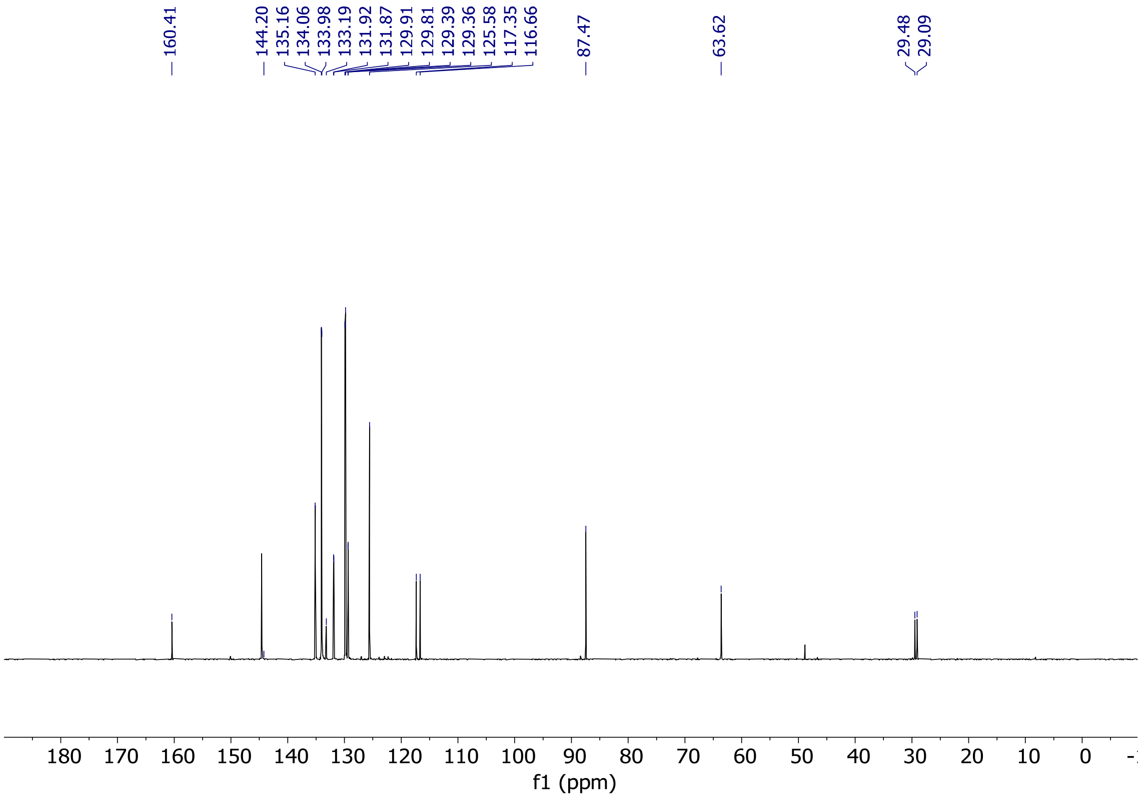

*Figure S19 $^{13}C\{^{1}H\}$ NMR (125 MHz, D_2O) spectrum of **1a**·2Br.*

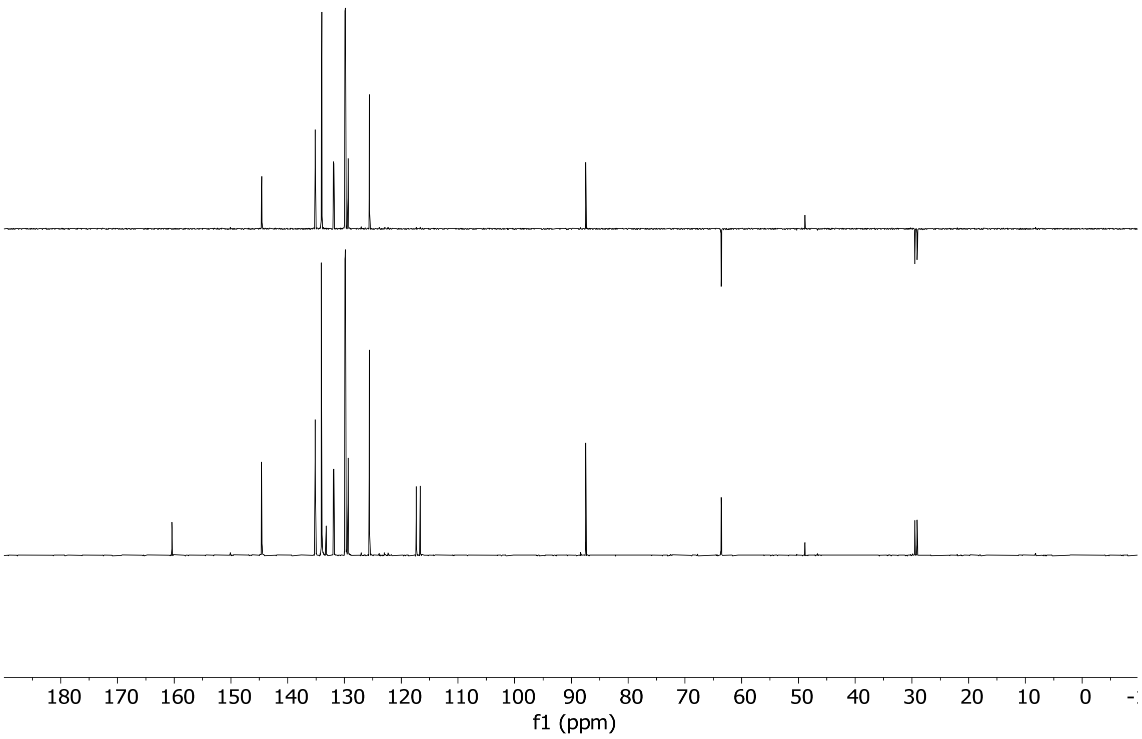

*Figure S20 $^{13}C\{^{1}H\}$ and DEPT 135$\{^{1}H\}$ NMR (125 MHz, D_2O) spectrum of **1a**·2Br.*

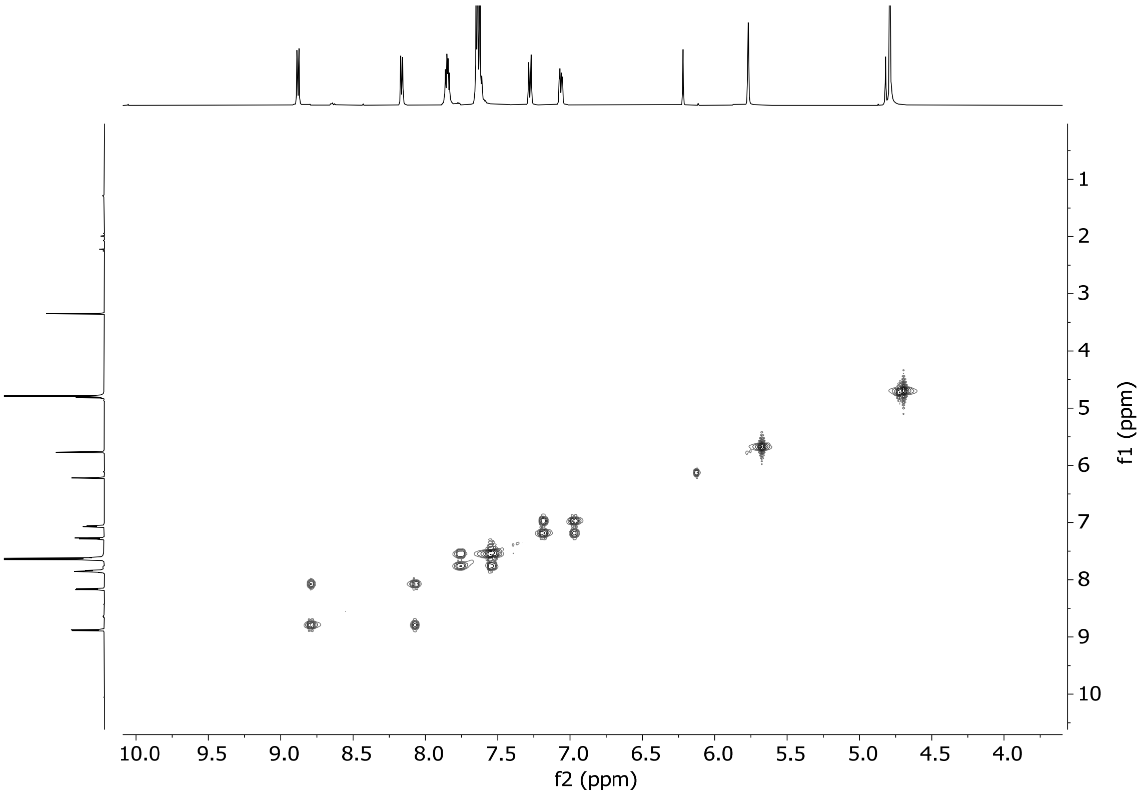

*Figure S21 COSY (500 MHz, D_2O) spectrum of **1a**·2Br.*

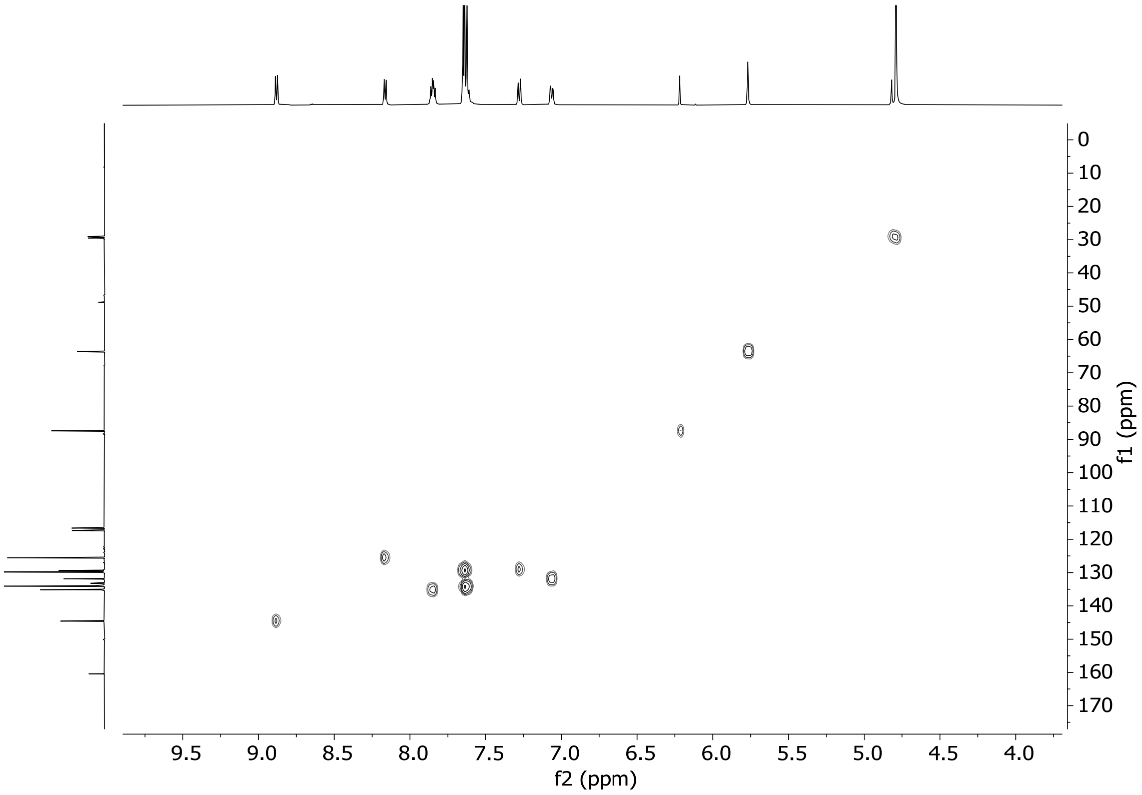

*Figure S22 HSQC (500 MHz, D_2O) spectrum of **1a**·2Br.*

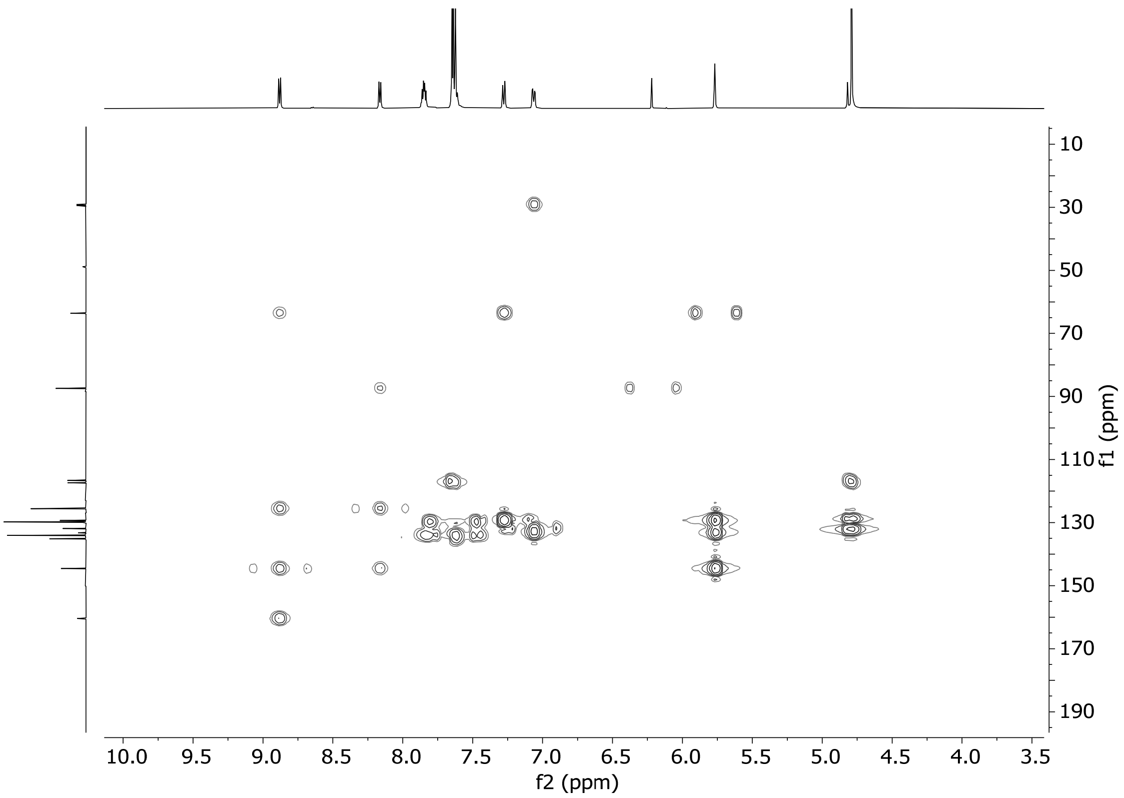

*Figure S23 HMBC (500 MHz, D_2O) spectrum of **1α**·2Br.*

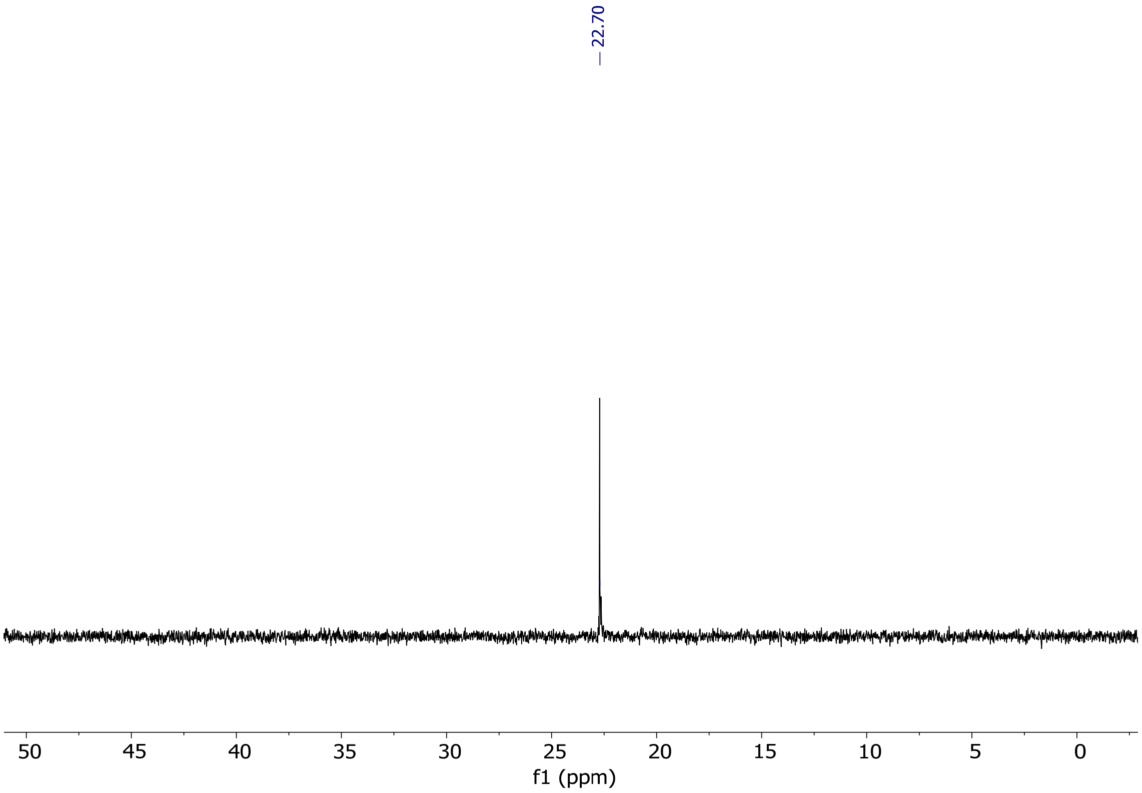

*Figure S24 ^{31}P (500 MHz, D_2O) spectrum of **1α**·2Br.*

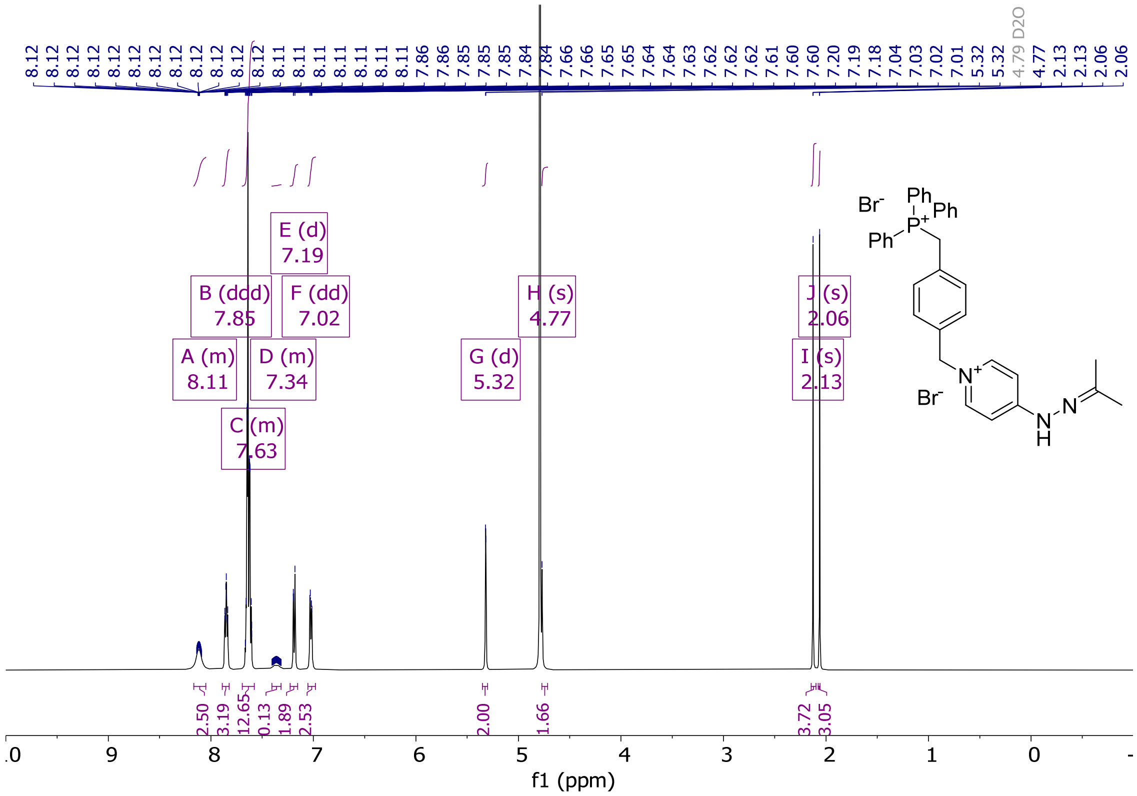

*Figure S25 ¹H NMR (500 MHz, D_2O) spectrum of **1b**·2Br.*

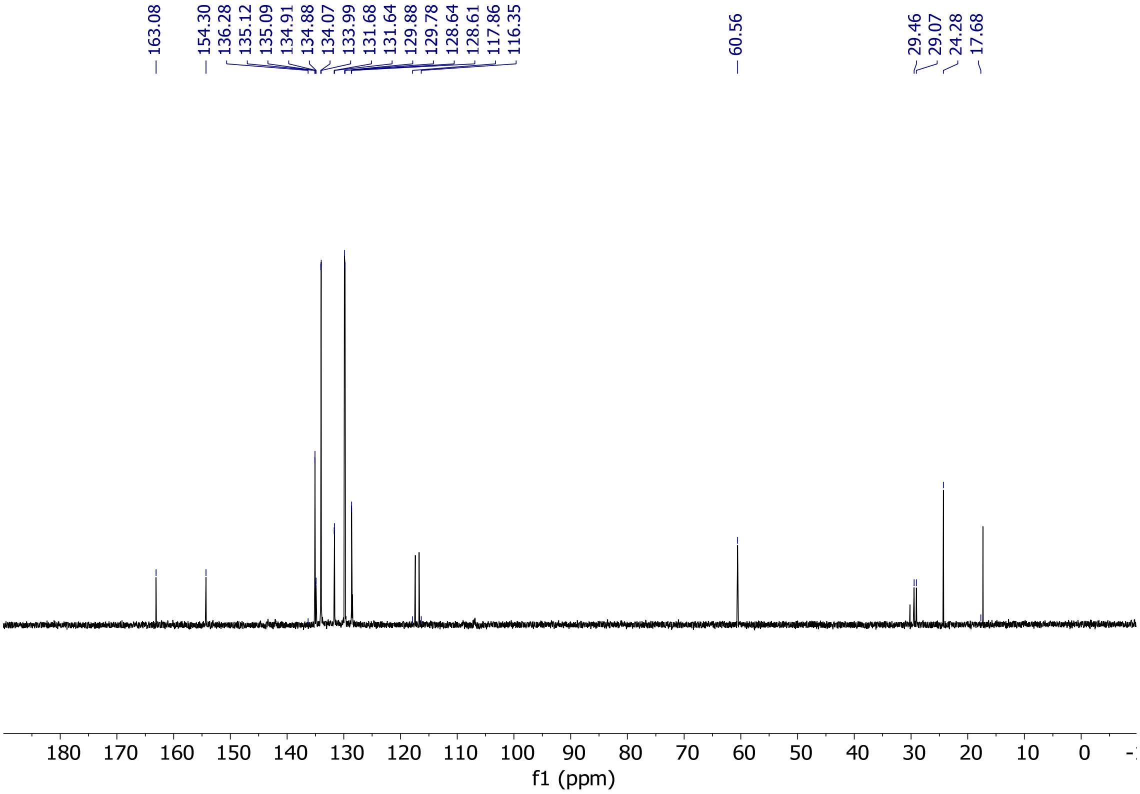

*Figure S26 ¹³C{¹H} NMR (125 MHz, D_2O) spectrum of **1b**·2Br.*

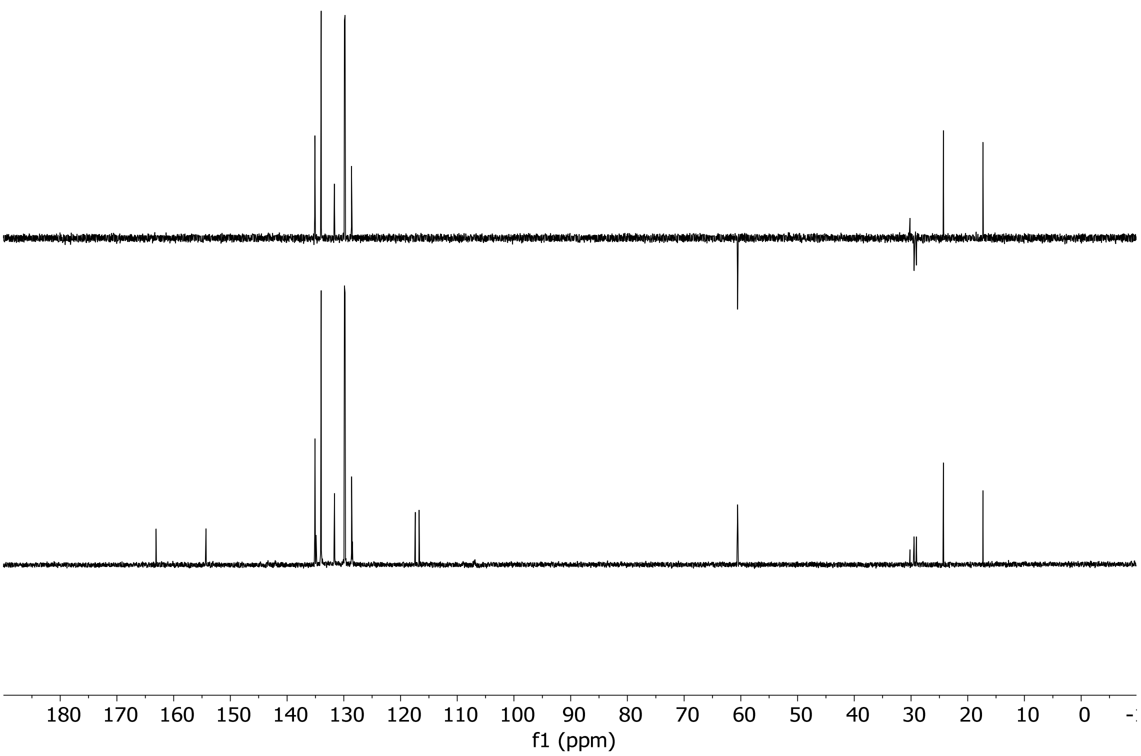

*Figure S27 $^{13}C\{^1H\}$ and DEPT 135$\{^1H\}$ NMR (125 MHz, D_2O) spectrum of **1b**·2Br.*

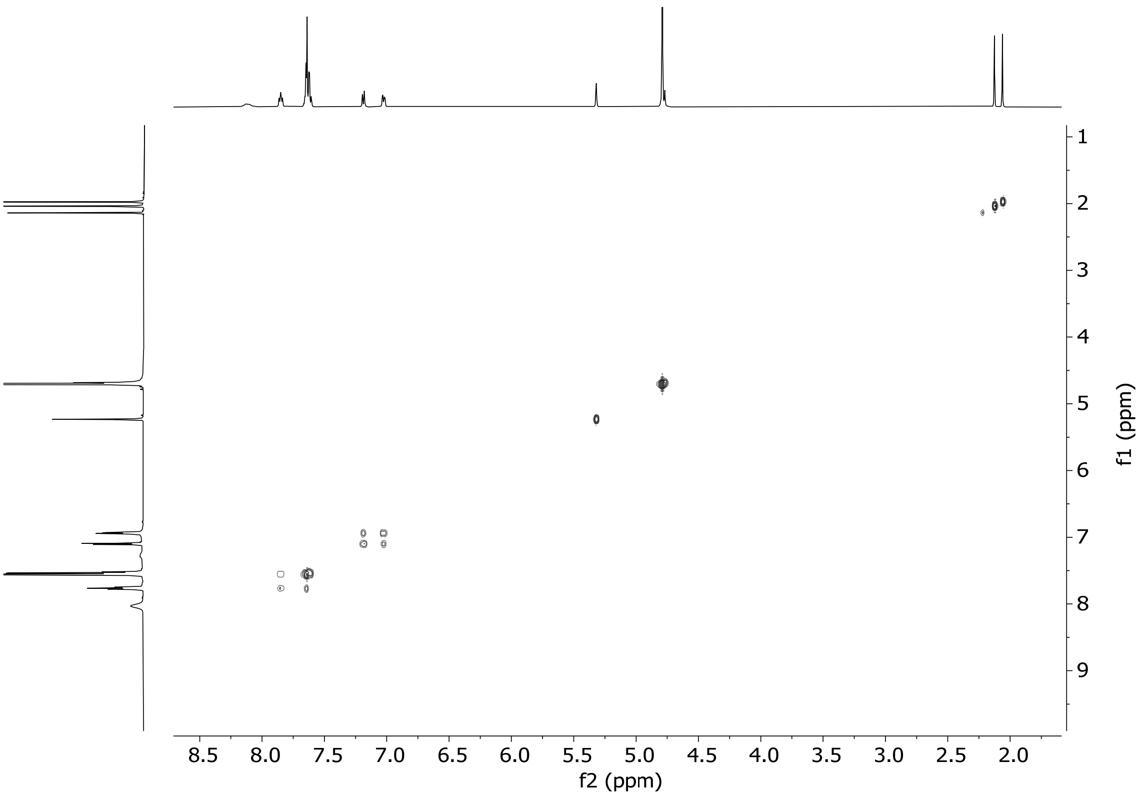

*Figure S28 COSY (500 MHz, D_2O) spectrum of **1b**·2Br.*

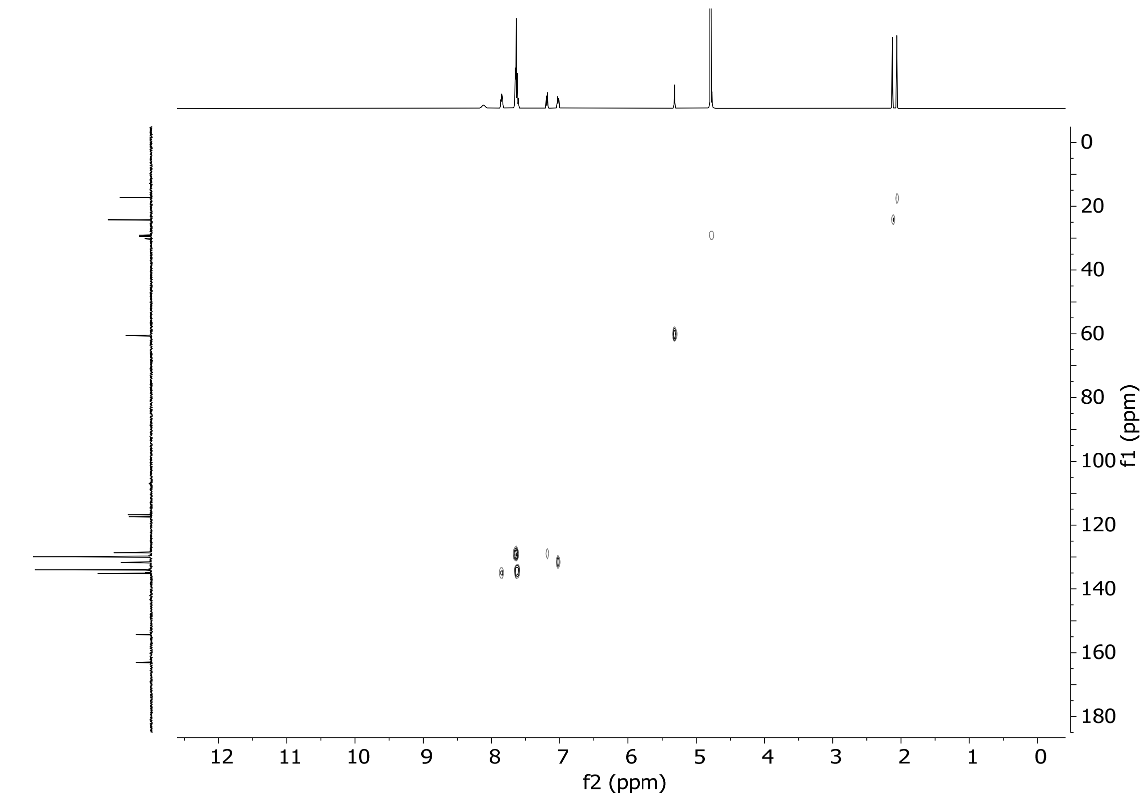

*Figure S29 HSQC (500 MHz, D_2O) spectrum of **1b**·2Br.*

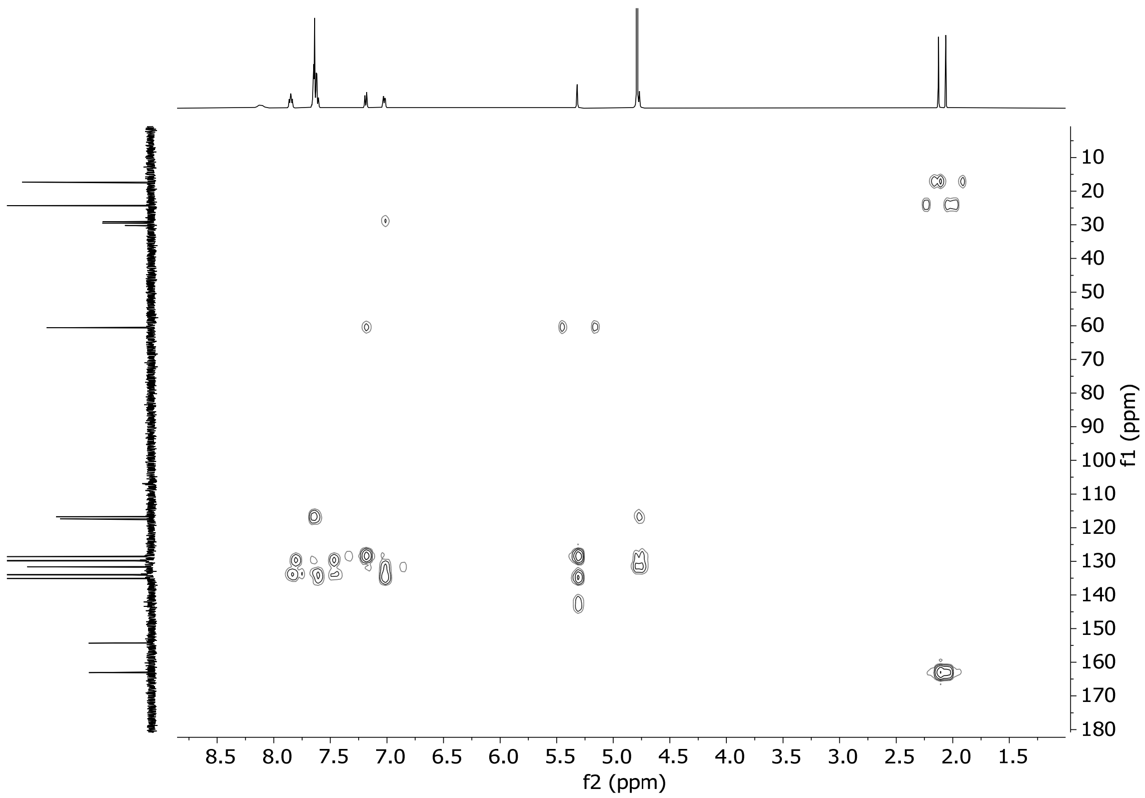

*Figure S30 HMBC (500 MHz, D_2O) spectrum of **1b**·2Br.*

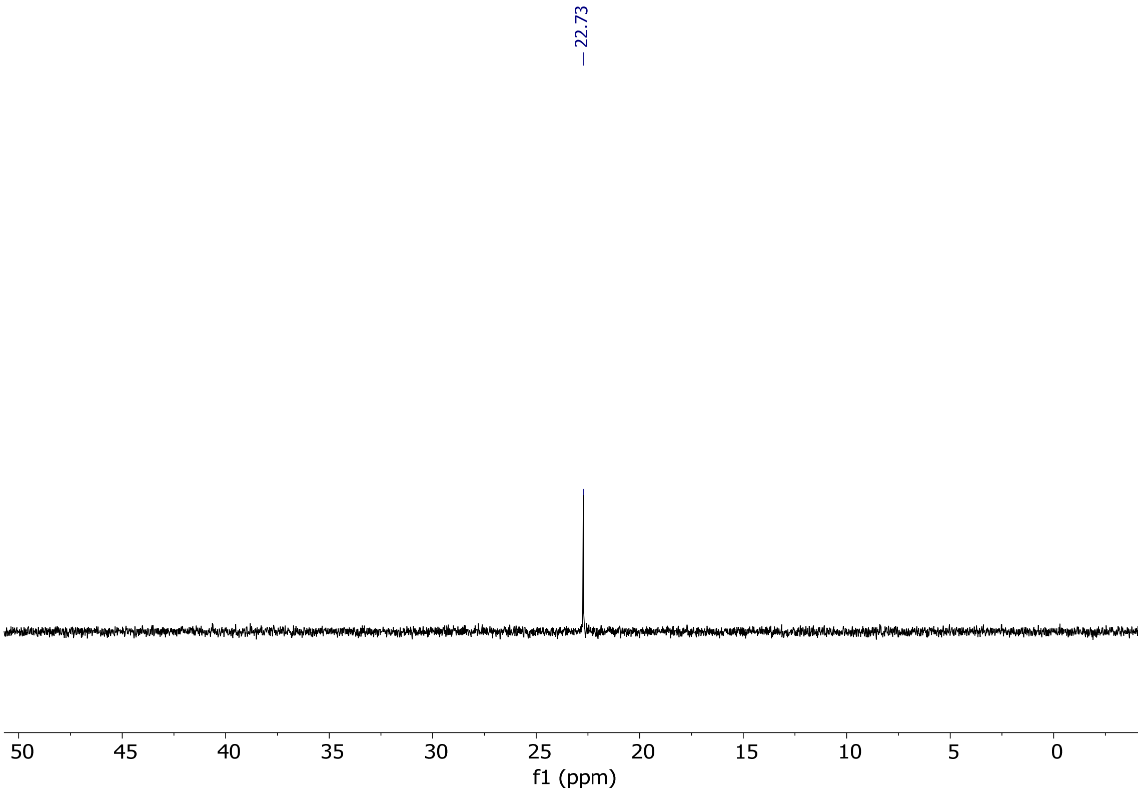

*Figure S31 $^{31}P\{^{1}H\}$ NMR (160 MHz, D_2O) spectrum of **1b**·2Br.*

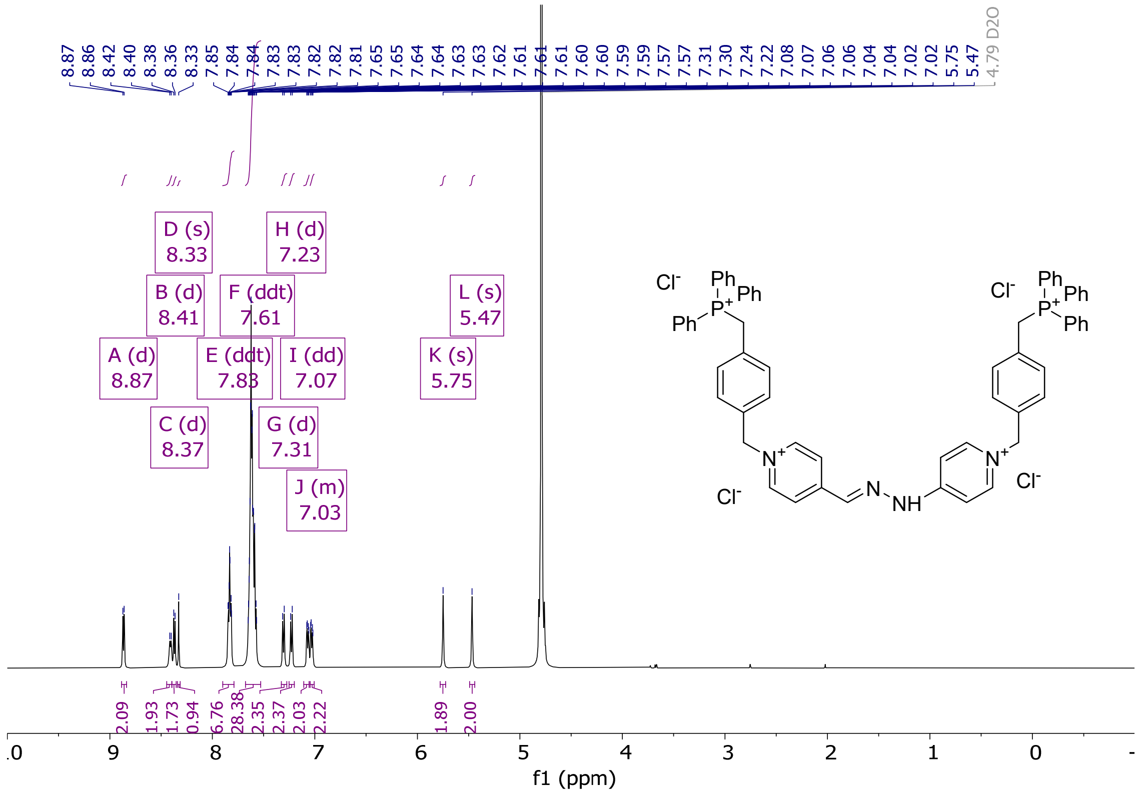

*Figure S32 ^{1}H NMR (500 MHz, D_2O) spectrum of **2**·4Cl.*

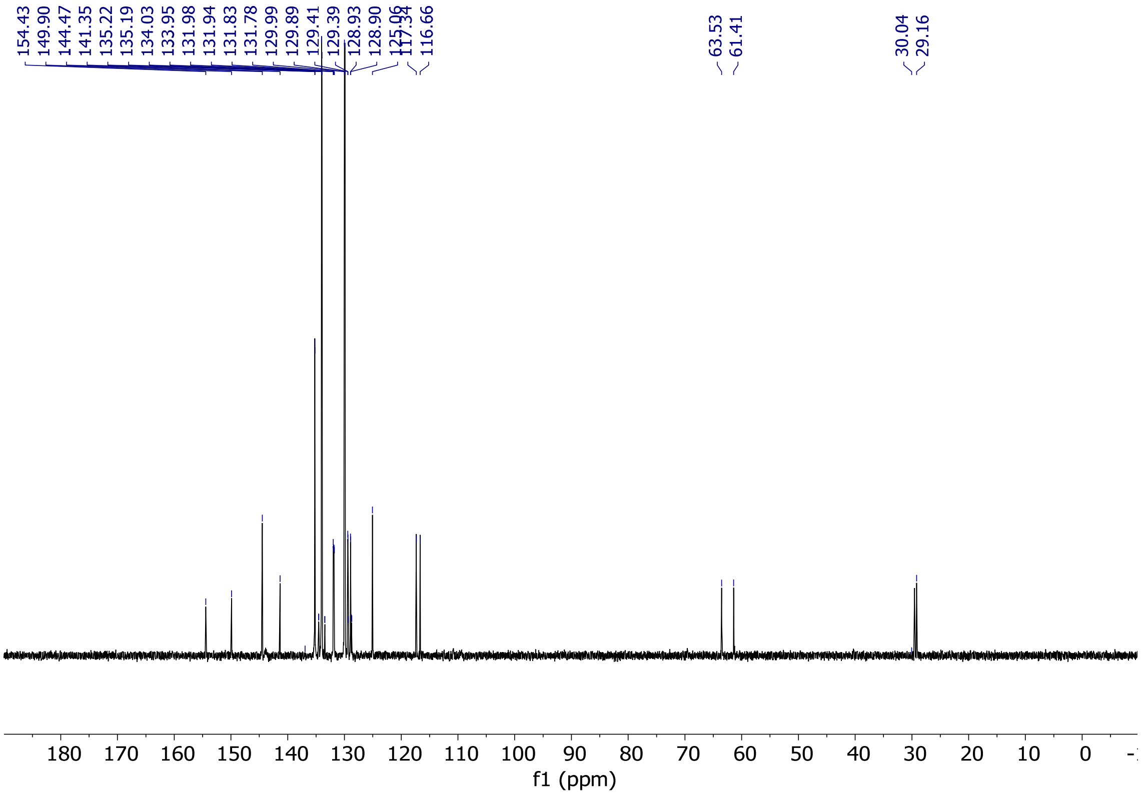

*Figure S33 $^{13}C\{^1H\}$ NMR (500 MHz, D_2O) spectrum of **2**·4Cl.*

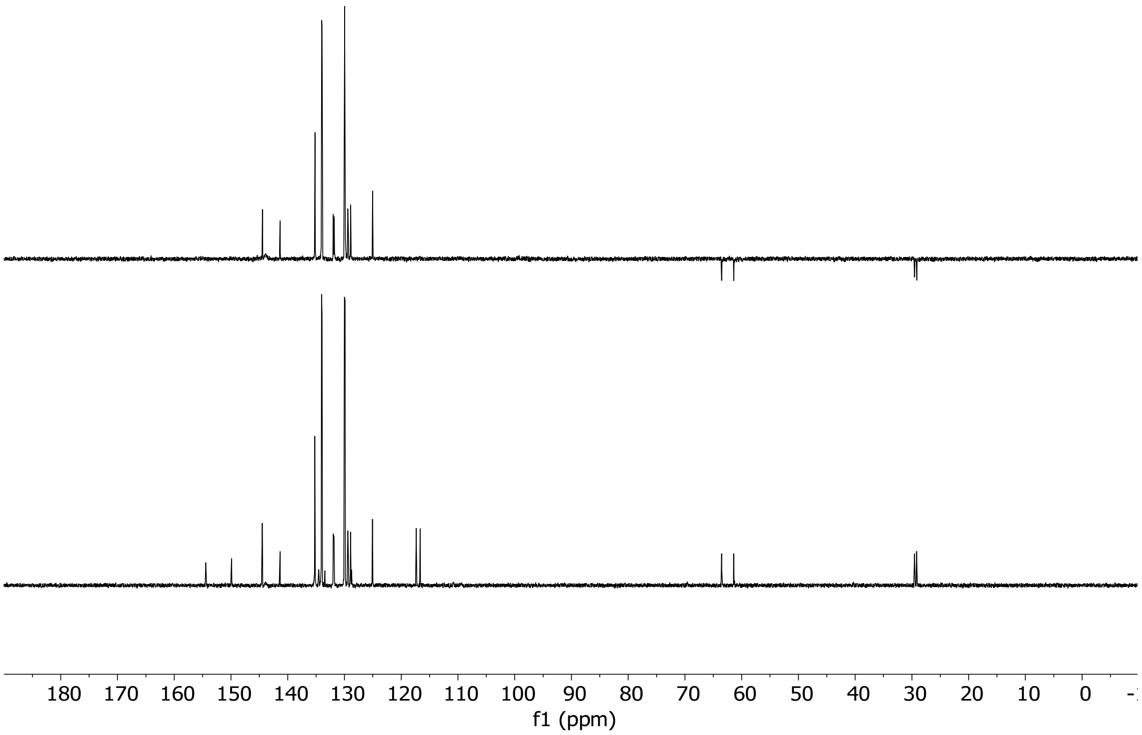

*Figure S34 $^{13}C\{^1H\}$ and DEPT 135$\{^1H\}$ NMR (500 MHz, D_2O) spectrum of **2**·4Cl.*

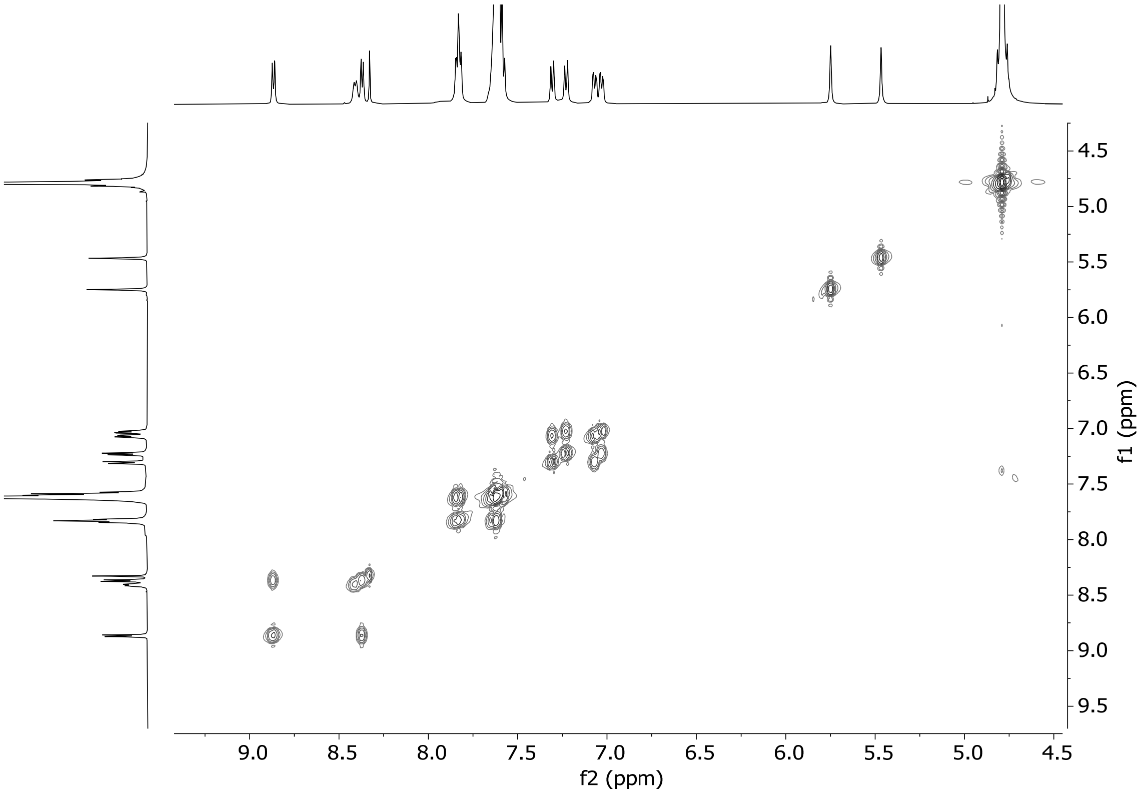

*Figure S35 COSY (500 MHz, D_2O) spectrum of **2**·4Cl.*

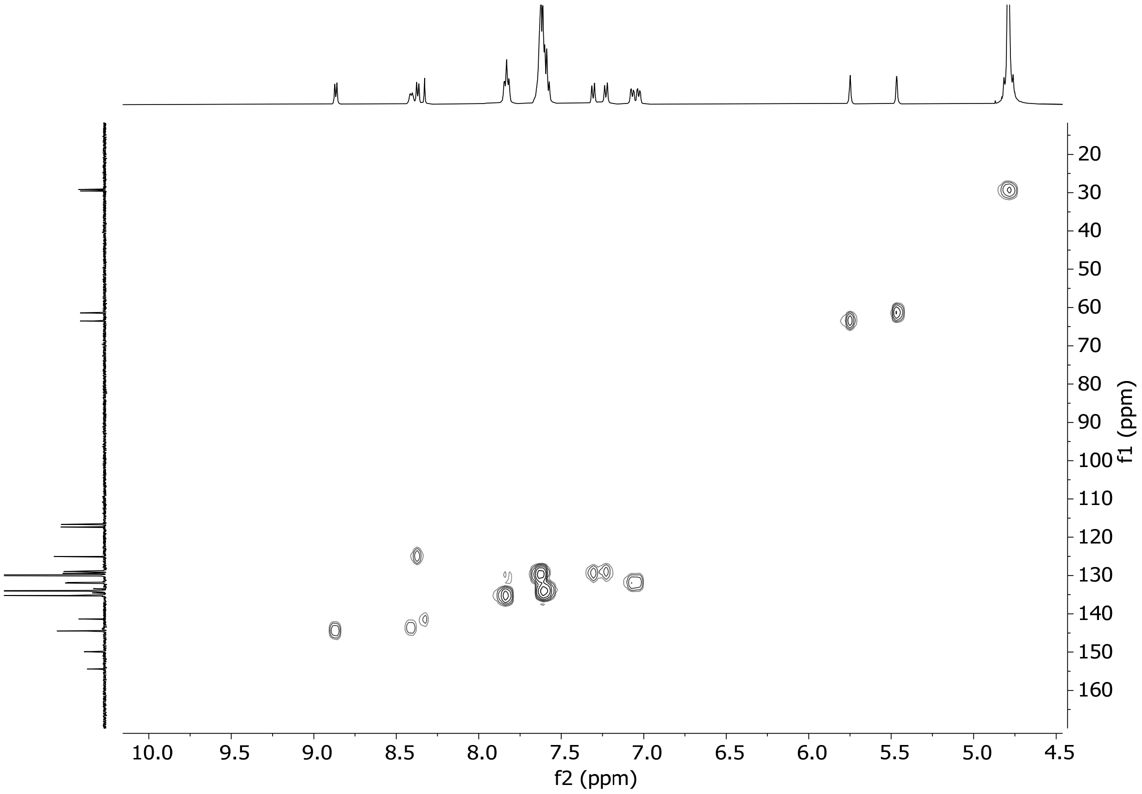

*Figure S36 HSQC (500 MHz, D_2O) spectrum of **2**·4Cl.*

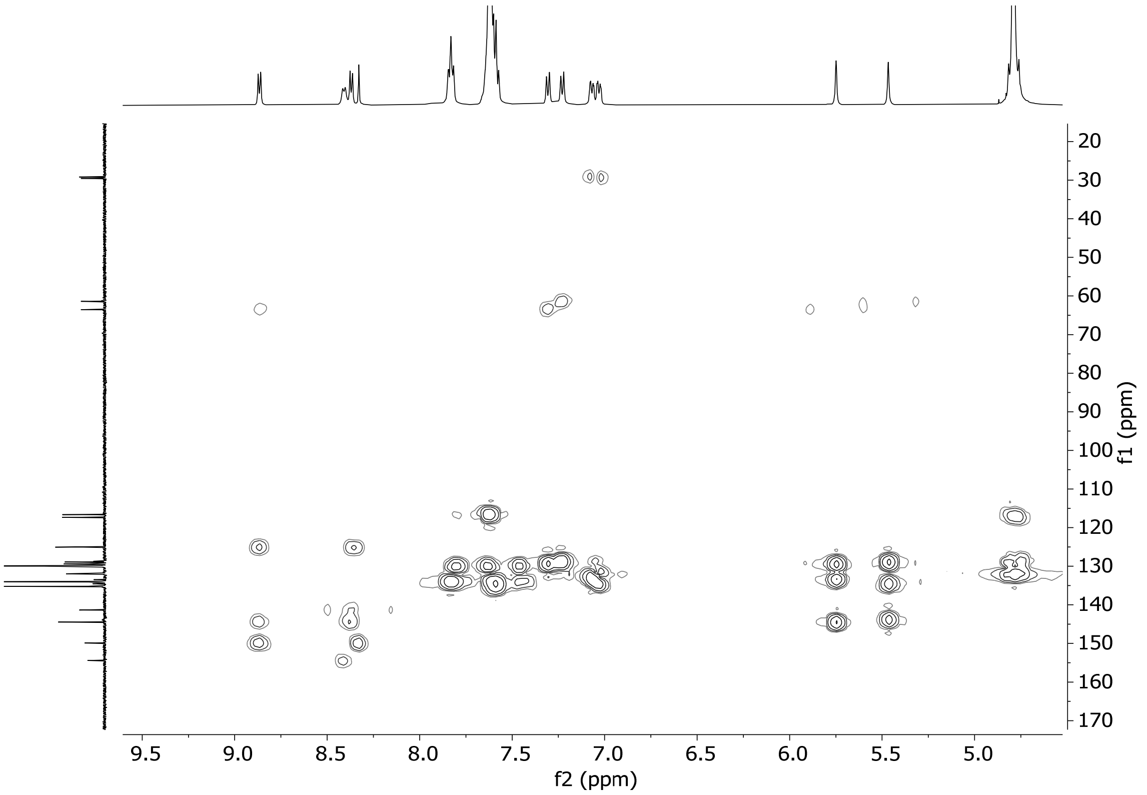

*Figure S37 HMBC (500 MHz, D_2O) spectrum of **2**·4Cl.*

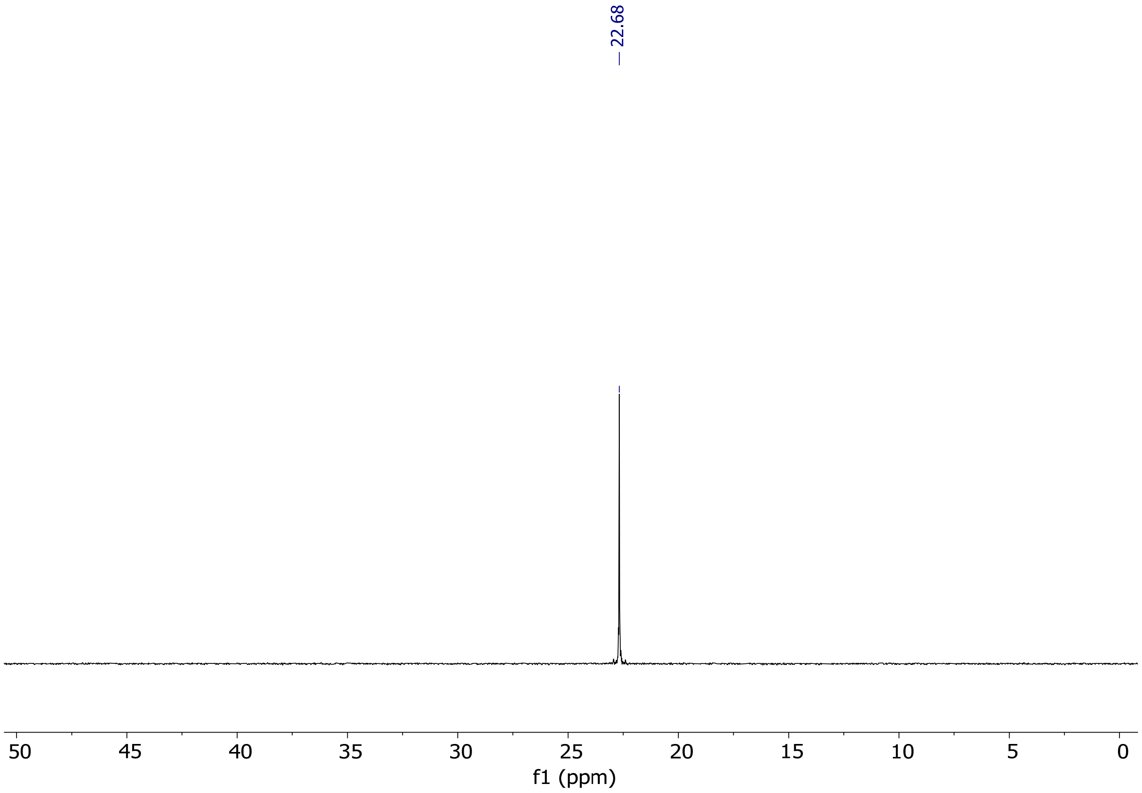

*Figure S38 $^{31}P\{^1H\}$ NMR (160 MHz, D_2O) spectrum of **2**·4Cl.*

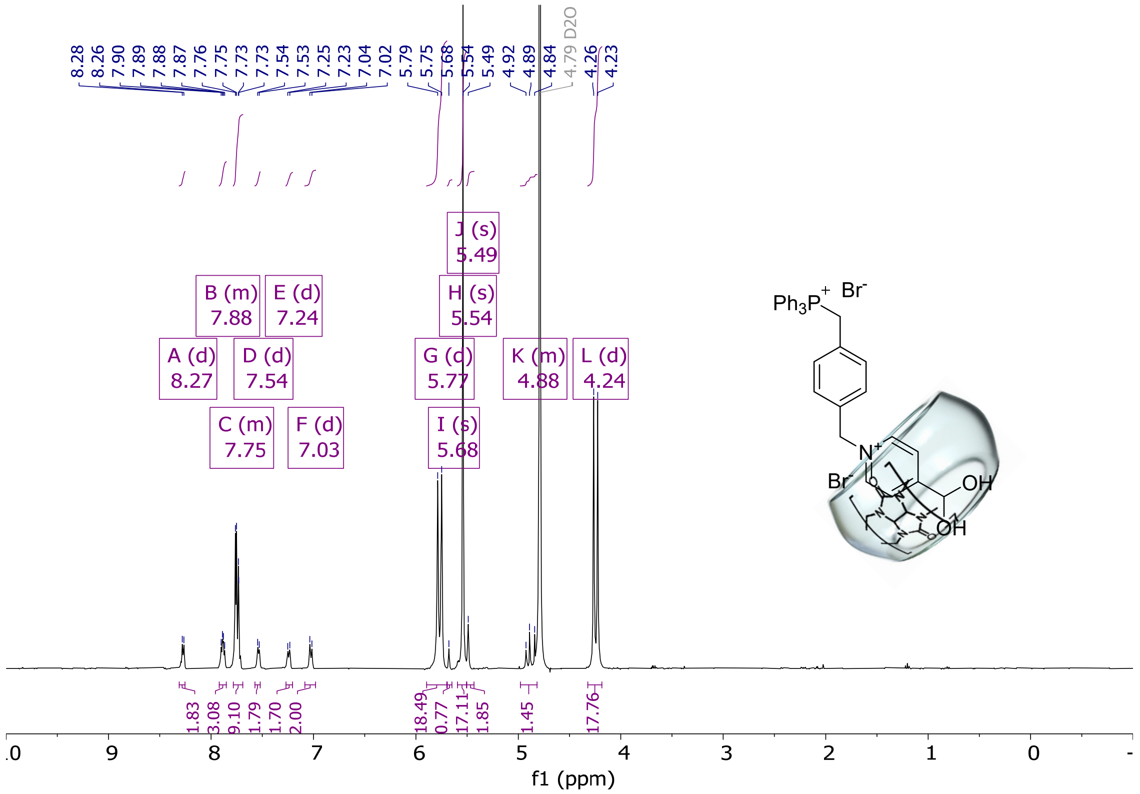

*Figure S39 ^{1}H NMR (500 MHz, D_2O) spectrum of **1ₐ**·2Br⊂CB[7].*

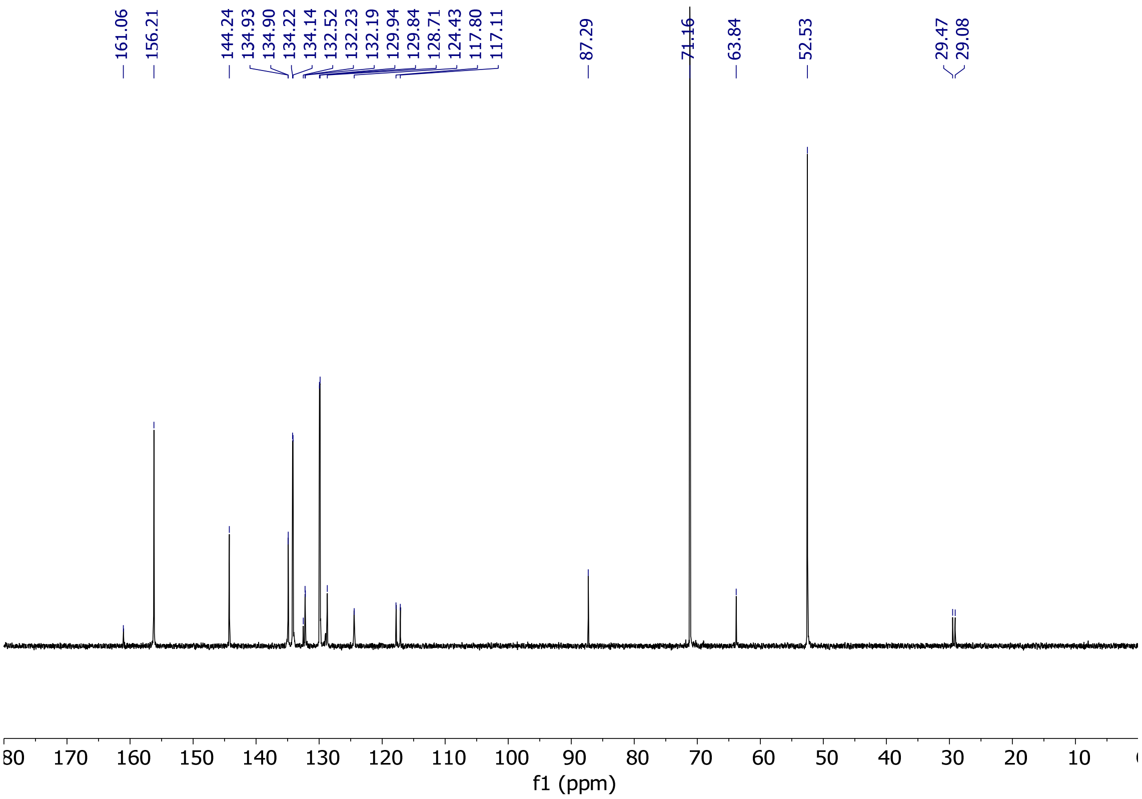

*Figure 40. $^{13}C\{^{1}H\}$ NMR (500 MHz, D_2O) spectrum of **1ₐ**·2Br⊂CB[7].*

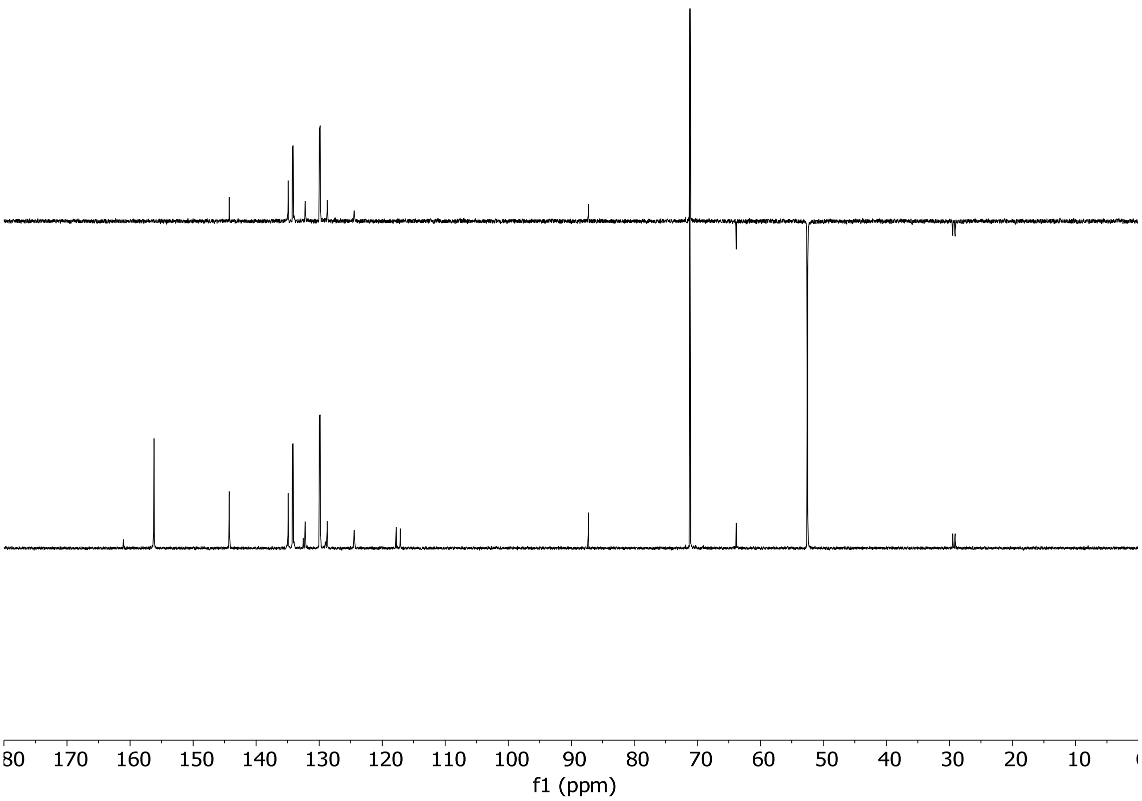

Figure 41. $^{13}C\{^{1}H\}$ and DEPT 135$\{^{1}H\}$ NMR (500 MHz, D_2O) spectrum of ***1***$_a$*·2Br⊂CB[7].*

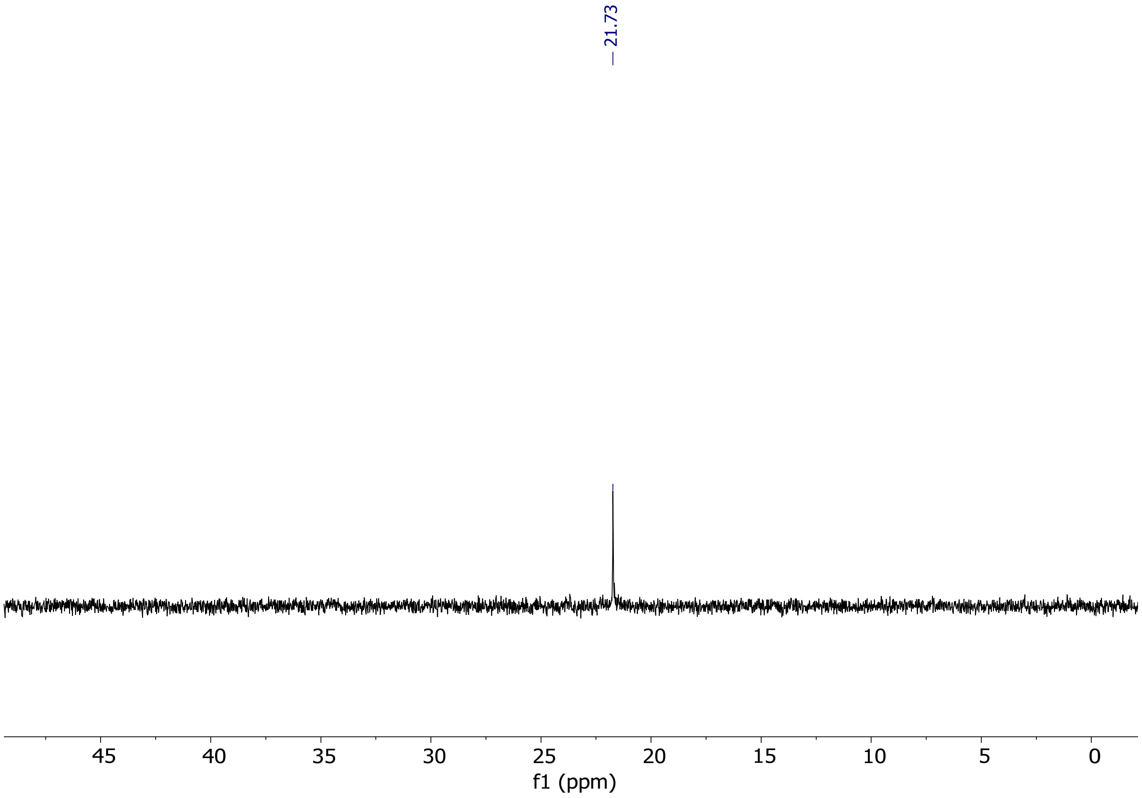

Figure S42 $^{31}P\{^{1}H\}$ NMR (160 MHz, D_2O) spectrum of ***1***$_a$*·2Br⊂CB[7].*

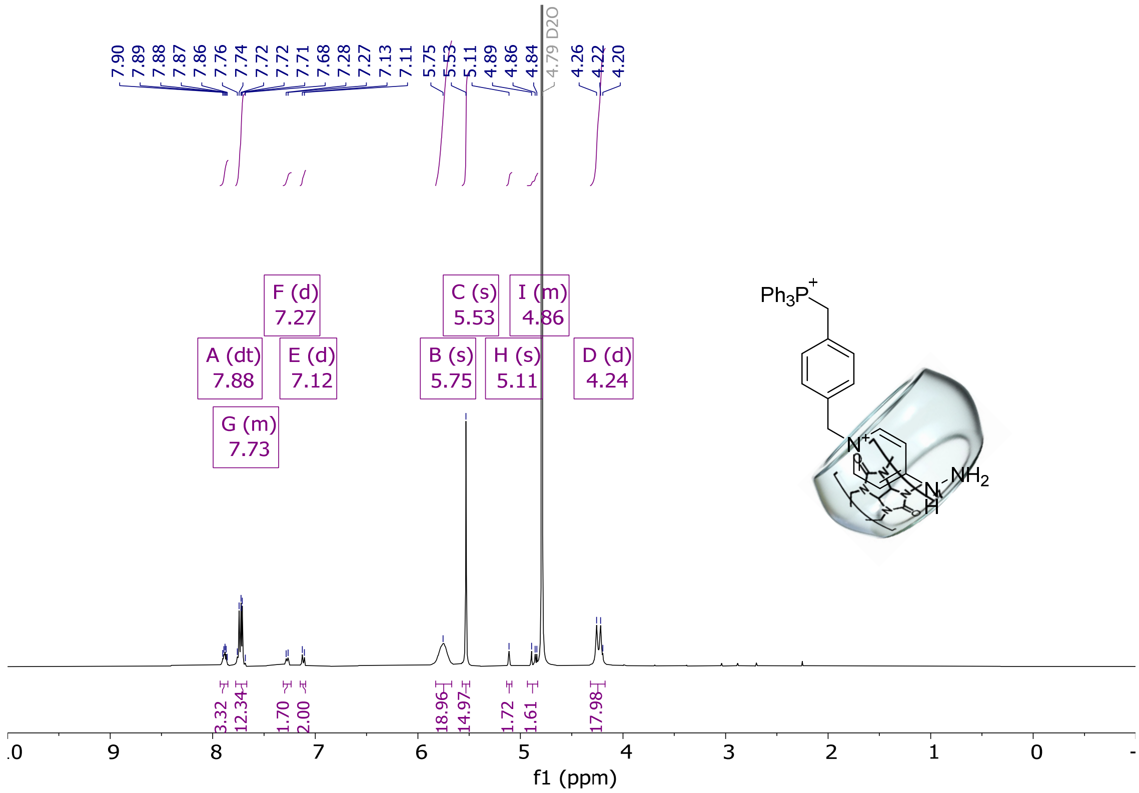

Figure S43 ^{1}H NMR (500 MHz, D_2O) spectrum of $\mathbf{1_b}$·2Br⊂CB[7]

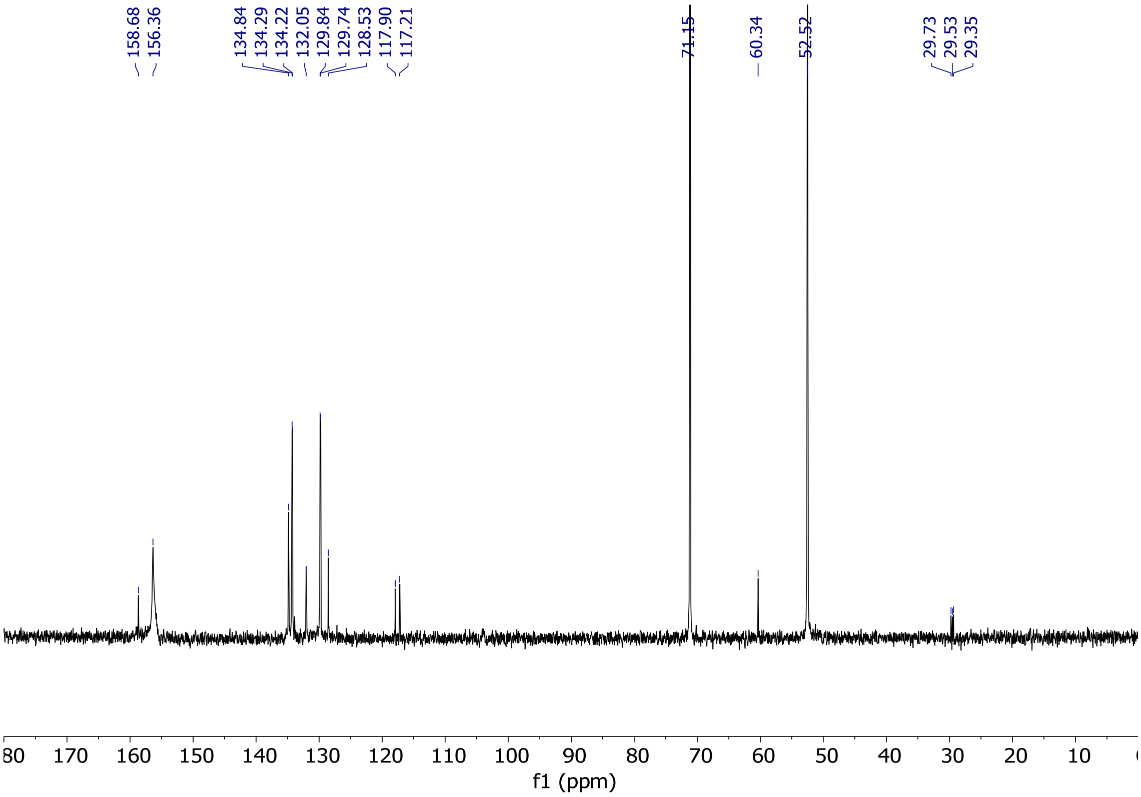

Figure 44 $^{13}C\{^{1}H\}$ NMR (500 MHz, D_2O) spectrum of $\mathbf{1_b}$·2Br⊂CB[7].

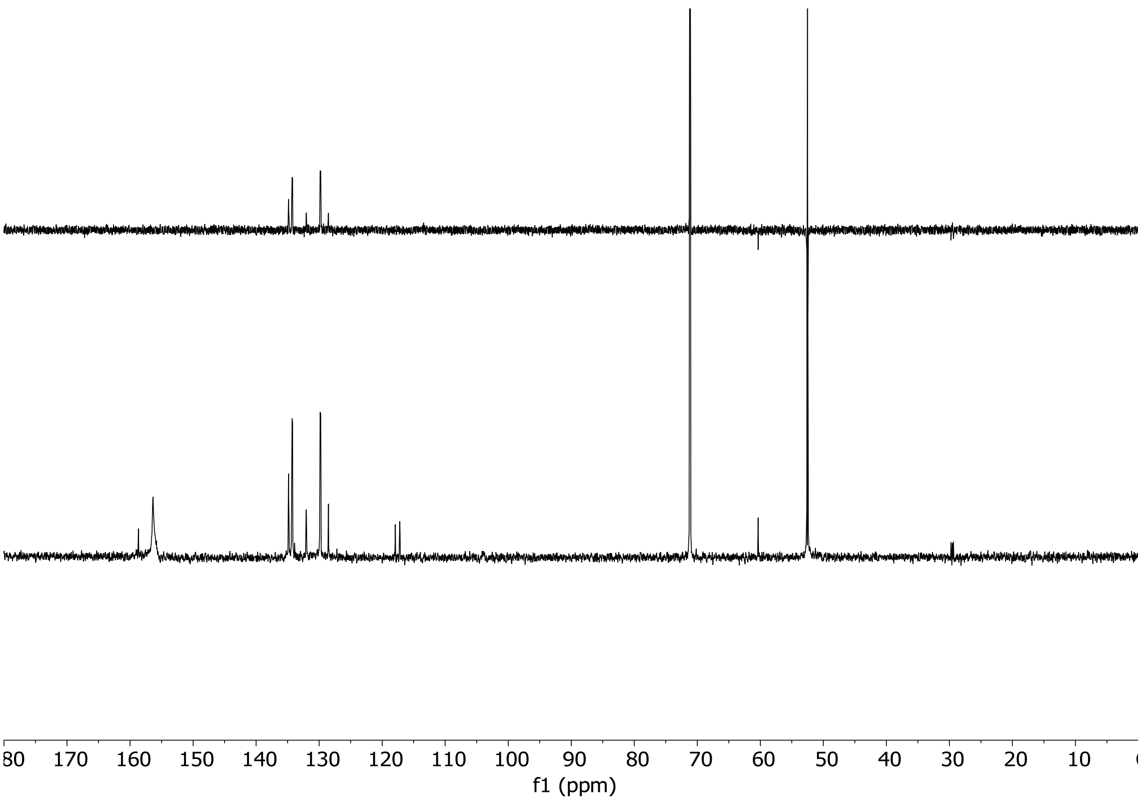

Figure 45 $^{13}C\{^{1}H\}$ and DEPT 135$\{^{1}H\}$ NMR (500 MHz, D_2O) spectrum of ***1_b****·2Br⊂CB[7].*

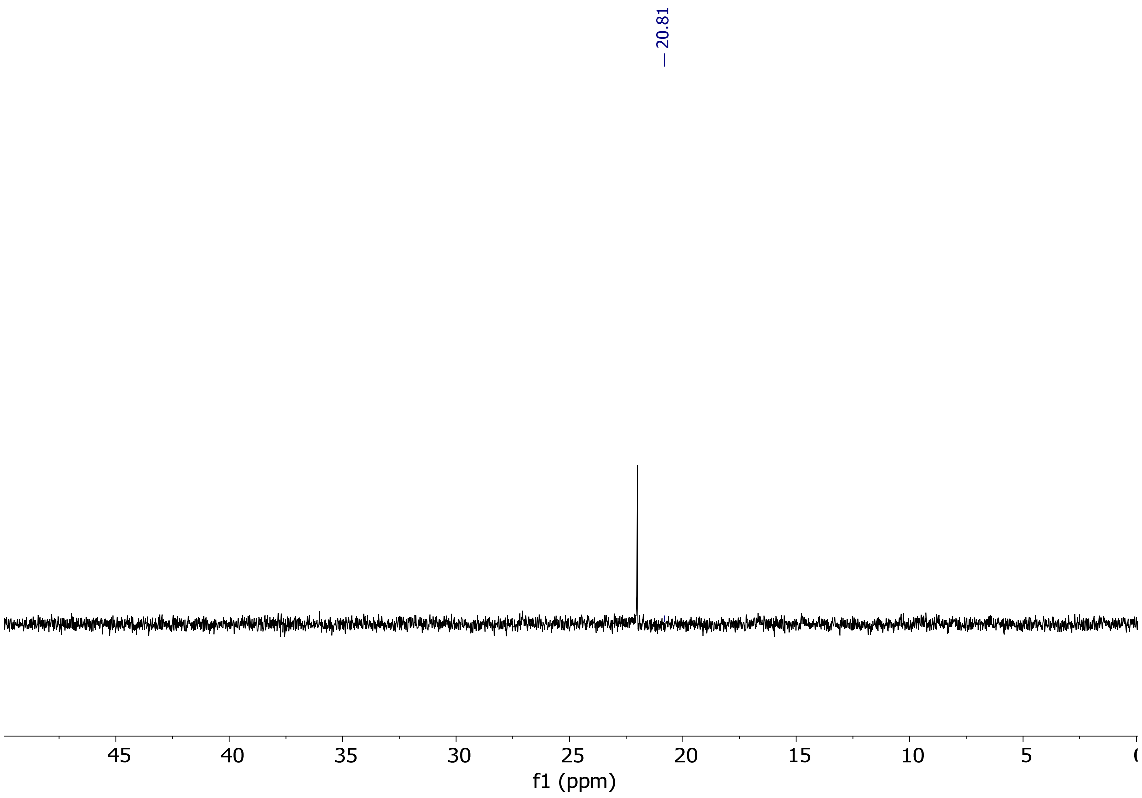

Figure S46 $^{31}P\{^{1}H\}$ NMR (160 MHz, D_2O) spectrum of ***1_b****·2Br⊂CB[7].*

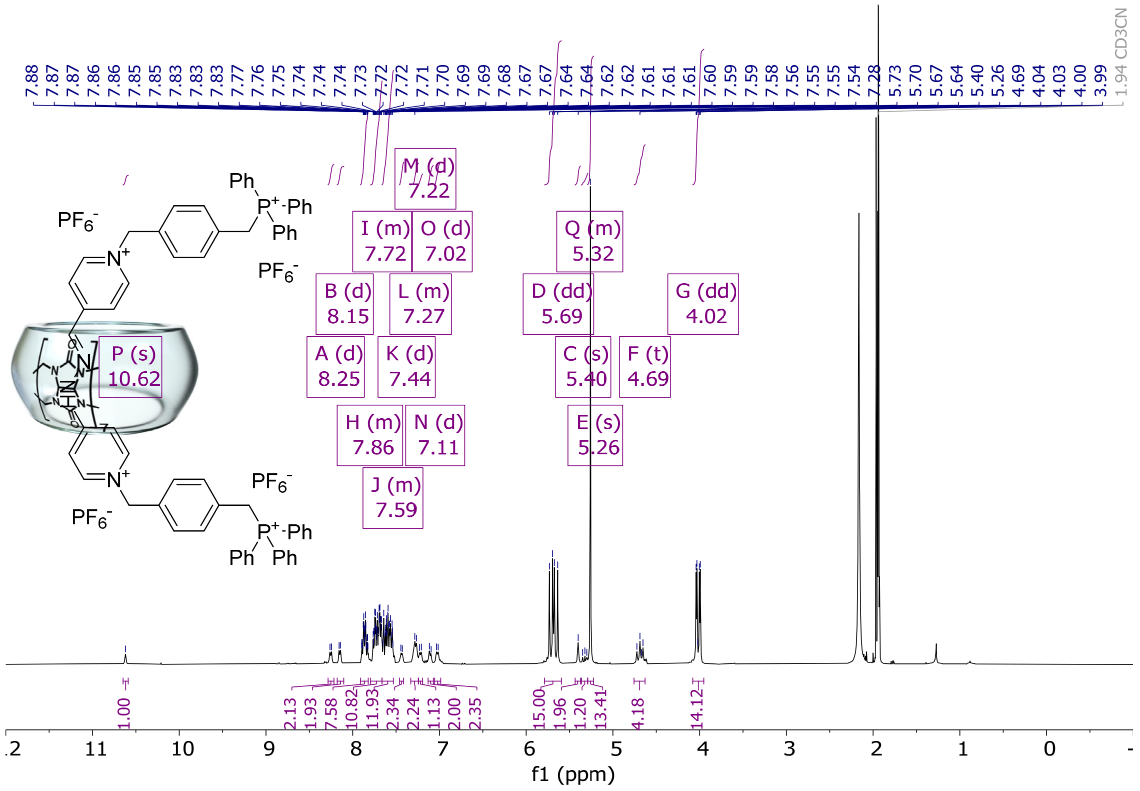

*Figure S47 ^{1}H NMR (500 MHz, CD_3CN) spectrum of **2**·$4PF_6$.*

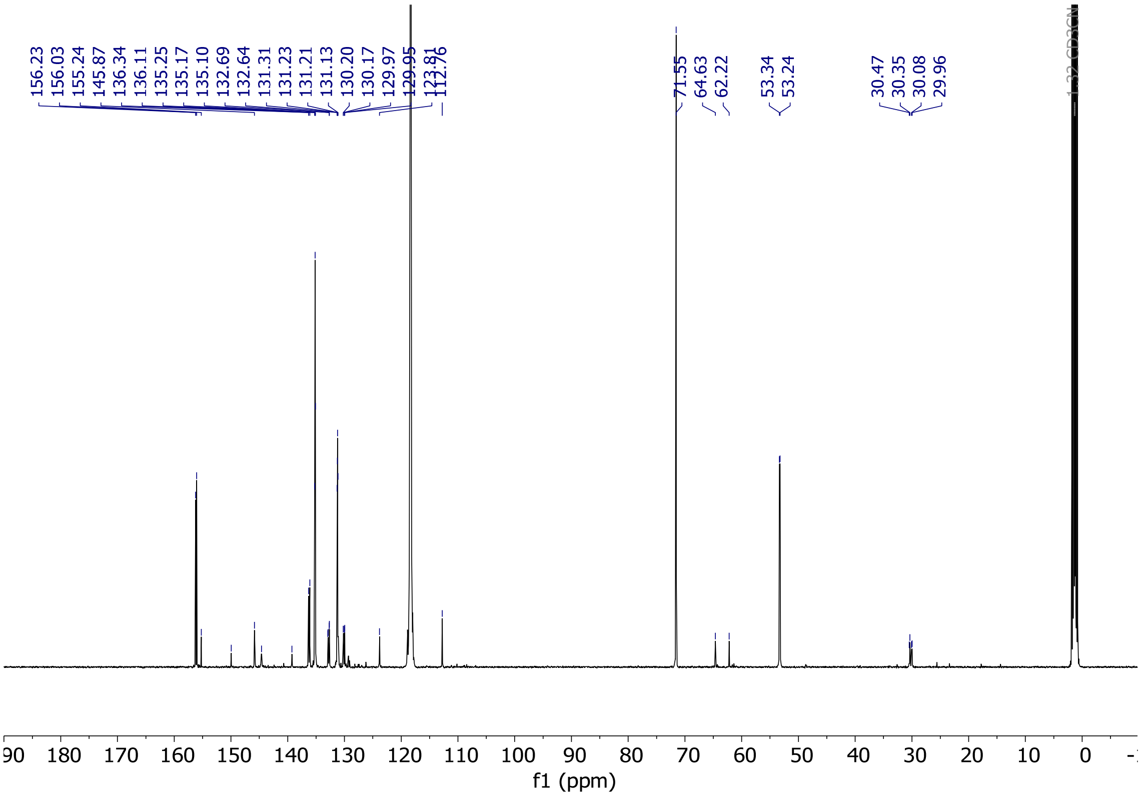

*Figure S48 $^{13}C\{^{1}H\}$ NMR (125 MHz, CD_3CN) spectrum of **2**·$4PF_6$.⊂CB[7].*

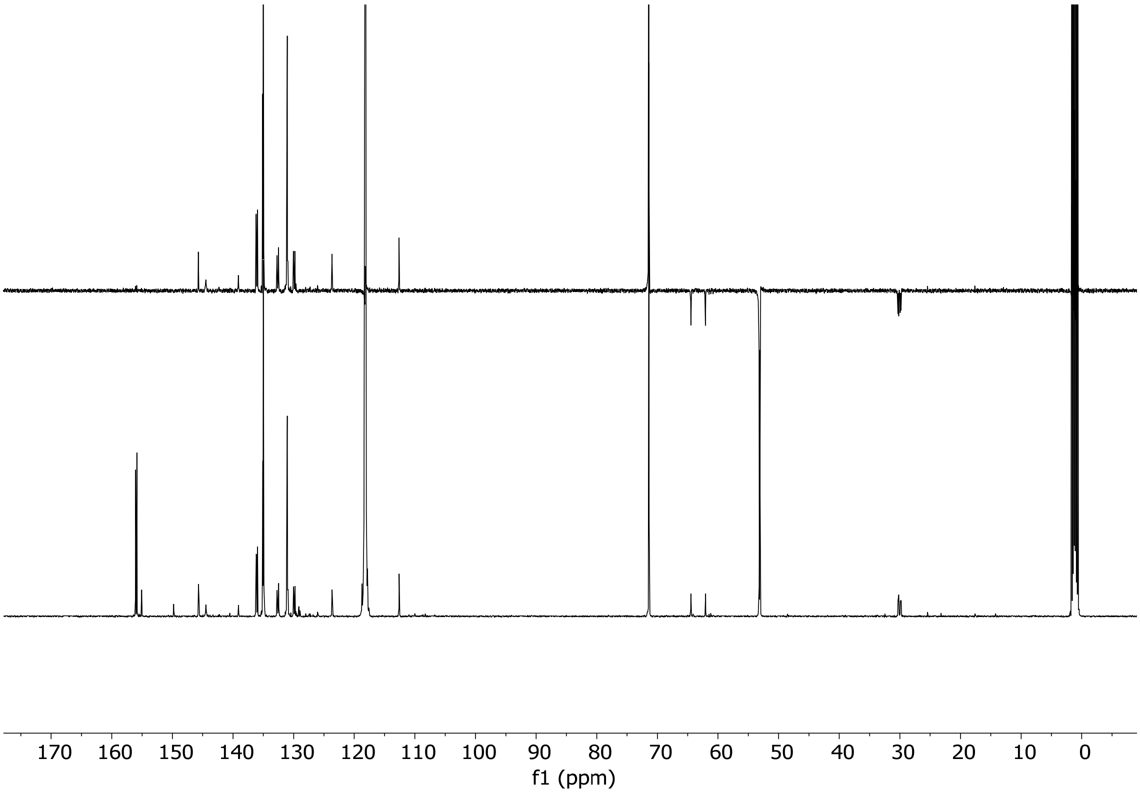

*Figure S49 $^{13}C\{^{1}H\}$ and DEPT 135$\{^{1}H\}$ NMR (125 MHz, CD_3CN) spectrum of **2**·4PF_6.⊂CB[7].*

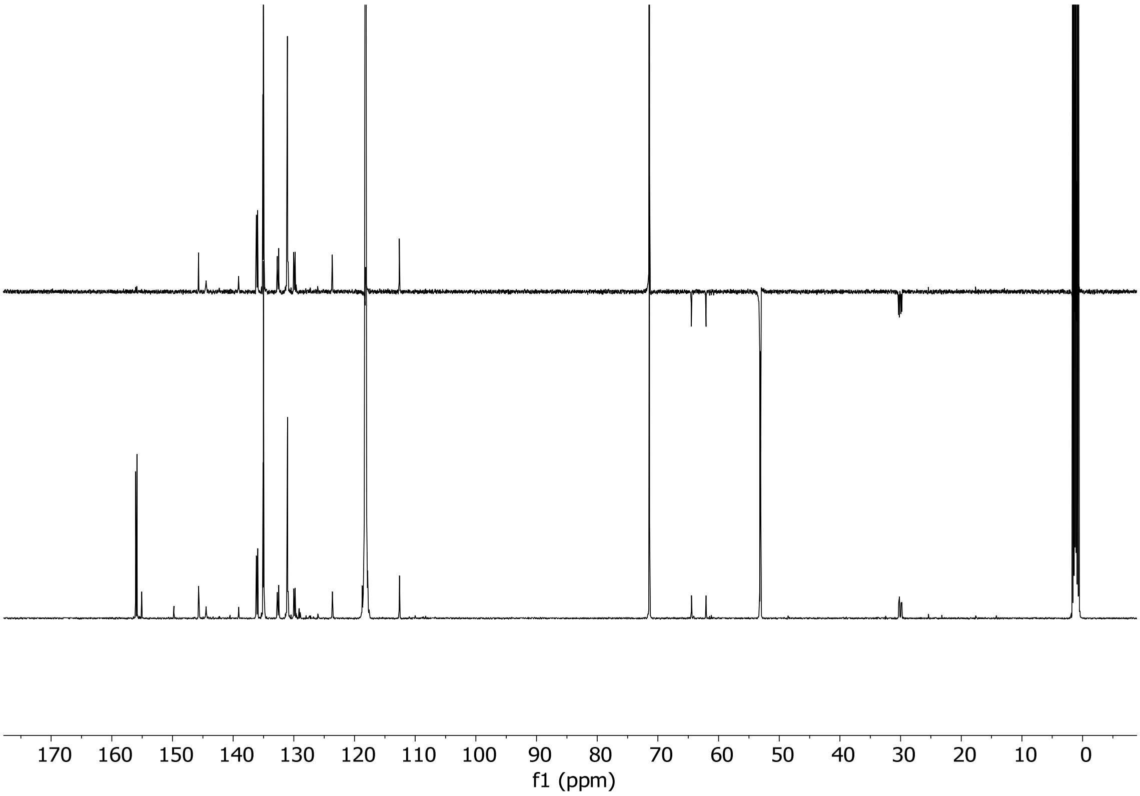

*Figure S50 COSY (500 MHz, CD_3CN) spectrum of **2**·4PF_6.⊂CB[7].*

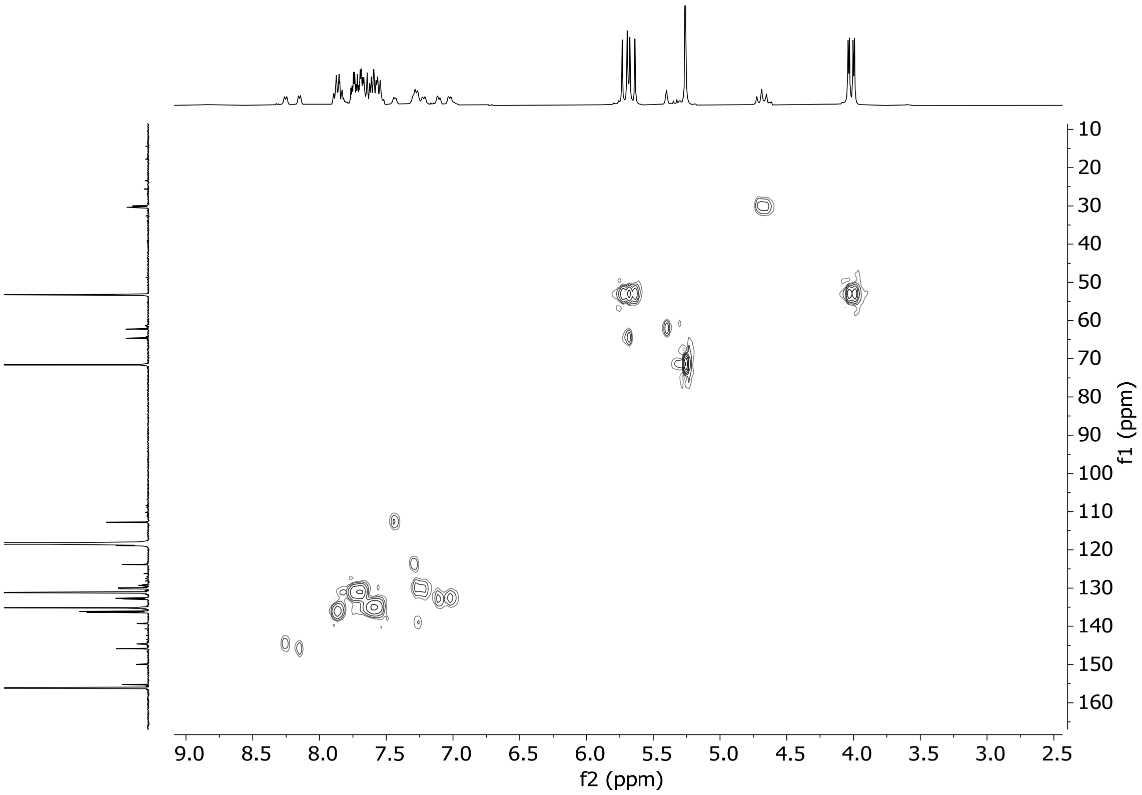

*Figure S51 HSQC(500 MHz, CD_3CN) spectrum of **2**·4PF_6.⊂CB[7].*

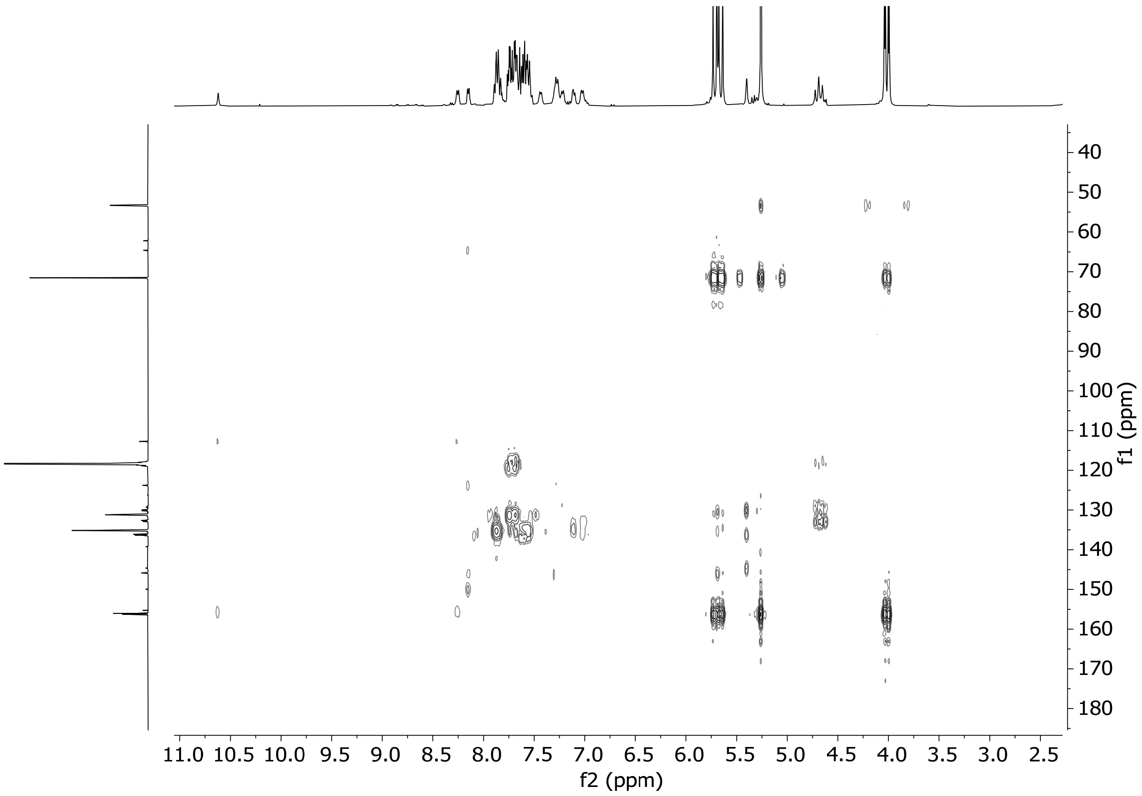

*Figure S52 HMBC (500 MHz, CD_3CN) spectrum of **2**·4PF_6.⊂CB[7].*

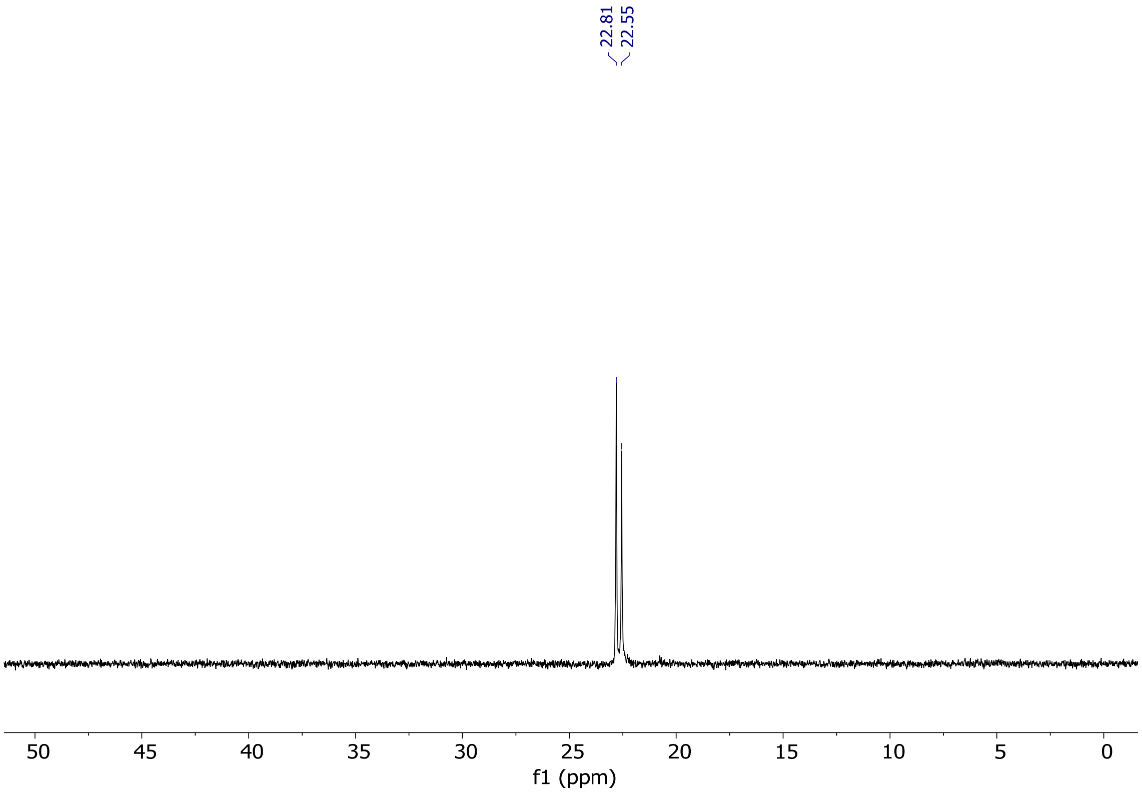

*Figure S53 ^{31}P NMR (160 MHz, CD_3CN) spectrum of **2**·$4PF_6$.⊂CB[7].*

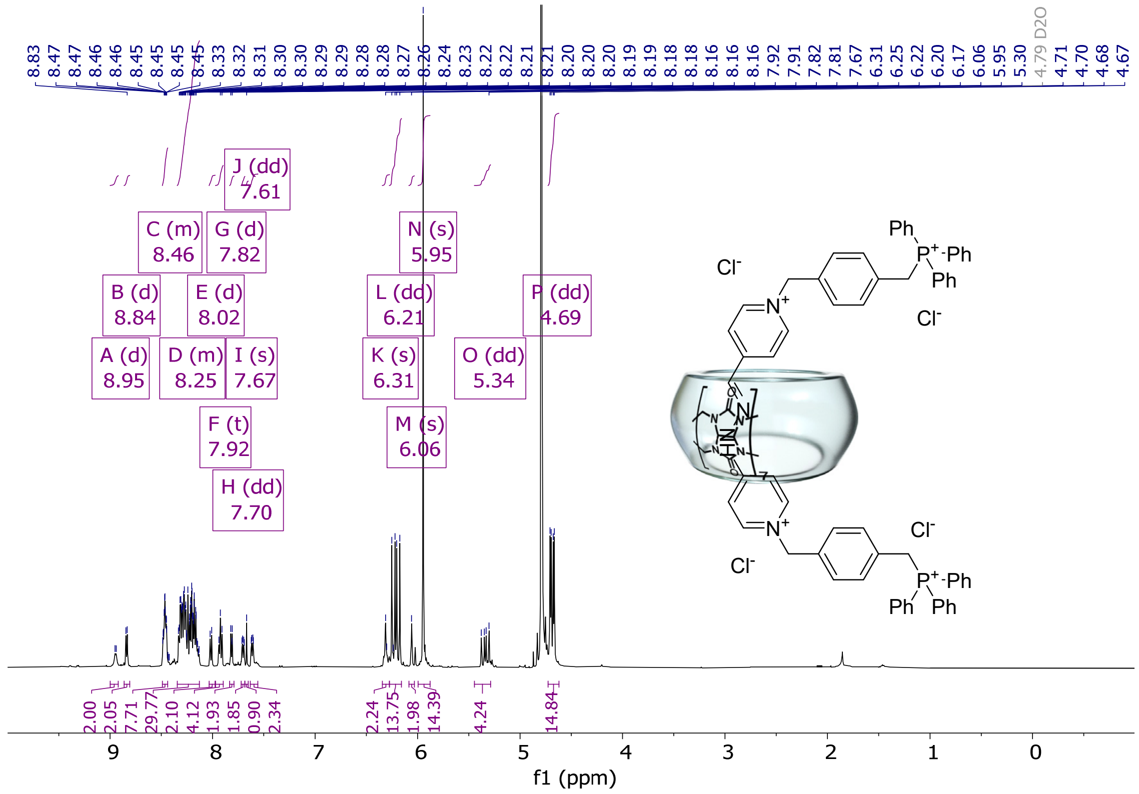

*Figure S54 ^{1}H NMR (500 MHz, D_2O) spectrum of **2**·4Cl⊂CB[7].*

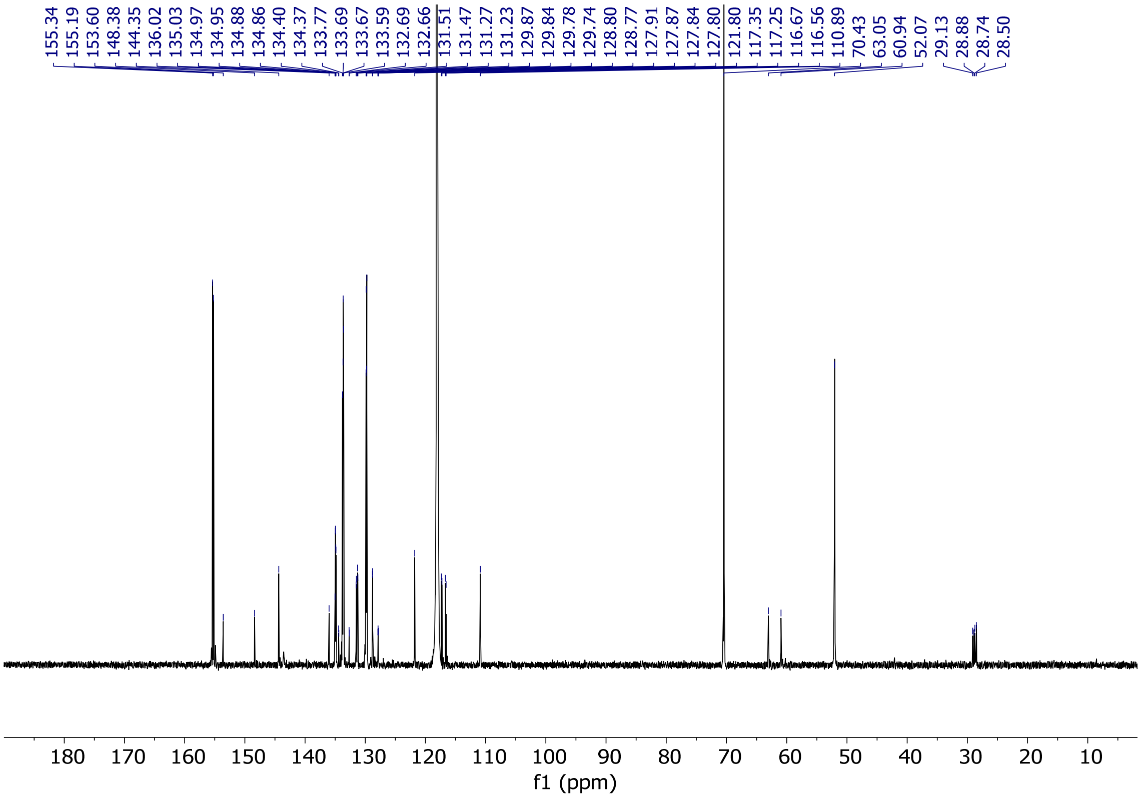

*Figure S55 $^{13}C\{^1H\}$ NMR (125 MHz, D_2O) spectrum of **2**·4Cl⊂CB[7]*

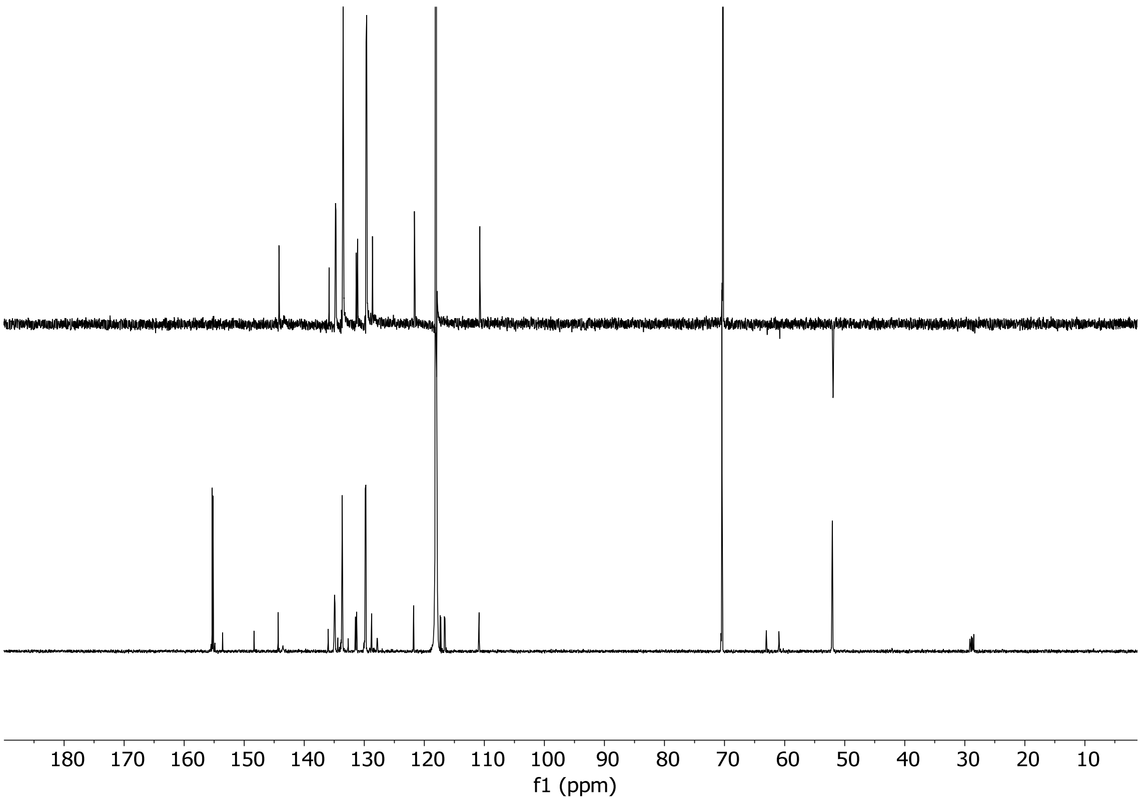

*Figure S56 $^{13}C\{^1H\}$ and DEPT 135$\{^1H\}$ NMR (125 MHz, D_2O) spectrum of **2**·4Cl⊂CB[7]*

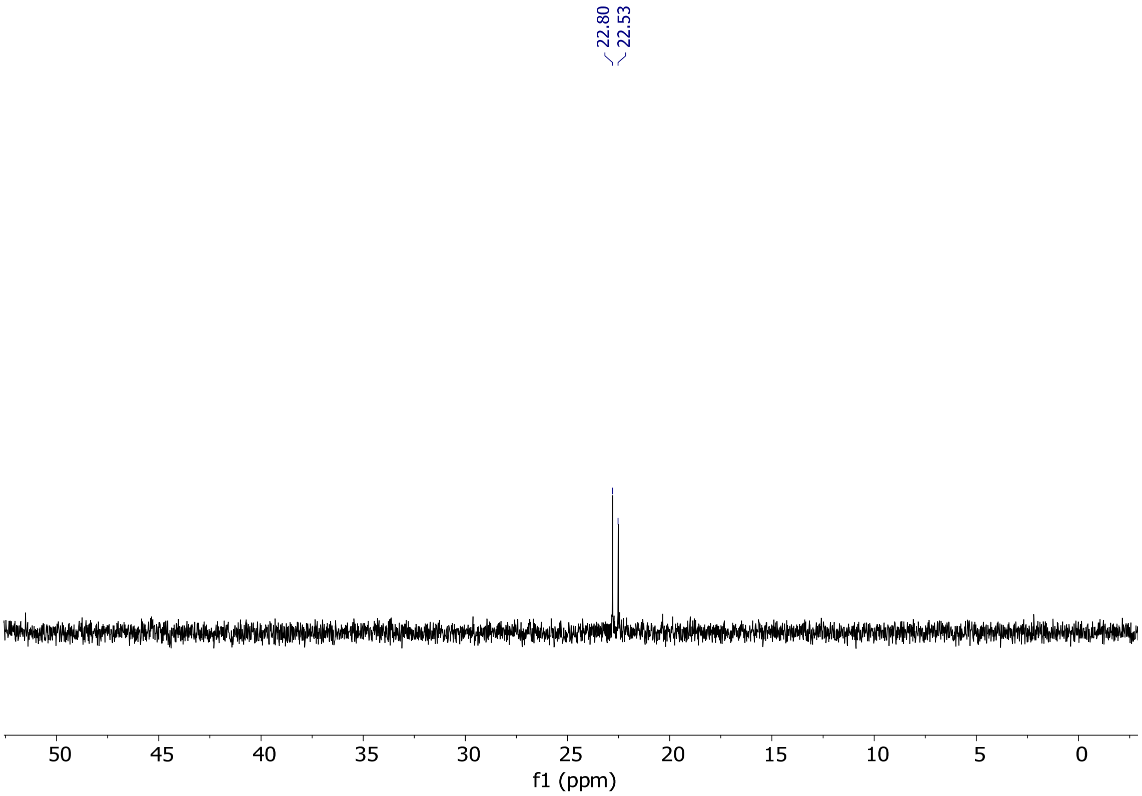

*Figure S57 $^{31}P\{^{1}H\}$ NMR (160 MHz, D_2O) spectrum of **2**·4Cl⊂CB[7].*

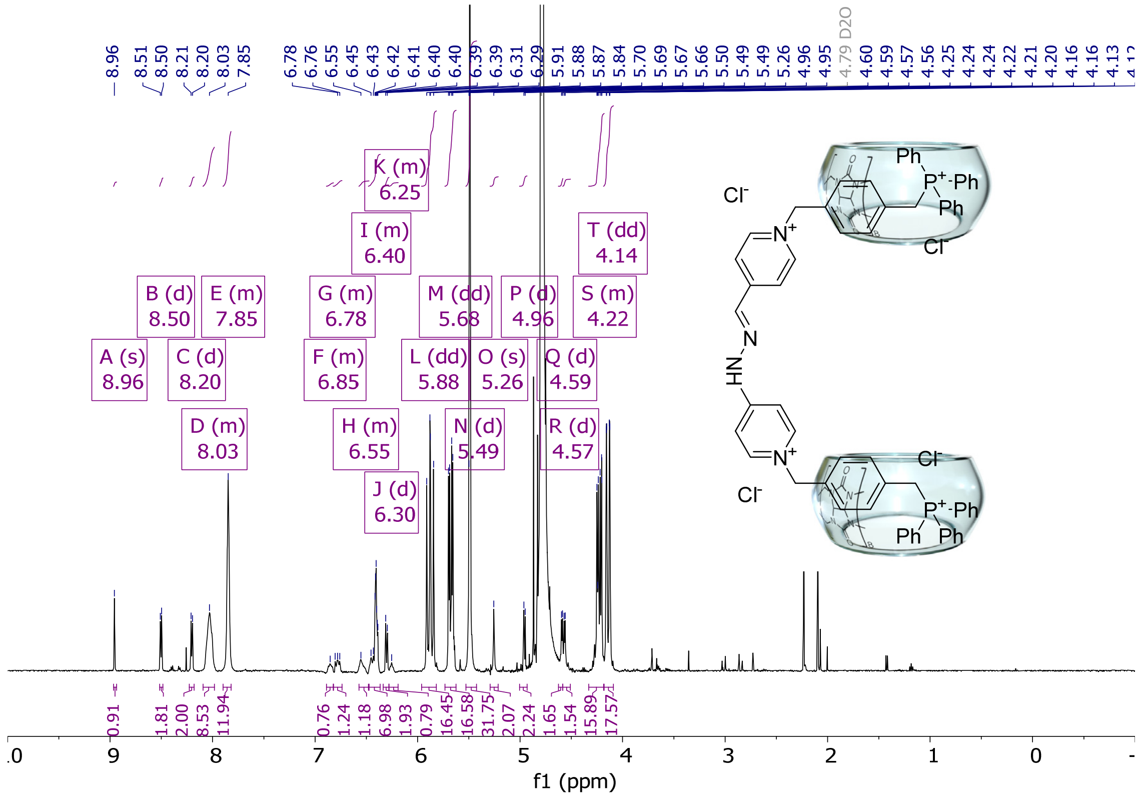

*Figure S58 ^{1}H NMR (500 MHz, D_2O) spectrum of **2**·4Cl⊂(CB[8])$_2$.*

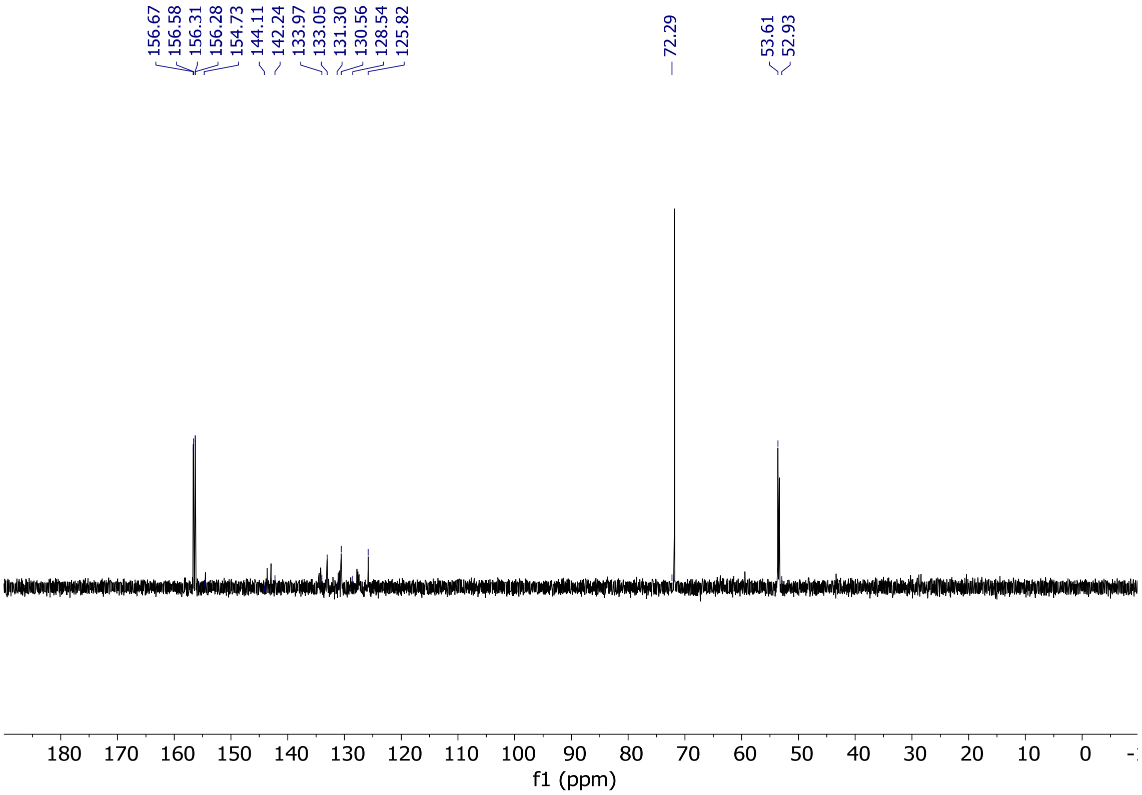

*Figure S59 $^{13}C\{^1H\}$ NMR (125 MHz, D_2O) spectrum of **2**·4Cl⊂(CB[8])$_2$.*

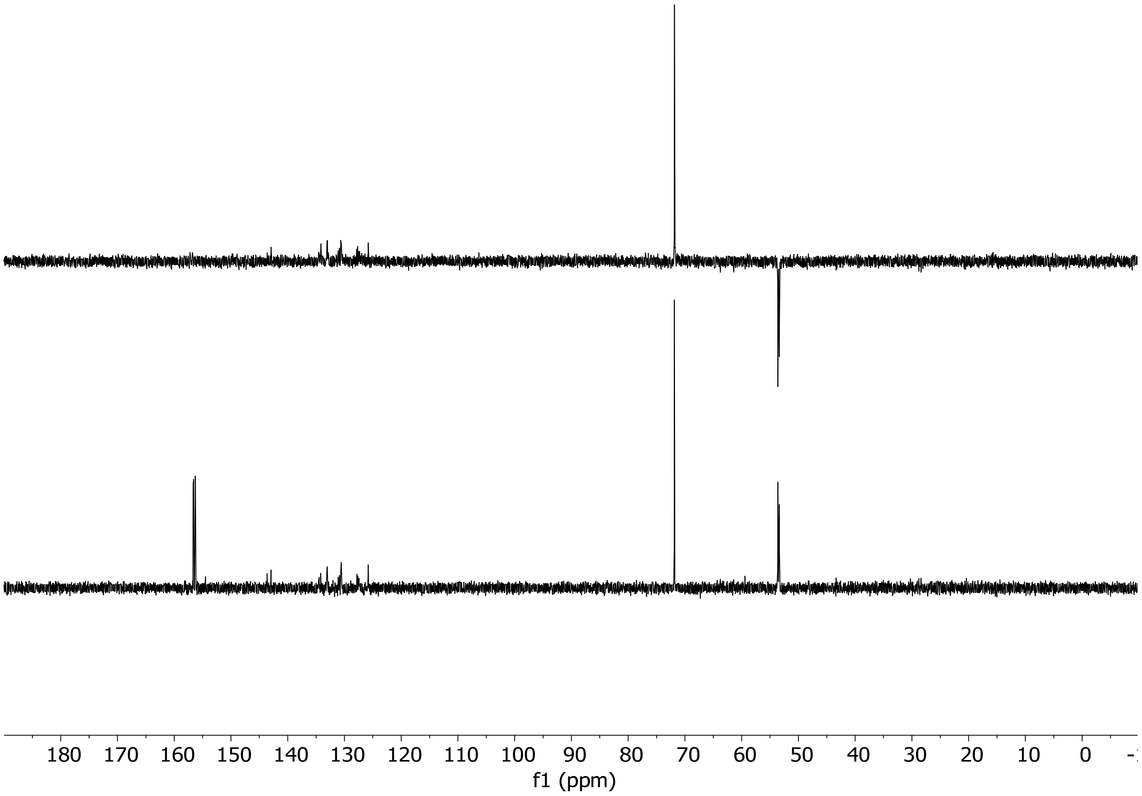

*Figure S60 $^{13}C\{^1H\}$ and DEPT 135$\{^1H\}$ NMR (125 MHz, D_2O) spectrum of **2**·4Cl⊂(CB[8])$_2$.*

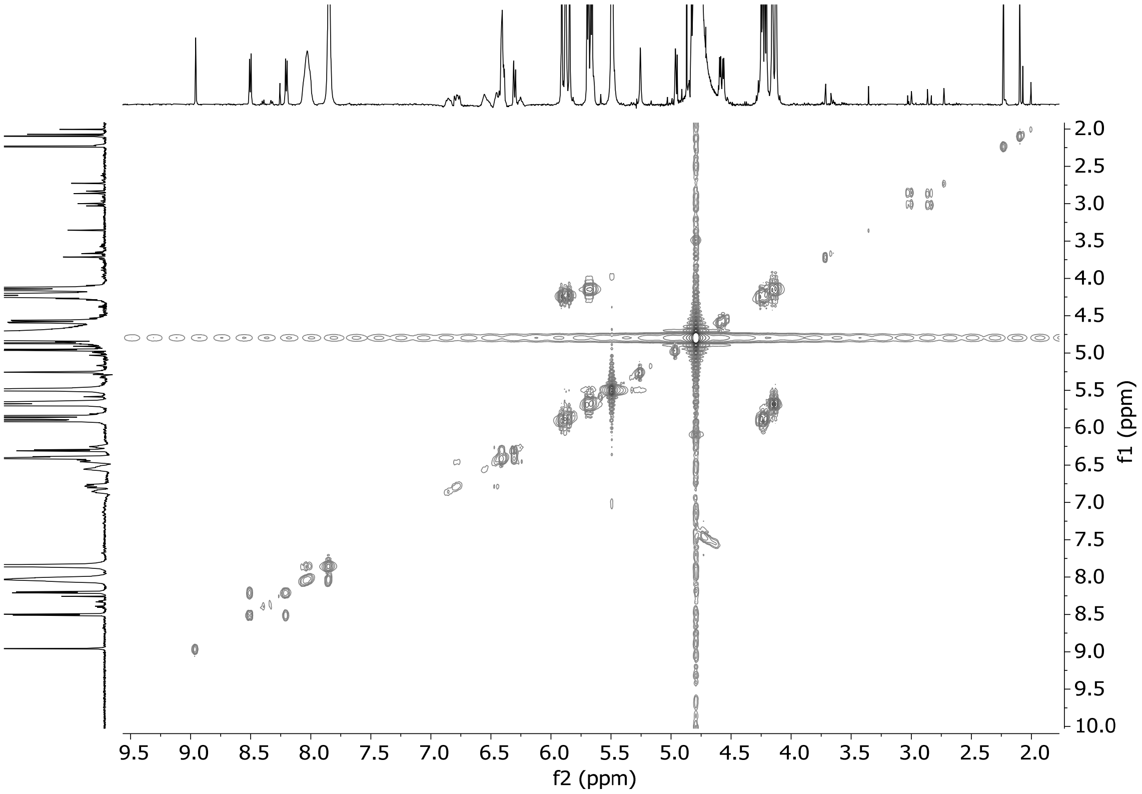

*Figure S61 COSY (500 MHz, D_2O) spectrum of **2**·4Cl⊂(CB[8])$_2$.*

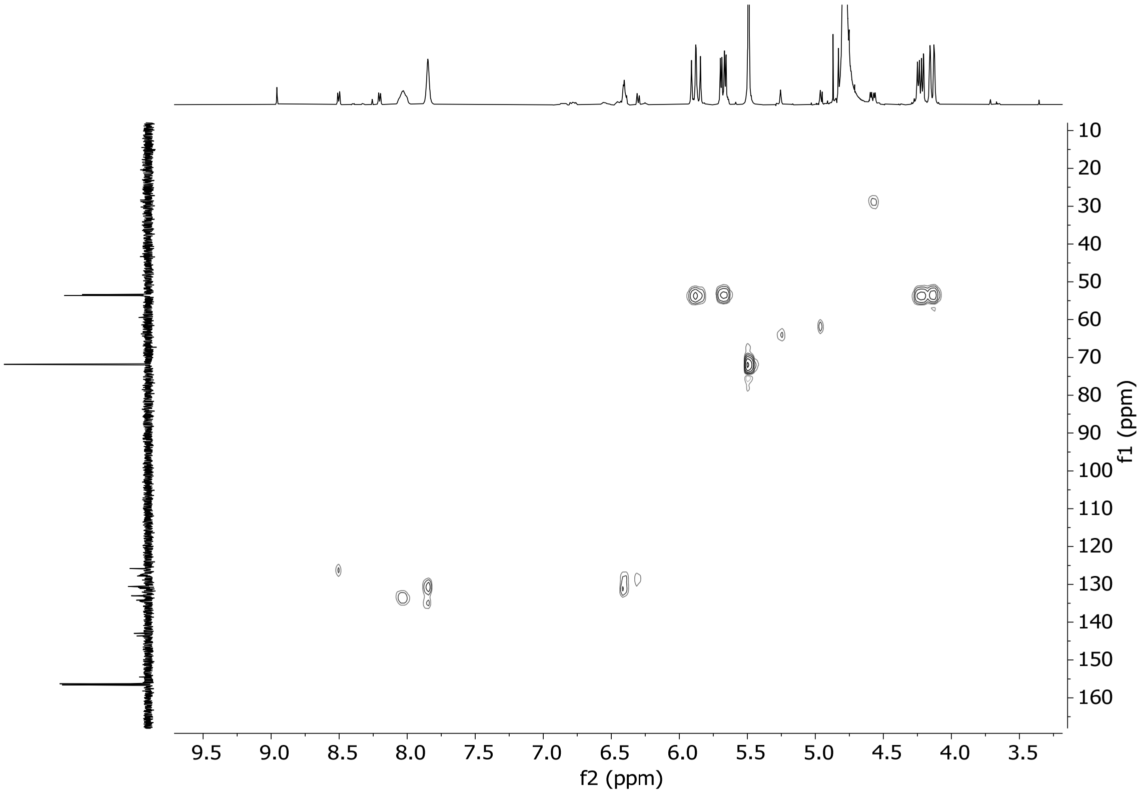

*Figure S62 HSQC (500 MHz, D_2O) spectrum of **2**·4Cl⊂(CB[8])$_2$.*

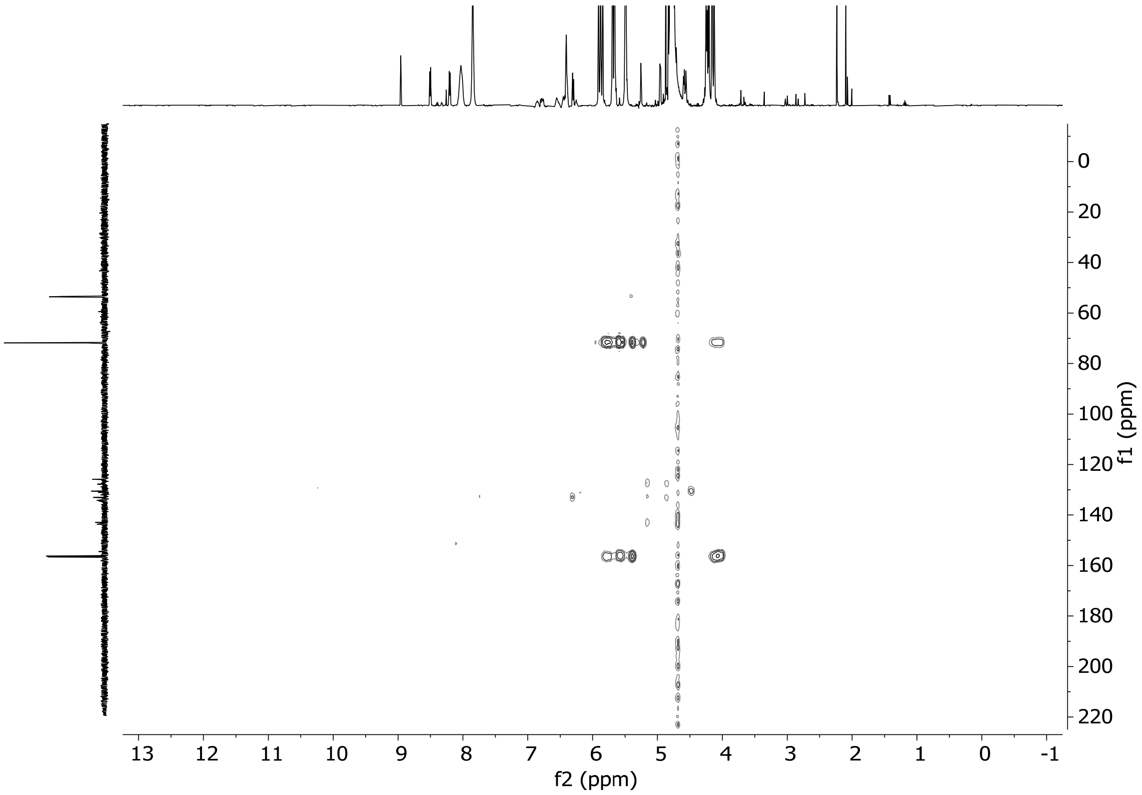

*Figure S63 HMBC (500 MHz, D_2O) spectrum of **2**·4Cl⊂(CB[8])$_2$.*

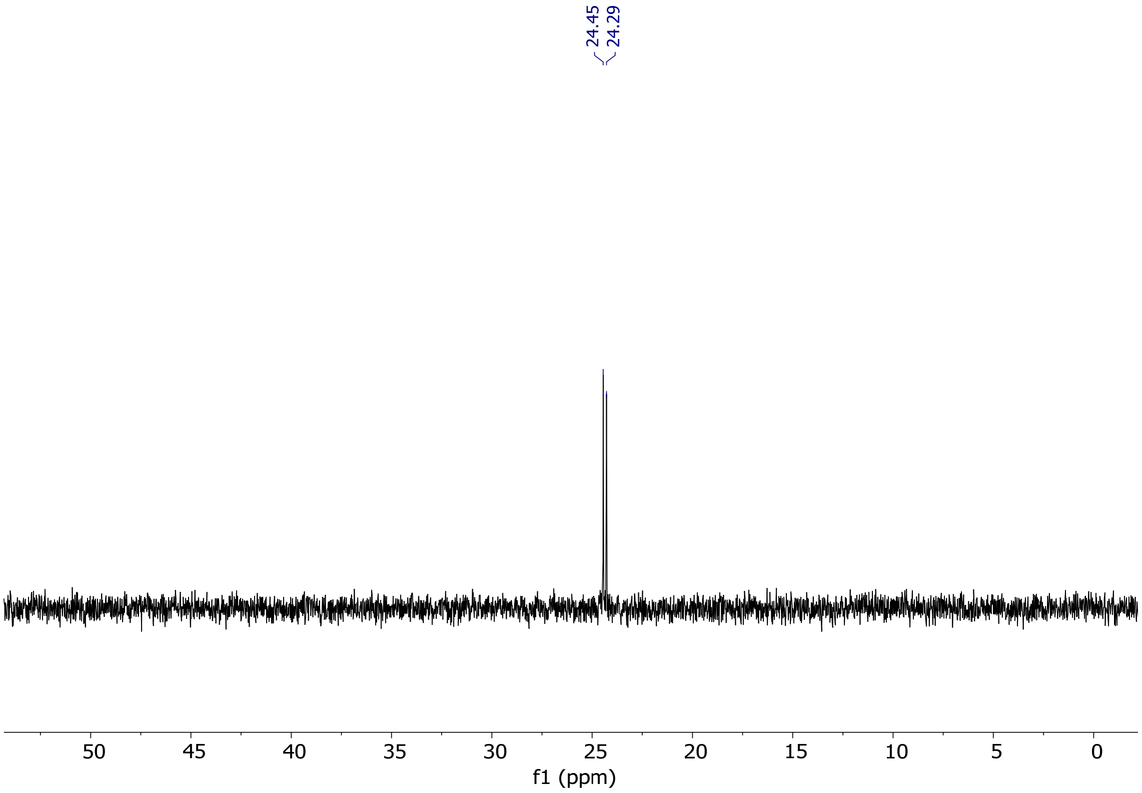

*Figure S64 $^{31}P\{^1H\}$ NMR (160 MHz, D_2O) spectrum of **2**·4Cl⊂(CB[8])$_2$.*

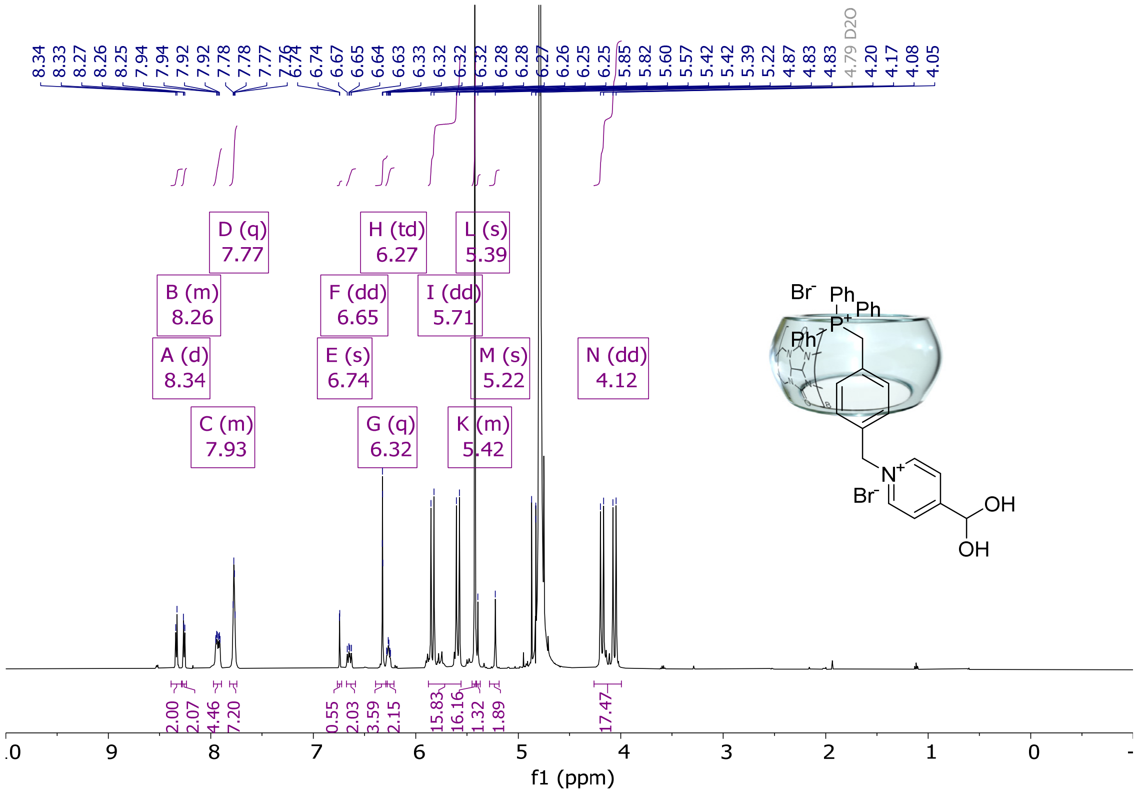

Figure S65 ^{1}H NMR (500 MHz, D_2O) spectrum of $\mathbf{1_a}$·2Br ⊂CB[8].

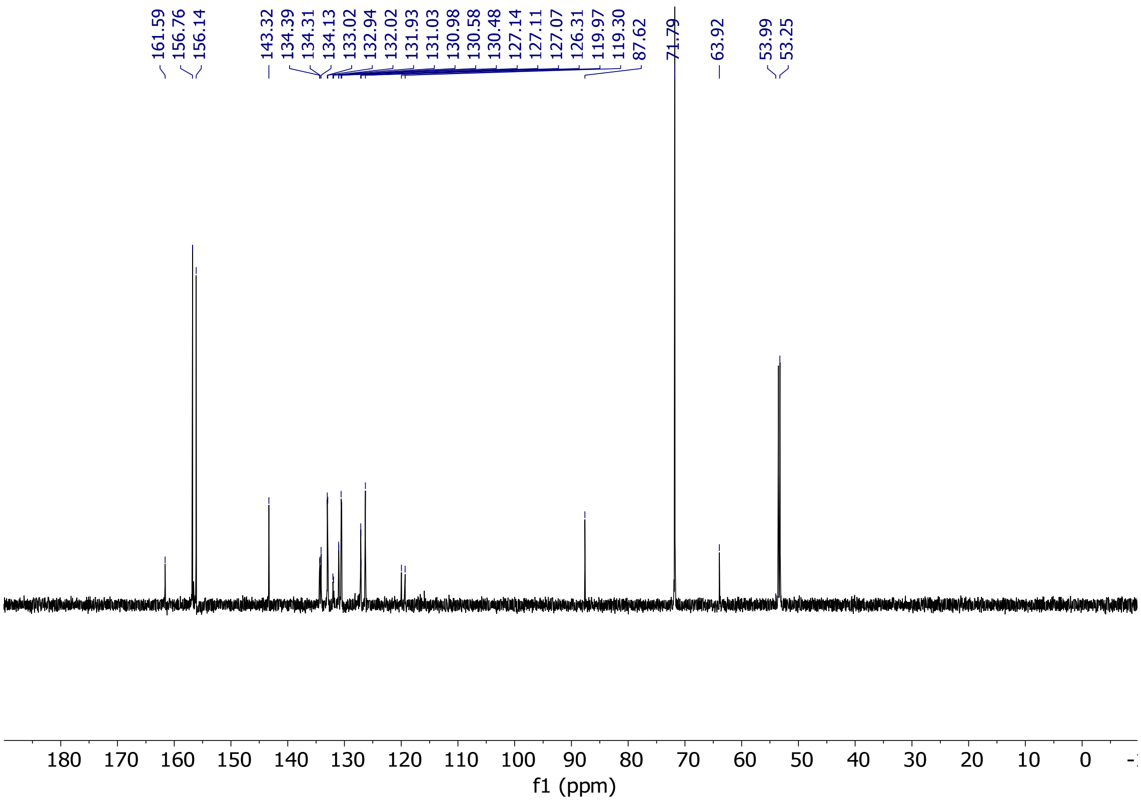

Figure S66 $^{13}C\{^{1}H\}$ NMR (125 MHz, D_2O) spectrum of $\mathbf{1_a}$·2Br ⊂CB[8].

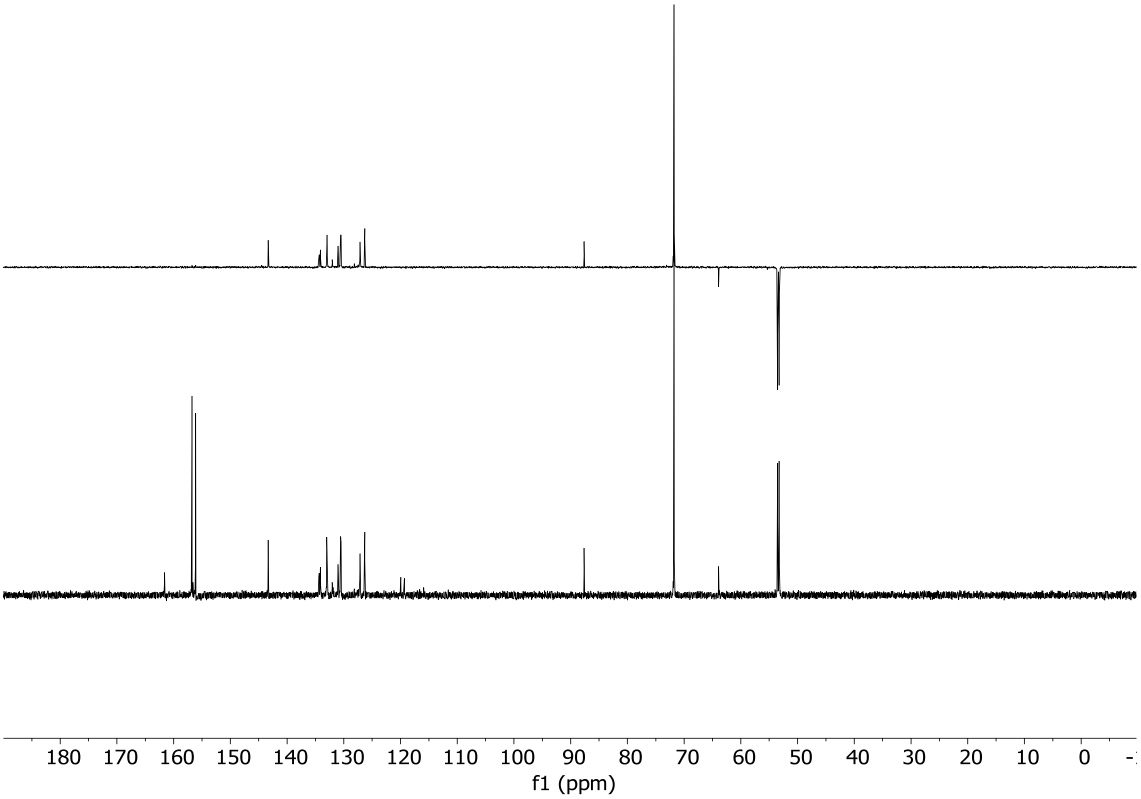

Figure S67 $^{13}C\{^{1}H\}$ and DEPT 135$\{^{1}H\}$ NMR (125 MHz, D_2O) spectrum of $\mathbf{1_a}$·2Br ⊂CB[8].

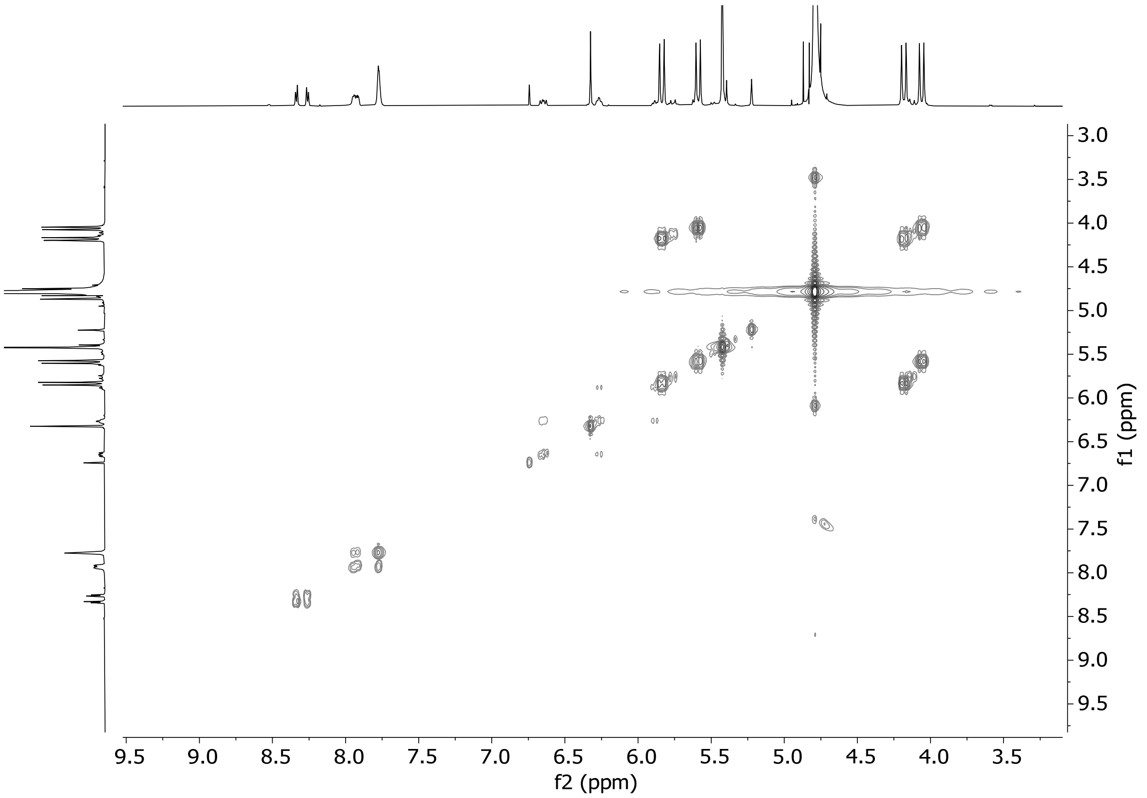

Figure S68 COSY (125 MHz, D_2O) spectrum of $\mathbf{1_a}$·2Br ⊂CB[8].

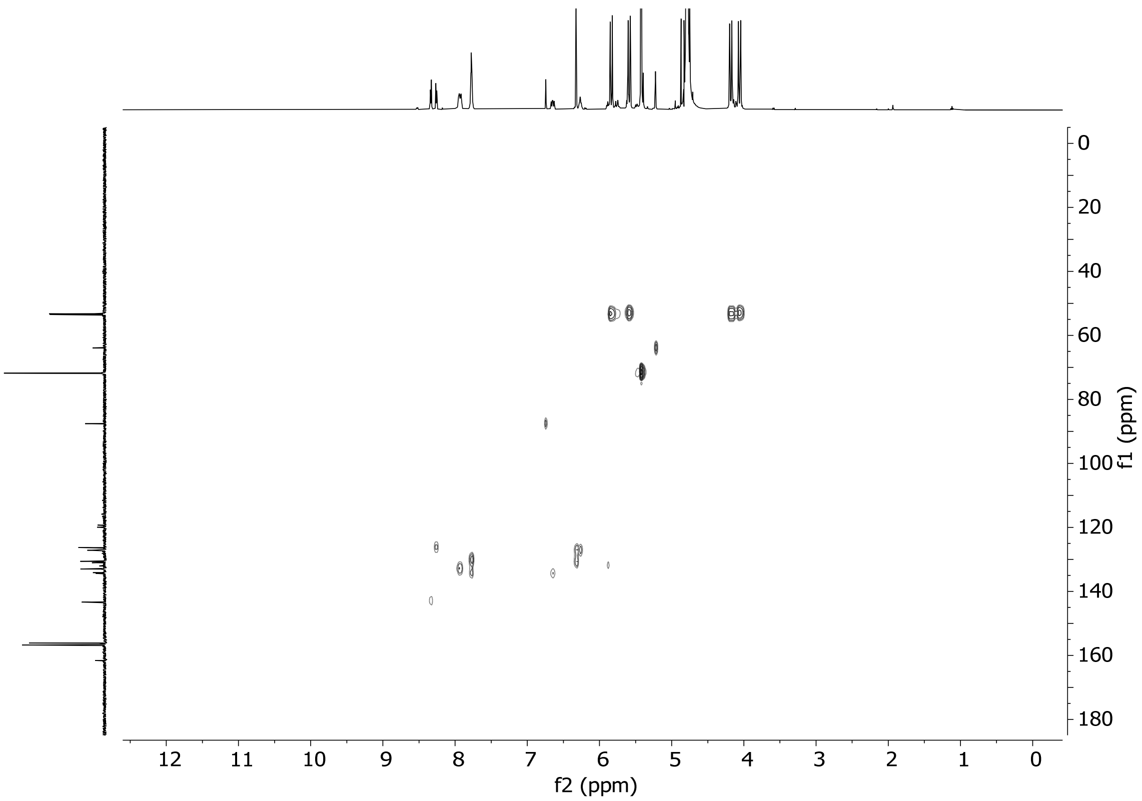

Figure S69 HSQC (125 MHz, D_2O) spectrum of ***1***$_a$*·2Br ⊂CB[8].*

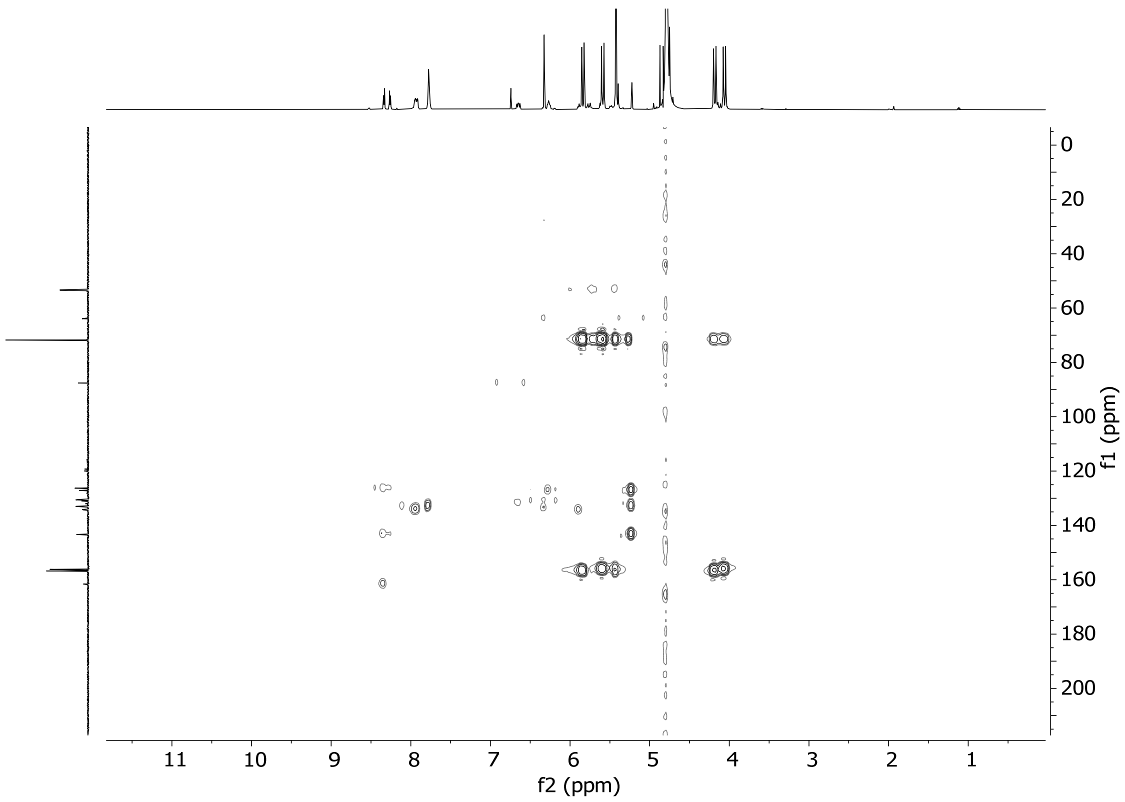

Figure S70 HMBC (125 MHz, D_2O) spectrum of ***1***$_a$*·2Br ⊂CB[8].*

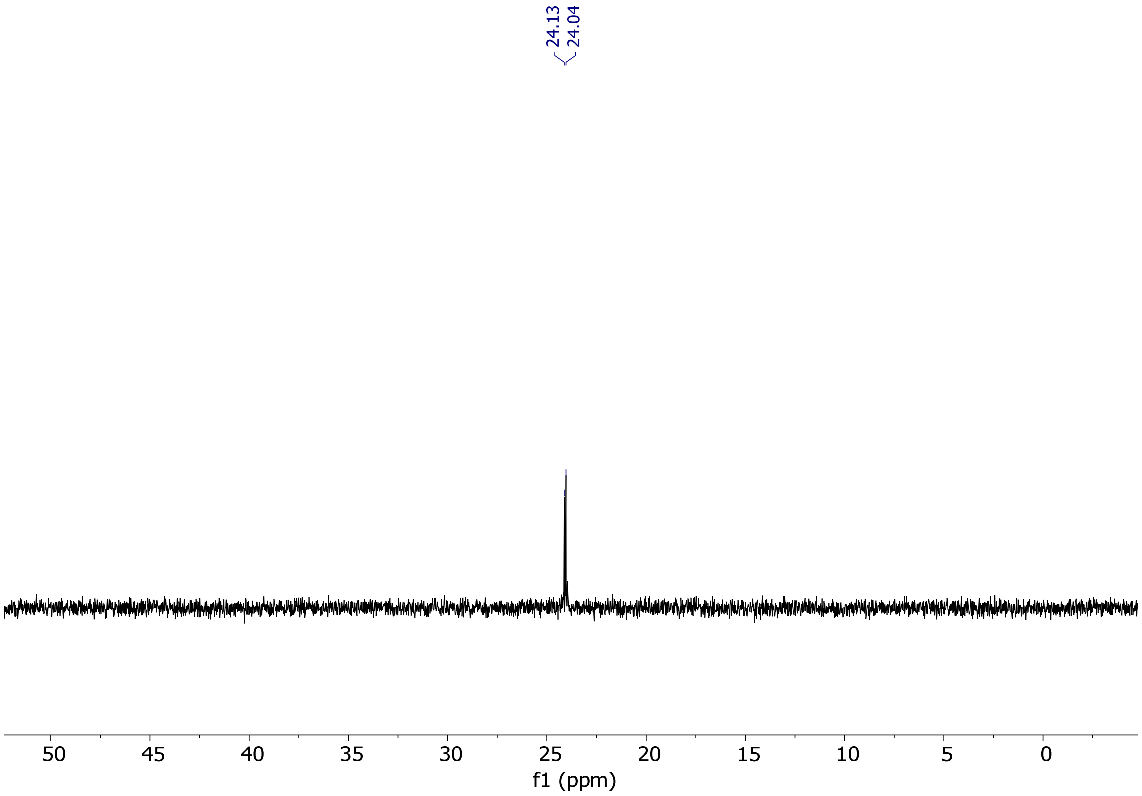

Figure S71 $^{31}P\{^{1}H\}$ NMR (125 MHz, D_2O) spectrum of $\mathbf{1_a}$·2Br ⊂CB[8].

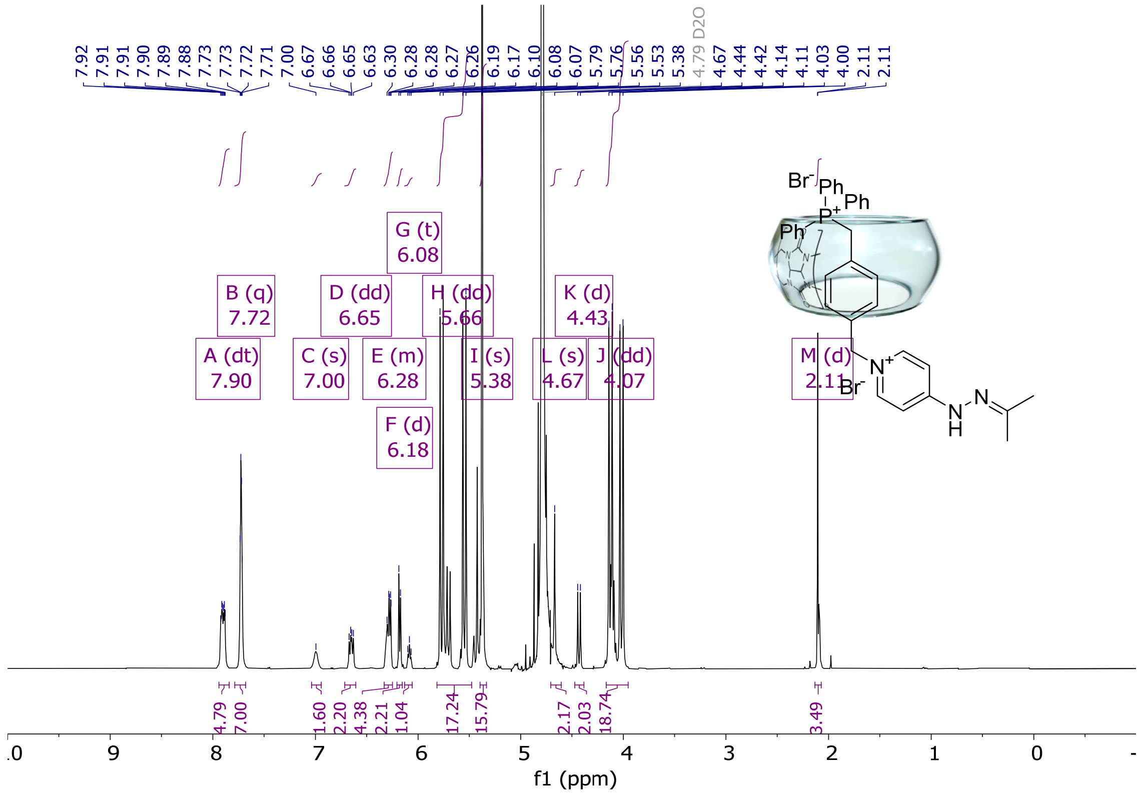

Figure S72 ^{1}H NMR (500 MHz, D_2O) spectrum of $\mathbf{1_b}$·2Br ⊂CB[8].

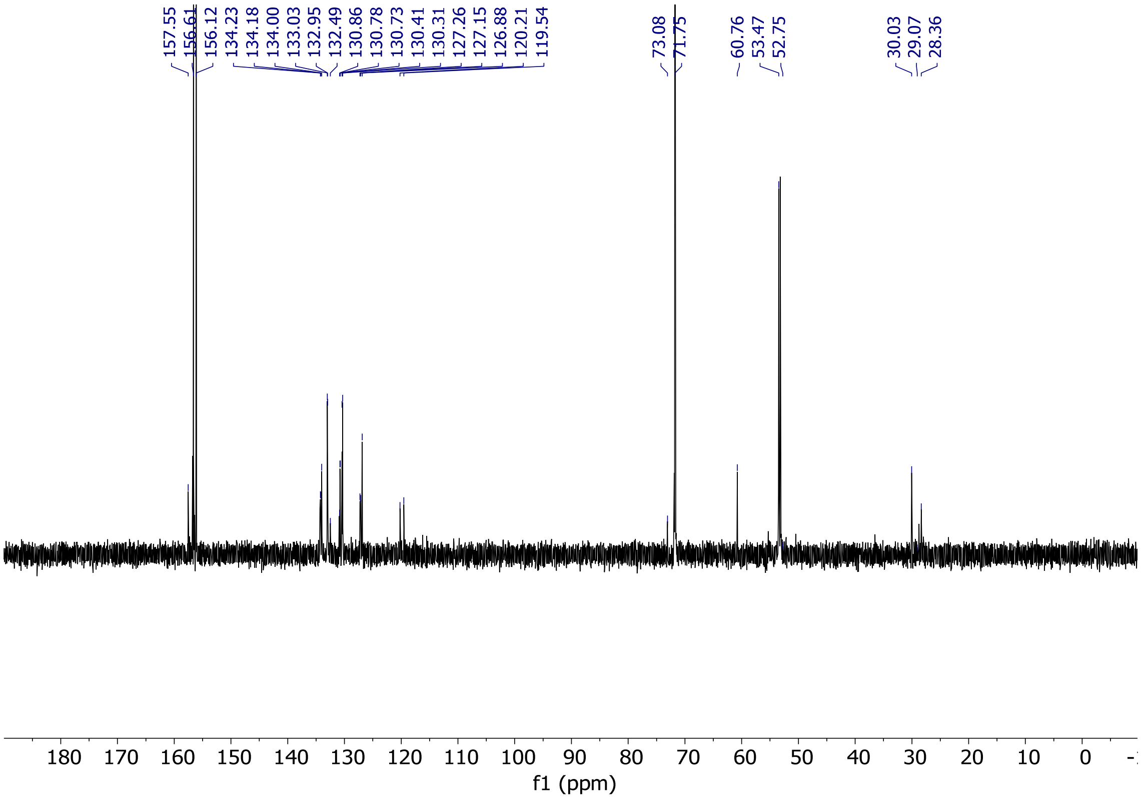

*Figure S73 $^{13}C\{^{1}H\}$ NMR (500 MHz, D_2O) spectrum of **1**$_{b}$·2Br ⊂CB[8].*

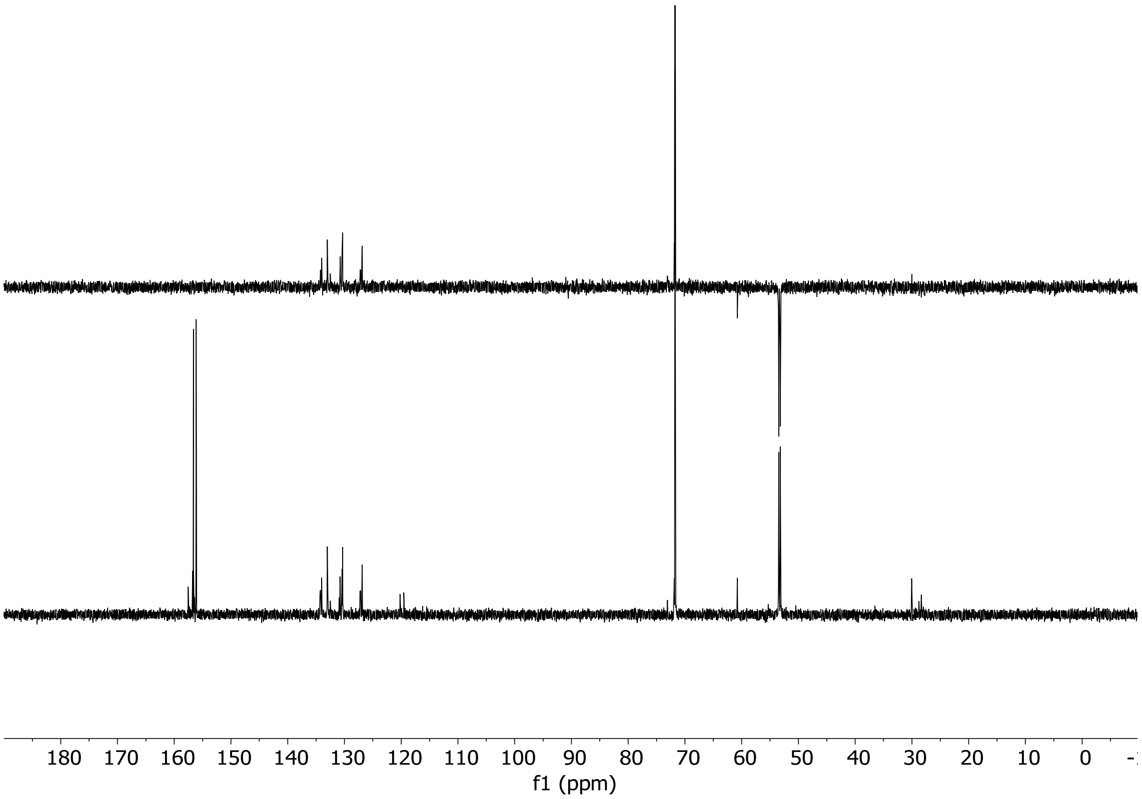

*Figure S74 $^{13}C\{^{1}H\}$ and DEPT 135$\{^{1}H\}$ NMR (500 MHz, D_2O) spectrum of **1**$_{b}$·2Br ⊂CB[8].*

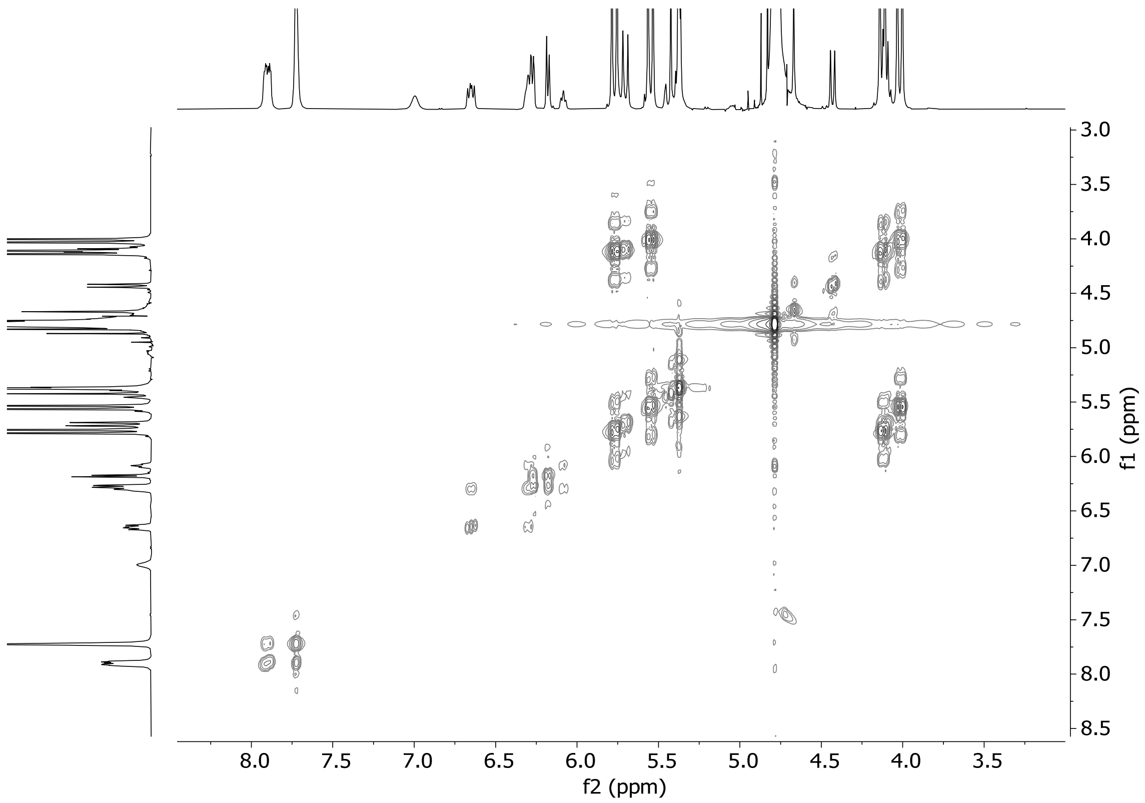

Figure S75 COSY (500 MHz, D_2O) spectrum of $\mathbf{1_b}$·2Br ⊂CB[8].

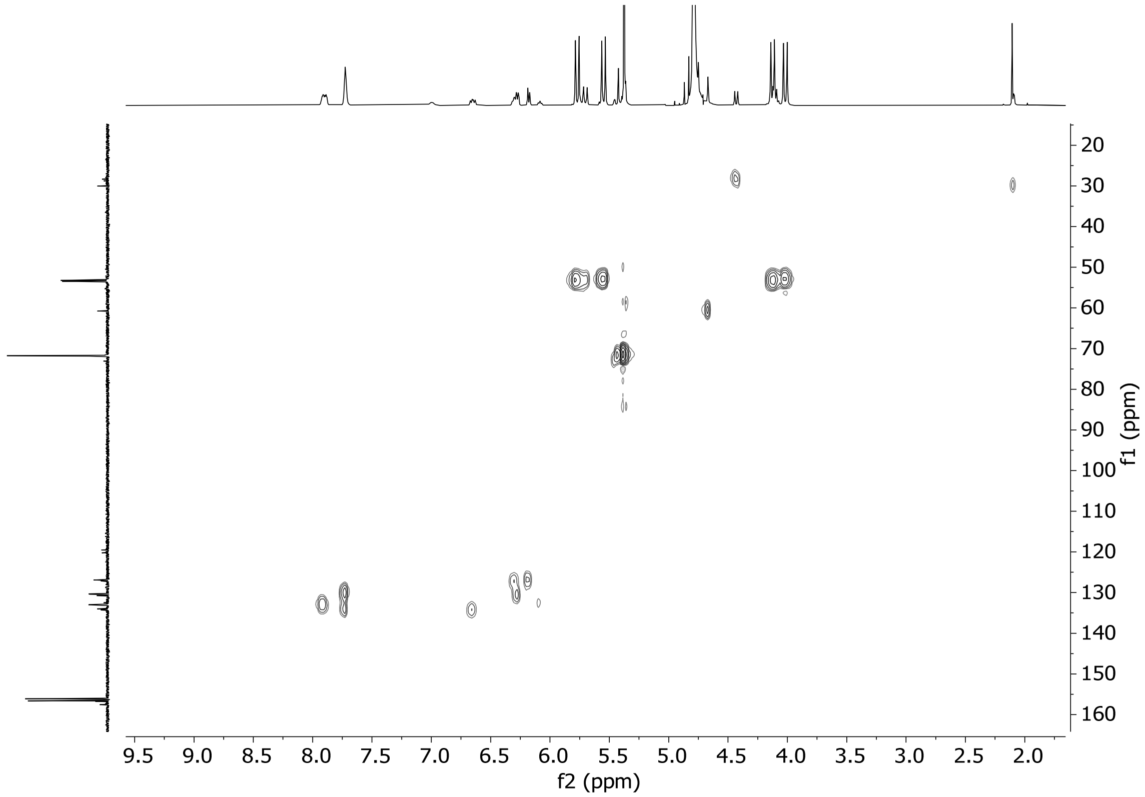

Figure S76 HSQC (500 MHz, D_2O) spectrum of $\mathbf{1_b}$·2Br ⊂CB[8].

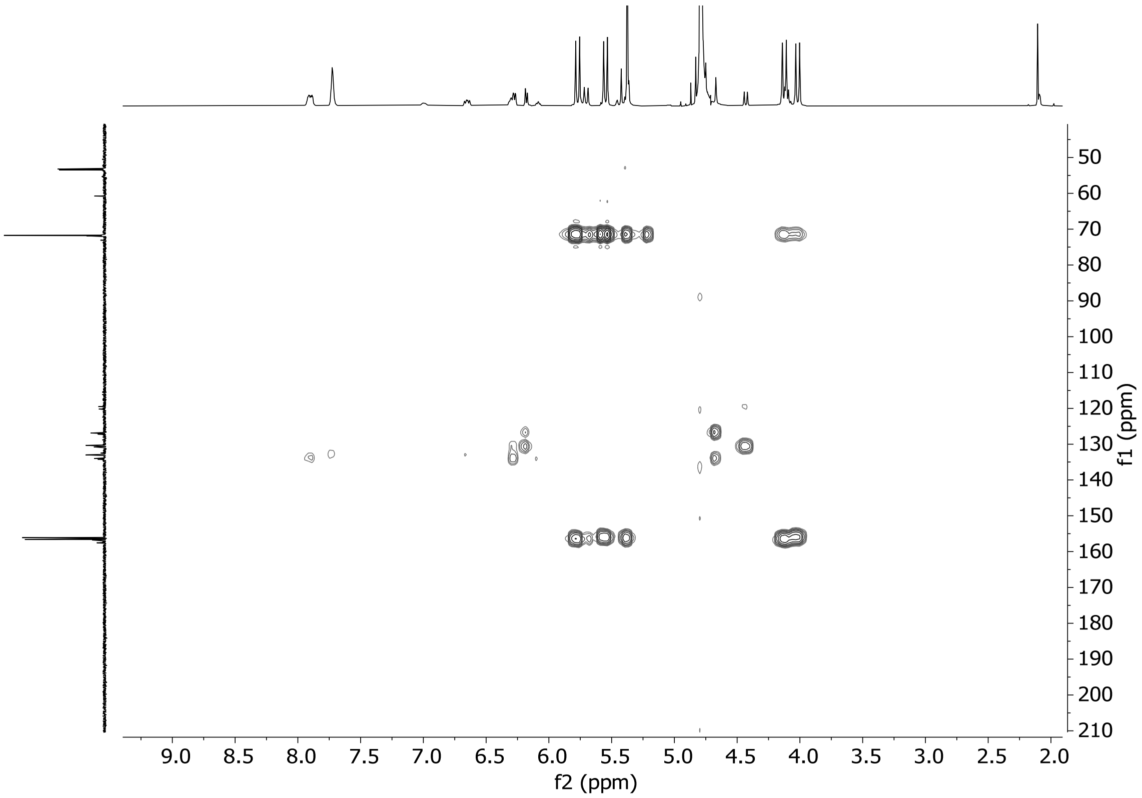

*Figure S77 HMBC (500 MHz, D_2O) spectrum of **1_b**·2Br ⊂CB[8].*

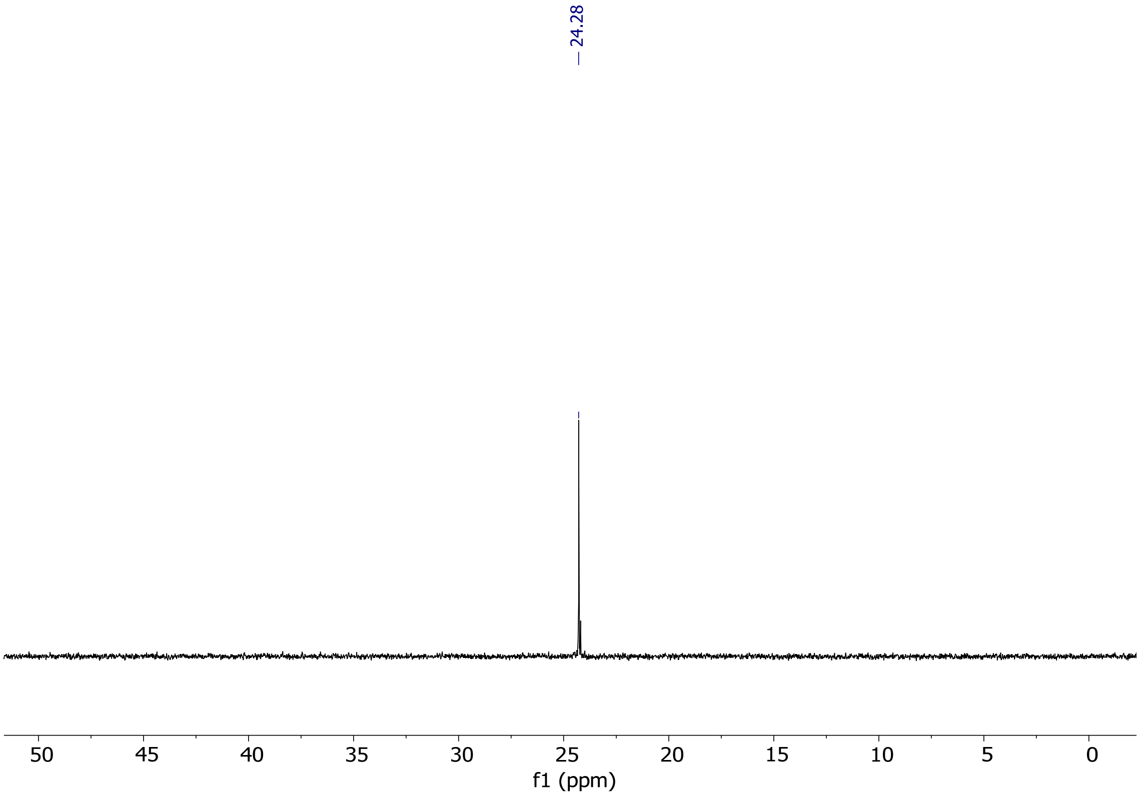

*Figure S78 $^{31}P\{^1H\}$ NMR (500 MHz, D_2O) spectrum of **1_b**·2Br ⊂CB[8].*
